# Using Empirical Performance Data to Source Bluebunch and Snake River Wheatgrass Plant Materials to Restoration Sites in the Eastern Great Basin, USA

**DOI:** 10.1002/ece3.70392

**Published:** 2024-10-23

**Authors:** Thomas A. Jones, Thomas A. Monaco

**Affiliations:** ^1^ Forage and Range Research Laboratory USDA‐Agricultural Research Service Logan Utah USA

**Keywords:** bluebunch wheatgrass, plant adaptation, plant materials, provenance testing, restoration planning

## Abstract

To infer adaptation of plant material, restoration practitioners often consider only surrogate geographic or climatic information. However, empirical biomass data could assist in deciding what material to use where. To test this approach, we transplanted seven bluebunch wheatgrass (BBWG; *Pseudoroegneria spicata*) and five Snake River wheatgrass (SRWG; *Elymus wawawaiensis*) populations to three sites ranging from low to high precipitation (LPPT, MPPT, and HPPT). We measured establishment‐year (2011) biomass at all sites and 2012–16 biomass at MPPT and HPPT. When data were standardized by site, P‐7 and Anatone produced the most BBWG biomass across sites and Wahluke the least in both 2011 and 2012–16, while E‐58X produced the most SRWG biomass and Secar and E‐49X the least in 2011 and 2012–16, respectively. Among BBWG populations in 2011, relative performance of P‐7 (G6 generation) and Goldar increased and Whitmar decreased at wetter sites, while Columbia was stable (high) and Wahluke was stable (low) over sites. Among SRWG populations in 2011, Secar, Secar78, and E‐58X increased at drier sites and Discovery at wetter sites. However, once established, populations of both species were much more similar for trend. In 2012–16, trend somewhat increased for five BBWG populations from MPPT to HPPT, was stable for Wahluke, but declined for Columbia, while all five SRWG populations declined at HPPT. These results suggest that, once established, BBWG is mostly stable across sites, while SRWG is less adapted to wetter sites. In 2012–16, BBWG populations originating at drier (or wetter) sites mostly performed relatively better at MPPT (or HPPT), suggesting adaptation to site. However, in the establishment year (2011), this relationship did not hold, suggesting seedling vigor and immature growth rate play a stronger role than precipitation at the site of origin.

## Graphical abstract

Plant materials can be characterized for adaptation to various potential restoration environments through prior testing. This may be more predictive than surrogate site measures of geographical, climatic, or soils data.

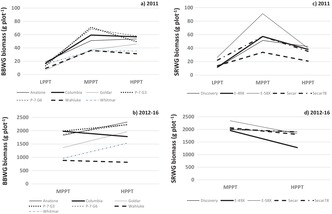

## 1 Introduction

Rangeland ecosystems have undergone dramatic shifts in composition due to climate change, novel disturbance, and annual‐grass invasion that have altered fire regimes and the provision of ecosystem services (Sayre et al. 2017; Crist et al. 2023). While changes in land management can improve land conditions, in some cases, revegetation with suitable plant materials is critical to generate desired conservation benefits (Hardegree et al. 2016; but see Lavin et al. 2013). Plant materials should be able to establish, produce biomass and seed, and persist in areas of intended use (Hardegree et al. 2016). Materials with these attributes will exhibit adaptation to the prevailing pressures of the restoration site (Jones 2013a), and such adaptation should be a primary emphasis when developing and evaluating plant materials for improving revegetation success (Jones 2013a; Jones, Monaco, and Rigby 2015). For example, biomass production is an important trait that integrates the influences of soil type and weather conditions. Biomass has been used to assess adaptation and to serve as a surrogate for fitness, which is much more problematic to measure directly (Younginger et al. 2017). Thus, it has become common practice to compare plant materials for adaptation using biomass as a metric (Kilkenny 2015; Dalgleish et al. 2011; Ying and Yanchuck 2006).

To service rangeland ecosystems in western North America, “workhorse” plant materials have been in production for decades, with their number and species diversity increasing over time (Jones 2019). Such materials offer good performance, display wide adaptation, deliver acceptable seed yields, generate a reliable demand, and sell for a reasonable price. Workhorse plant materials have typically undergone field‐trial evaluation to identify differences in performance, thereby revealing general insights into relative adaptation for general site conditions (Robins et al. 2013; Rigby et al. 2018). Controlled experiments can also identify specific functional traits that impart adaptation to site conditions (Jones, Monaco, and James 2010). Plant materials can be expected to perform differently across precipitation gradients or among differing ecological sites (Robins et al. 2013; Rigby et al. 2018) due to variable functional‐trait expressions (Berger, Ludwig, and Whisson 2020; Galliart et al. 2020; Volis, Mendlinger, and Ward 2002a, 2002b). In arid regions, sandier soils may be more favorable for plant growth than finer textured soils that hold more water (Walter 1964; Noy‐Meir 1973). Thus, a precipitation × soil texture interaction may impact water availability for plant growth (Renne et al. 2019). Considerable attention is being focused on heat tolerance in crop plants due to global warming concerns (Jha, Bohra, and Singh 2014). For example, screening of a reference collection of chickpea (*Cicer arietinum* L.) at two locations in India revealed great genetic variation for heat tolerance and identified genotypes with both heat and drought tolerance (Krishnamurthy et al. 2011).

While local adaptation is common, it is far from universal (Vogel et al. 2018; Hereford 2009; Leimu and Fischer 2008) and should not be assumed without testing (Jones 2013b; Gould 1998). Furthermore, local adaptation may be present but superseded by the high general adaptation (across sites) of a workhorse plant material, making the latter a better indicator of adaptation overall (Gibson and Nelson 2017).

Bluebunch wheatgrass (BBWG; *Pseudoroegneria spicata* [Pursh] À. Löve) and Snake River wheatgrass (SRWG; *Elymus wawawaiensis* J. Carlson & Barkw.) are two cool‐season, perennial Triticeae bunchgrass species that are widely used in rangeland restoration efforts in the Intermountain West (Mukherjee et al. 2011). Because of their superficial resemblance, these species were not recognized as distinct until 1986 (Carlson 1986). The ‘new’ species, *E. wawawaiensis* (Carlson and Barkworth 1997), has been given the common name, SRWG. BBWG and SRWG produce only sterile hybrids and differ for genus (*Pseudoroegneria* vs. *Elymus*), genome constitution (St diploid or less commonly, StSt autotetraploid vs. StH allotetraploid), and usually chromosome number (2*n* = 14 or less commonly 28 vs. 2*n* = 28) (Carlson and Barkworth 1997).

The Intermountain West's native‐seed industry (Jones 2019) currently supplies seed of six BBWG and two SRWG plant materials. These include Whitmar, Goldar, P‐7, Anatone, Columbia, and Wahluke BBWG; and Secar and Discovery SRWG (Table 1). A limited number of additional local populations of BBWG are available, but together they do not represent the entirety of the Intermountain West.

**TABLE 1:** Populations of bluebunch wheatgrass (BBWG) and Snake River wheatgrass (SRWG) planted at three sites.

Species	Name	Release year	Location of origin	Organization	Reference
BBWG	“Whitmar”	1946	Near Colton (Whitman Co.), WA	USDA‐SCS et al. (Pullman, WA)	Ogle, St. John, and Jones (2003)
BBWG	“Goldar”	1989	Mallory Ridge (Asotin Co.; Umatilla National Forest), WA	USDA‐SCS et al. (Aberdeen, ID)	USDA‐SCS et al. (1989)
BBWG	P‐7 Germplasm G3 and G6 generations	2001	25 sites (WA, ID, NV, OR, BC, MT, UT)	USDA‐ARS et al. (Logan, UT)	Jones et al. (2002)
BBWG	Anatone Germplasm	2004	Near Anatone (Asotin Co.), WA	USDA‐FS et al. (Provo, UT)	USDA‐FS et al. (2004)
BBWG	Columbia Germplasm	2015	Adams Co., WA	USDA‐ARS et al. (Logan, UT)	Jones and Mott (2016)
BBWG	Wahluke Germplasm	N/A	Franklin Co., WA	BFI Native Seeds (Moses Lake, WA)	N/A
SRWG	“Secar”	1980	Lewiston Grade, Lewiston (Nez Perce Co.), ID	USDA‐NRCS (Pullman, WA)	USDA‐NRCS (2010)
SRWG	Secar78	N/A	Twickenham (Wheeler Co.), OR	Gabe Williams	N/A
SRWG	“Discovery”	2007	Multiple sites in WA and ID	USDA‐ARS et al. (Logan, UT)	Jones (2008)
SRWG	E‐49X	N/A	SRWG X thickspike wheatgrass (TSWG) 1/4 TSWG	USDA‐ARS (Logan, UT)	N/A
SRWG	E‐58X	N/A	SRWG X TSWG 1/8 TSWG	USDA‐ARS (Logan, UT)	N/A

In this study, we wished to evaluate six BBWG and two SRWG populations being produced by the Intermountain West's native‐seed industry, along with a second generation of P‐7 BBWG and three experimental (unreleased) populations of SRWG. This amounts to a total of seven BBWG and five SRWG plant materials. We assessed their adaptation at three sites spanning three levels of average annual precipitation (AAP) and two ecological site types. The two wetter sites shared a common ecological site (Bonneville big sagebrush), but the third (driest) site was distinct (Wyoming big sagebrush). Ecological sites describe ecological potential and ecosystem dynamics (USDA‐Natural Resources Conservation Service et al. 2024). An ecological site consists of a distinctive kind of land with specific soil and physical characteristics associated with a type and amount of vegetation with characteristic responses to management actions and natural disturbances. Information regarding a particular ecological site is compiled in its Ecological Site Description.

We report four sets of statistics for establishment year (2011) and post‐establishment year (2012–16) biomass: means for individual sites across populations of each species, means of each population at each site, means across sites for each population, and the number of standard deviations above or below the mean for each population at each site. While means across sites provide information regarding general adaptation, means for individual sites provide complementary information regarding specific adaptation to site (Fedriani et al. 2019; Jones 2013b; Kassen 2002).

To elucidate performance of plant materials and to guide their recommended use, we formulated the following six objectives: (1) Determine whether across‐population biomass production of BBWG and SRWG responded similarly across sites; (2) Compare biomass of populations of (a) BBWG and (b) SRWG across sites; (3) Relate each BBWG population's collection‐site AAP to its biomass across sites; (4) Compare older and more recent BBWG releases for biomass; (5) Compare biomass production of two seed‐increase generations (G3 vs. G6) of P‐7 BBWG wheatgrass to identify any genetic shift over generations; and (6) Assess how admixture between SRWG and its closest relative, thickspike wheatgrass (TSWG; *Elymus lanceolatus* [Scribn. & J.G. Smith] Gould), alters adaptation across sites.

## 2 Materials and Methods

We chose three experimental sites representing low (LPPT), medium (MPPT), and high (HPPT) AAP and two ecological sites (Wyoming big sagebrush, Bonneville big sagebrush). Sites included the Lee A. Sharp Experimental Area east of Malta (Cassia Co.), ID (LPPT/291 mm, Wyoming big sagebrush); Nephi Farm south of Nephi (Juab Co.), UT (MPPT/348 mm, Bonneville big sagebrush); and Millville Farm south of Millville (Cache Co.), UT (HPPT/510 mm, Bonneville big sagebrush) (Table 2). April–June mean temperatures among the three sites rank according to latitude, with MPPT (13.44°C; 39.6° N) being warmer than either LPPT (11.54°C; 42.3° N) or HPPT (12.46°C; 41.7° N) (Table 2). For site coordinates, soil classification, and ecological site designation, also see Table 2. The HPPT site exhibits the coarsest soil (highest percentages of sand and silt) and lowest soil‐water content (SWC) at field capacity, while the MPPT site has the finest soil (highest percentages of silt and clay) and highest SWC at field capacity (Table 3). For both variables, LPPT ranks between MPPT and HPPT (Table 3). Soil organic matter of the three sites ranked according to AAP, with LPPT being lowest and HPPT being highest. MPPT and HPPT sites feature soils that are deeper, higher in organic matter, and greater in SWC than the LPPT site (Tables 2 and 3) (Wilder et al. 2019).

**TABLE 2:** Location, precipitation, temperature, soil, and ecological site of three experimental sites.

	Lee Sharp (LPPT)	Nephi (MPPT)	Millville (HPPT)
Location	42.302° N, −113.196° W East of Malta, ID	39.643° N, −111.875° W South of Nephi, UT	41.657° N, −111.815° W South of Millville, UT
30‐year AAP^a^	291	348	510
Actual April–June average ppt (mm)	2011: 76.92 2012: n/a	2011: 154.27 2012–16: 71.28	2011: 271.02 2012–16: 129.01
Temperature^b^	11.54	13.44	12.46
Soil^c^	Declo silt loam 1%–3% slopes (coarse‐loamy, mixed, mesic Xerollic Calciorthids)	Nephi silt loam (fine‐silty, mixed, mesic Calcic Argixerolls)	Ricks gravelly loam 0%–3% slopes (coarse‐loamy over sand or sandy‐skeletal Calcic Haploxerolls)
Ecological site	Wyoming big sagebrush^d^	Bonneville big sagebrush^e^	Bonneville big sagebrush^e^

^a^ Average annual precipitation (mm) over 30 years (1981–2010) (PRISM Climate Group 2022).

^b^ Average April–June temperature (°C) over 30 years (1981–2010) (PRISM Climate Group 2022).

^c^ https://websoilsurvey.sc.egov.usda.gov/App/WebSoilSurvey.aspx.

^d^ RO11XB001ID—Loamy 8–12—Provisional (https://edit.jornada.nmsu.edu/catalogs/esd).

^e^ *A. tridentata* Nutt. ssp. *xbonnevillensis* H. Garrison, L. Shultz, & E.D. McArthur (= *A. tridentata* ssp. *vaseyana* × ssp. *wyomingensis*) (Garrison, Shultz, and McArthur 2013) ecological sites (R028AY310UT—Upland Loam [Bonneville Big Sagebrush] North).

**TABLE 3:** Soil physical properties for three sites derived using the online calculator from the Soil Survey Geographic database (SSURGO) in the Web Soil Survey (WSS) (https://websoilsurvey.nrcs.usda.gov/app/; accessed June 15, 2021). The dominant component aggregation method with a depth range of 0–20 cm was applied for areas of interest scaled at 0.081 ha in each site's center.

	Soil‐water content at −1.5 MPa (dry conditions) (%)	Soil‐water content at −0.033 MPa (field capacity) (%)	Organic matter (%)	Sand (%)	Silt (%)	Clay (%)
Lee Sharp (LPPT)	11.8	27.3	1.5	29.1	53.4	17.5
Nephi (MPPT)	13.5	28.6	2.0	7.0	69.5	23.5
Millville (HPPT)	7.4	18.8	2.5	45.7	41.8	12.5

Plantings of seven BBWG and five SRWG populations (Table 1), plus 27 other BBWG populations not used in this study, were established from 3‐ to 3.5‐month‐old greenhouse‐grown transplants on April 22, 25, 2011 (LPPT), April 20, 2011 (MPPT), and May 5–6, 2011 (HPPT). We used transplant evaluations instead of direct seedings because we wished to evaluate post‐establishment adaptation. Our laboratory has a long history of testing seeded evaluations (Robins et al. 2013; Rigby et al. 2018), but because seedling establishment and post‐establishment adaptation are two distinct phases of the plant's life cycle, they should be evaluated separately. Seeded trials are not ideal for evaluating post‐establishment adaptation because it is confounded by establishment success, which precedes it in time.

The BBWG populations included two cultivars (Whitmar, Goldar) and four pre‐variety germplasms (P‐7, Anatone, Columbia, Wahluke), with P‐7 being represented by G3 and G6 generations of seed increase. The SRWG populations included two cultivars (Secar, Discovery) and three experimental populations (Secar78, E‐49X, E‐58X), with the latter two being admixed (X) with thickspike wheatgrass (TSWG; *Elymus lanceolatus* [Scribn. & J.G. Smith] Gould), SRWG's closest relative (Jones, Wang, and Li 1995).

Plantings at each site were arranged as randomized complete block designs with 12 replications in a 6 × 2 arrangement, and with each replication consisting of 39 plots (populations) in a 3 × 13 arrangement. Each plot consisted of 12 plants planted on 0.4‐m centers in a 6 × 2 arrangement. A single row of Anatone BBWG was transplanted along the perimeter of the planting at each site. Because seedling establishment encompasses a complex assortment of intractable dependent factors, termed a seed ecological spectrum (Saatkamp et al. 2019), we established our sites with transplants instead of seed, as we wished to emphasize post‐establishment adaptation.

In fall 2011 (establishment year), we clipped the 12 plants of each plot at a 5‐cm height (October 17, 2011 at LPPT; October 24, 2011 at MPPT; October, 12–13 2011 at HPPT) and combined the clippings in a paper bag. Similarly, in each summer of 2012–16, we clipped plots at a 10‐cm height (July 18, 2012, August 7, 2013, August 12–13, 2014, August 17, 2015, and August 8, 2016 at MPPT; July 19 and 23, 2012, August 13, 2013, September 6 and 11, 2014, August 27, 2015, and 2016 date unavailable at HPPT). Bags were dried in a forced‐air oven at 60°C for at least 48 h and weighed. Biomass for each plot was totaled over the five post‐establishment years (2012–16) for statistical analysis. As BBWG and SRWG cease growth in mid‐July following seed ripening, the range of harvest dates in 2012–2016 is not problematic. As the LPPT planting was damaged in 2012, only 2011 data could be used.

We used PROC MIXED in SAS 9.4 (SAS Institute, Cary, NC) to perform analysis of variance. Replications, that is, blocks, were considered random, and plant materials and sites were considered fixed. Mean separations were deemed significant at *p* < 0.05 unless otherwise indicated.

**TABLE 4:** Means (and standard errors) for biomass of seven bluebunch wheatgrass populations at three sites for the establishment year (2011) and across post‐establishment years (2012–2016). Population means at a site are based on 12 replicated plots, each with 12 individual plants harvested in bulk. Test statistics may be found in Appendices S1 (2011) and S2 (2012–2016).

Lee Sharp (LPPT)		Nephi (MPPT)		Millville (HPPT)	
Establishment‐year biomass (g plot^−1^)					
Anatone	19.21 (3.68) a^1^	P‐7 G3	71.20 (7.19) a^1^	P‐7 G6	57.94 (5.52) a^1^
Columbia	16.89 (3.61) ab	P‐7 G6	68.55 (8.40) a	Columbia	56.75 (5.49) a
P‐7 G6	16.28 (4.90) ab	Columbia	59.39 (8.85) ab	Anatone	53.51 (4.12) ab
Whitmar	15.60 (2.65) ab	Anatone	51.03 (5.71) b	P‐7 G3	49.35 (4.81) ab
P‐7 G3	13.10 (1.95) bc	Goldar	37.24 (6.43) c	Goldar	45.73 (5.79) b
Wahluke	9.13 (2.31) cd	Wahluke	36.15 (4.36) c	Whitmar	35.40 (3.44) c
Goldar	6.77 (1.13) d	Whitmar	35.60 (5.22) c	Wahluke	31.27 (4.03) c
Mean	13.85		51.31		47.14
Post‐establishment biomass (g plot^−1^)					
		P‐7 G3	1992 (106) a^1^	Anatone	2327 (126) a^1^
		Columbia	1980 (96) a	P‐7 G6	2236 (129) ab
		P‐7 G6	1862 (120) a	P‐7 G3	2229 (91) ab
		Anatone	1830 (65) a	Goldar	1965 (130) bc
		Goldar	1366 (99) b	Columbia	1781 (106) cd
		Whitmar	964 (74) c	Whitmar	1533 (109) d
		Wahluke	883 (75) c	Wahluke	814 (67) e
		Mean	1554		1841

^1^ Not followed by a common letter are significantly different at *p* < 0.05.

## 3 Results

### 3.1 Objective 1: Compare Species by Sites and Sites by Species

**FIGURE 1:**
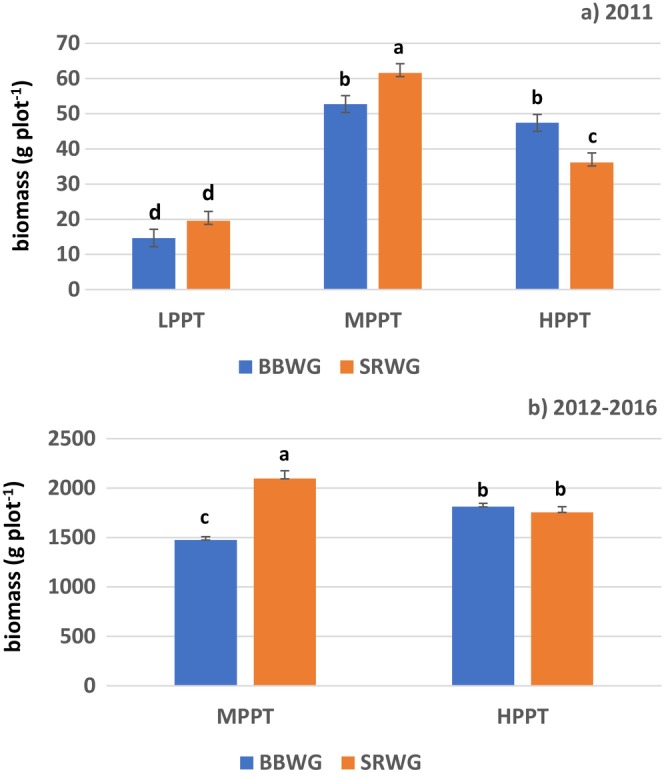
Biomass of bluebunch wheatgrass (BBWG) and Snake River wheatgrass (SRWG) (a) in 2011 (establishment year) at Lee Sharp (LPPT), Nephi (MPPT), and Millville (HPPT) at LPPT, MPPT, and HPPT and (b) summed over 2012–2016 (post‐establishment years) at Nephi (MPPT) and Millville (HPPT) at MPPT and HPPT. Error bars represent ± one standard error.

In 2011, the two species were similar (*p* > 0.05) at LPPT (Figure 1a), while at MPPT, biomass of SRWG exceeded (*p* < 0.05) BBWG. Nevertheless, the SRWG mean relative to BBWG mean was similarly greater at LPPT (21.2%) and MPPT (21.8%). At HPPT, however, BBWG exceeded (*p* < 0.05) SRWG by 31.2%. In 2012–16, SRWG exceeded (*p* < 0.05) BBWG by 41.9% at MPPT, while the species were similar (*p* > 0.05) at HPPT (LPPT not measured) (Figure 1b). Biomass of SRWG across 2012–16 was 19.4% greater (*p* < 0.05) at MPPT than HPPT, while BBWG biomass was 22.7% greater (*p* < 0.05) at HPPT than MPPT (Figure 1b).

When comparing sites for a given species, 2011 SRWG biomass was 214.7% greater (*p* < 0.05) at MPPT than at LPPT and 65.3% greater (*p* < 0.05) at MPPT than at HPPT (Figure 1a). While BBWG biomass was 259.8% greater (*p* < 0.05) at MPPT than at LPPT, it was similar (*p* > 0.05) to BBWG biomass at HPPT. BBWG's 2011 biomass did not decline from MPPT to HPPT, as SRWG's did. Thus, 2012–16 productivity of BBWG relative to SRWG increased from MPPT to HPPT (Figure 1b), as it did in 2011 (Figure 1a).

### 3.2 Objective 2a: Compare BBWG Populations

**FIGURE 2:**
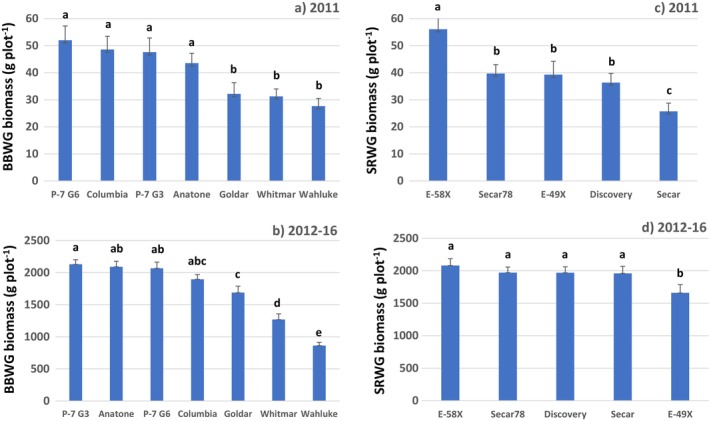
Biomass of (a) seven bluebunch wheatgrass (BBWG) populations averaged across three sites in 2011 (establishment year) and (b) two sites summed over 2012–2016 (post‐establishment years) and (c) five Snake River wheatgrass (SRWG) populations averaged across three sites in 2011 and (d) across two sites summed over 2012–16. Error bars represent ± one standard error.

Averaged across sites, BBWG populations in 2011 segregated (*p* < 0.05) into a high‐biomass group (P‐7 G3/G6, Columbia, Anatone) and a low‐biomass group (Goldar, Whitmar, Wahluke) (Figure 2a). These two groups held for MPPT, but at LPPT, Whitmar moved into the high‐biomass group and P‐7 G3 fell between (*p* > 0.05) the groups, while at the HPPT site, Goldar moved into the high‐biomass group (Table 4).

**TABLE 5:** Means (and standard errors) for biomass of five Snake River wheatgrass populations at three sites for the establishment year (2011) and across post‐establishment years (2012–16). Population means at a site are based on 12 replicated plots, each with 12 individual plants harvested in bulk. Test statistics may be found in Appendices S1 (2011) and S2 (2012–2016).

Lee Sharp (LPPT)		Nephi (MPPT)		Millville (HPPT)	
Establishment‐year biomass (g plot^−1^)					
E‐58X	26.47 (4.13) a^1^	E‐58X	91.07 (10.47) a^1^	Discovery	42.45 (2.44) a^1^
Secar78	22.14 (3.08) a	E‐49X	57.33 (8.34) b	E‐58X	39.94 (3.87) a
Secar	13.89 (4.02) b	Secar78	57.24 (4.98) b	E‐49X	38.05 (5.83) a
E‐49X	11.74 (2.90) b	Discovery	51.39 (4.22) b	Secar78	35.30 (3.07) a
Discovery	11.14 (2.29) b	Secar	33.82 (5.89) c	Secar	20.80 (4.17) b
Mean	17.08		58.17		35.31
Post‐establishment biomass (g plot^−1^)					
		E‐58X	2341 (140) a^1^	Discovery	1902 (134) a^1^
		Secar	2064 (181) ab	Secar78	1901 (98) a
		Secar78	2000 (139) ab	E‐58X	1831 (83) a
		Discovery	1991 (124) ab	Secar	1801 (121) a
		E‐49X	1954 (171) b	E‐49X	1278 (118) b
		Mean	2072		1743

^1^ Not followed by a common letter are significantly different at *p* < 0.05.

For 2012–16, the BBWG populations again segregated into mostly discrete groups (Figure 2b). At MPPT, we observed high‐ (P‐7 G3/G6, Columbia, Anatone) and low‐biomass (Whitmar, Wahluke) groups (*p* < 0.05), with Goldar being intermediate (*p* < 0.05) (Table 4). At HPPT, we observed high‐ (Anatone, P‐7 G3/G6) and intermediate‐biomass (Whitmar, Columbia) groups (*p* < 0.05), with Goldar falling between (*p* > 0.05) the two groups, and Wahluke last (*p* < 0.05) (Table 4). 2012–16 biomass of BBWG plant materials responded differently (*p* < 0.05) from MPPT to HPPT. Biomass of Wahluke and Columbia, which originated from dry sites, decreased 7.8% and 10.1%, respectively, while Anatone, Goldar, and Whitmar, which originated from wet sites, increased 27.2%, 43.9%, and 59.0%, respectively (Table 4).

### 3.3 Objective 2b: Compare SRWG Populations

Averaged across sites, 2011 biomass of E‐58X exceeded (*p* < 0.05) all other SRWG populations, while biomass of Secar fell below (*p* < 0.05) all others (Figure 2c). Furthermore, E‐58X was the highest (*p* < 0.05) or similar (*p* > 0.05) to the highest SRWG at all sites individually, while Secar was lowest (*p* < 0.05) or similar (*p* > 0.05) to the lowest at all sites (Table 5). Discovery produced the numerically lowest biomass at LPPT, while it was numerically highest at HPPT (Table 5).

**FIGURE 3:**
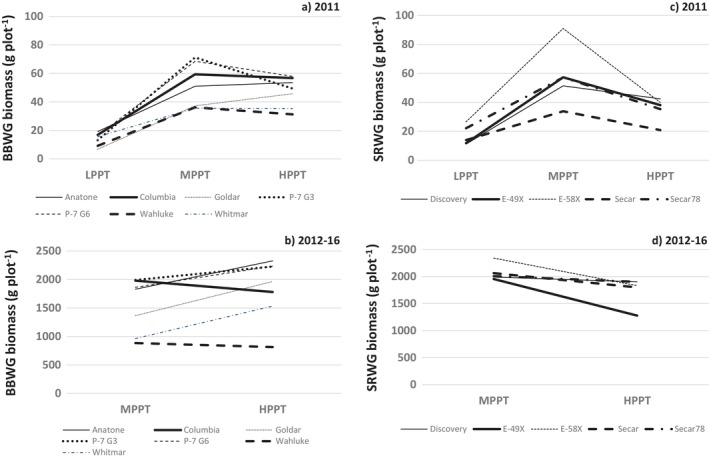
Biomass of (a) seven bluebunch wheatgrass (BBWG) populations at LPPT, MPPT, and HPPT in 2011 (establishment year) and (b) at MPPT and HPPT summed over 2012–16 (post‐establishment years) (standard errors presented in Table 4). Biomass of (c) five Snake River wheatgrass (SRWG) populations at LPPT, MPPT, and HPPT in 2011 and (d) at MPPT and HPPT summed over 2012–16 (standard errors presented in Table 5).

SRWG tended (*p* = 0.10) to produce less 2012–16 biomass at HPPT relative to MPPT, but this varied among populations (Table 5, Figure 3d). This decline was significant (*p* < 0.05) for Secar (12.7%), E‐58X (21.8%), and E‐49X (34.6%), but not (*p* > 0.10) for Secar78 (5.3%) or Discovery (4.5%).

Secar and Discovery, the two released SRWG cultivars, were similar (*p* > 0.05) for 2011 biomass at LPPT, but Discovery was superior (*p* < 0.05) at MPPT and HPPT (Table 5). In 2012–16, in contrast to the establishment year, we found no differences (*p* > 0.05) between Secar and Discovery for biomass at either site (Table 5) or across sites (Figure 2d). In 2011, Secar78 exceeded (*p* < 0.05) Secar at all sites individually (Table 5) and by 54.1% averaged across sites (Figure 2c). However, 2012–16 biomass for Secar78 was similar (*p* > 0.05) to Secar at both sites (Table 5) and averaged across sites (Figure 2d).

### 3.4 Objective 3: Relate BBWG Collection‐Site AAP to Biomass Over Sites

In both 2011 and 2012–16, BBWG populations segregated into high‐ and low‐biomass groups that did not correspond to site AAP (Tables 4 and 6), that is, each biomass group had both high‐ and low‐AAP populations. For example, both the high‐biomass group, Anatone (504 mm AAP) and Columbia (250 mm), and the low‐biomass group, Whitmar (549 mm), Goldar (598 mm), and Wahluke (216 mm), included populations collected from both high‐ and low‐AAP sites (Figure 2a,b). While P‐7 qualified as high biomass (Tables 4 and 6), it was developed from several populations from both high‐ and low‐AAP sites. Thus, it could not be used to address this objective.

### 3.5 Objective 4: Compare Older and More Recent BBWG Releases for Biomass

Biomass in 2011 and 2012–16 was above the mean for P‐7 (released in 2001; +0.64 SD in 2011, +0.76 SD in 2012–16), Anatone (2004; +0.60, +0.75), and Columbia (2015; +0.68, +0.39) (Table 6). However, older releases or non‐releases, for example, Whitmar (1946; −0.40, −0.76), Goldar (1989; −0.72, 0.00), and Wahluke (source‐identified; −0.94, −1.18), fell at or below the mean.

### 3.6 Objective 5: Identify any Genetic Shift in P‐7 Seed Increase

In 2011, P‐7 G3 and G6 neither differed (*p* > 0.05) at any site (Table 4), nor when averaged across sites (Figure 2a). The same was true for 2012–16 biomass (Table 4, Figure 2b).

**TABLE 6:** Positions and trends for relative biomass of seven bluebunch wheatgrass (BBWG) and five Snake River wheatgrass (SRWG) populations for three sites (LPPT, MPPT, and HPPT; low, mid, and high precipitation) and trends across the three sites in the establishment year (2011) and across post‐establishment years (2012–2016). Based on the area under a normal curve, position ranges from high (top third; > 0.43 SD above the mean of 39 populations) to mid (middle third; < 0.43 SD above the mean and < 0.43 SD below the mean) to low (bottom third; > 0.43 SD below the mean). Trend describes the change in site position from LPPT to MPPT to HPPT. See Figure 3 for graphs of unstandardized data.

	Lee Sharp (LPPT)	Nephi (MPPT)	Millville (HPPT)	Overall mean	Overall position	Site positions^b^	Trend over sites^c^
	Standard deviations^a^						
	Above (+) or	Below (−)	Mean^a^				
BBWG 2011							
P‐7 G6	0.35	1.12	1.04	0.84	High	Mid/high/high	Rises
Columbia	0.47	0.61	0.95	0.68	High	High/high/high	Stable
Anatone	0.96	0.14	0.71	0.60	High	High/mid/high	Sags at MPPT
P‐7 G3	−0.32	1.26	0.39	0.44	High	Mid/high/mid	Peaks at MPPT
Whitmar	0.20	−0.71	−0.68	−0.40	Mid	Mid/low/low	Falls
Goldar	−1.64	−0.62	0.11	−0.72	Low	Low/low/mid	Rises
Wahluke	−1.14	−0.68	−0.99	−0.94	Low	Low/low/low	Stable
BBWG 2012‐16							
P‐7 G3	—	0.82	0.79	0.81	High	−/High/high	Stable
Anatone	—	0.52	0.97	0.75	High	−/High/high	Stable
P‐7 G6	—	0.58	0.81	0.70	High	−/High/high	Stable
Columbia	—	0.79	−0.02	0.39	Mid	−/High/mid	Falls
Goldar	—	−0.32	0.31	0.00	Mid	−/Mid/mid	Stable
Whitmar	—	−1.05	−0.47	−0.76	Low	−/Low/low	Stable
Wahluke	—	−1.20	−1.17	−1.18	Low	−/Low/low	Stable
SRWG 2011							
E‐58X	2.47	2.37	−0.33	1.50	High	High/high/mid	Falls
Secar78	1.57	0.49	−0.69	0.46	High	High/high/low	Falls
E‐49X	−0.60	0.49	−0.48	−0.20	Mid	Low/high/low	Peaks at MPPT
Discovery	−0.73	0.16	−0.14	−0.24	Mid	Low/mid/mid	Rises
Secar	−0.15	−0.81	−1.79	−0.92	Low	Mid/low/low	Falls
SRWG 2012–16							
E‐58X	—	1.45	0.07	0.76	High	−/High/mid	Falls
Secar78	—	0.83	0.20	0.52	High	−/High/mid	Falls
Discovery	—	0.81	0.20	0.51	High	−/High/mid	Falls
Secar	—	0.95	0.02	0.49	High	−/High/mid	Falls
E‐49X	—	0.75	−0.93	−0.09	Mid	−/High/low	Falls

^a^ Mean and SD based on all 39 populations.

^b^ Positions at LPPT, MPPT, and HPPT, respectively. Dash indicates no data for LPPT for 2012–16.

^c^ Trends from LPPT to MPPT to HPPT, that is, from lower to higher precipitation.

### 3.7 Objective 6: Assess Impact of TSWG Admixture on SRWG

In 2011, E‐58X displayed a higher biomass mean averaged across sites than any other SRWG population (Figure 2c). Its superiority in 2011 was particularly evident at MPPT (Table 5, Figure 3c). However, E‐58X was similar (*p* > 0.10) to most other SRWG populations at both MPPT and HPPT for 2012–16 (Table 5). E‐49X was the only exception, being inferior (*p* < 0.05) to E‐58X at all combinations of site and year except HPPT in 2011, where they were similar (*p* > 0.05) (Table 5).

Post‐establishment biomass of E‐58X exceeded (*p* < 0.05) E‐49X at both MPPT and HPPT (Table 5) and averaged across sites (Figure 2d). At MPPT, E‐49X performed well prior to 2015 but poorly for the last 2 years, while at HPPT, E‐49X performed poorly in all five post‐establishment years (data not shown). In contrast, E‐58X performed well for all years at MPPT, while poorly only in the last year at HPPT (data not shown).

## 4 Discussion

Restoration practitioners often struggle with choosing plant materials for inclusion in a restoration seeding mix (Johnson et al. 2015). For the relatively few workhorse species (Erickson 2008), several plant materials may be available in commerce (Jones 2019), though availability is often a limiting factor (Rowe 2010; Waters and Shaw 2003). Performance data may characterize establishment and productivity and suggest which plant material is likely to persist on the land and in the marketplace. Ideally, multi‐year data from a variety of sites should be routinely collected, interpreted, published, and distributed to practitioners (Robins et al. 2013; St. Clair et al. 2013; Davies et al. 2015; Rigby et al. 2018; Clements, Harmon, and Blank 2022), who are likely to value such explanatory information for planning their restoration projects.

Plant adaptation may be a function of various traits, such as biomass, reproductive output, germinability, and survivorship (Sambatti and Rice 2006). In our dataset, survivorship was consistently over 95%, which may relate to the fact that vigorous greenhouse‐grown transplants were used. Thus, biomass was deemed a better measure of adaptation to site than survivorship. Mortality would be most likely to appear at LPPT, but this site was inadvertently damaged and subsequently terminated. Our species display high germination and minimal seed dormancy (Young, Eckert, and Evans 1981; Kitchen and Monsen 1994; Rigby et al. 2018). For these reasons, we employed above‐ground biomass as a measure of adaptation, as have several others (Casler et al. 2004; Gustafson, Gibson, and Nickrent 2004; Vogel et al. 2018). Younginger et al. (2017) argued that biomass is a reliable estimate of fitness when making comparisons within a species of the same age class. While we recognize that smaller plants often typify low‐resource environments, we argue that when populations are compared at a common site, relative biomass among populations over time is a good measure of relative adaptation to that site. Here, we not only make comparisons among populations of two species separately, but we also compared the species based on their means across populations.

### 4.1 Objective 1: Matching Species to Site

Productivity in 2011 was much lower at LPPT (292 mm AAP) than at the two wetter sites (Figure 1a). Robins et al. (2013) reported that establishment and 3‐year persistence of cool‐season grasses were poor at sites receiving < 310 mm AAP. When comparing MPPT and HPPT, however, environmental factors in addition to limiting precipitation appear to have played a role in biomass production, as SRWG biomass decreased from MPPT to HPPT. On the other hand, BBWG biomass at MPPT and HPPT was similar (*p* > 0.05) in 2011 and increased (*p* > 0.05) from MPPT to HPPT in 2012–16 (Figure 1).

While BBWG and SRWG are both cool‐season bunchgrasses that occupy similar ecological niches in our semi‐arid region, they vary for functional traits (Mukherjee et al. 2011, 2015; Ray‐Mukherjee et al. 2011). Interestingly, Secar SRWG, released in 1980, was unwittingly used as a surrogate for BBWG before SRWG was formally recognized as a distinct species (Carlson and Barkworth 1997).

At MPPT, in both 2011 and 2012–16, SRWG exceeded (*p* < 0.05) BBWG for biomass production (Figure 1). In 2011 at LPPT, SRWG biomass exceeded (*p* < 0.05) that of BBWG by a similar percentage as at MPPT, though at LPPT, the difference was not significant (*p* > 0.05) (Figure 1). Thus, SRWG seems to have been favored at lower precipitation sites relative to BBWG. Warmer temperatures may also have favored SRWG at MPPT, though not at LPPT, the coolest site, as BBWG is more sensitive to high‐temperature stress than SRWG (He, Monaco, and Jones 2018; Jones, Bell, and Monaco 2021).

At MPPT, SRWG may have also been favored by its finer roots, that is, higher specific root length (SRL) (Mukherjee et al. 2011). High SRL is associated with fine‐textured soils (Xie et al. 2012), such as at MPPT (Tables 2 and 3), though not with low available water (Ostenon et al. 2007; Mukherjee et al. 2011; Reisner et al. 2013). Besides root proliferation, high SRL enhances root length density and concomitant water extraction, though root lifespans are shorter and turnover rates are greater (Eissenstat and Caldwell 1988, 1989; Eissenstat 1991). Relative to BBWG, these SRWG traits confer greater adaptation to a finer‐textured soil that retains moisture longer.

The coarse‐textured soil at HTTP (Tables 2 and 6) may have provided a greater opportunity for BBWG's thicker roots, that is, lower SRL, to extract soil moisture due to less resistance, less negative soil matric potential (Tuller and Or 2004), and percolation of water away from the soil surface (Noy‐Meir 1973; Sala et al. 1988). This may partially explain previous reports that BBWG performs better on coarse‐textured than fine‐textured soils (Madsen et al. 2012; Wilder et al. 2019). Likewise, soil texture might explain why 2012–16 biomass of BBWG increased from MPPT to HPPT, while that of SRWG declined. BBWG might also have been favored over SRWG at HPPT because this site features cooler temperatures than MPPT (Table 1). Thus, BBWG's greater susceptibility to heat (He, Monaco, and Jones 2018) would be less problematic at HPPT than at MPPT.

### 4.2 Objective 2a: Matching BBWG Plant Material to Site

Our data offer some insight into the relationship between collection‐site AAP and adaptation across test sites varying for AAP. Post‐establishment (2012–16) biomass declined slightly from MPPT to HPPT for two populations from low‐AAP sites, Columbia (7.8%; 250 mm) and Wahluke (10.1%; 216 mm) (Table 4; Figure 3b), suggesting that these populations were unable to capitalize on HPPT's greater precipitation. On the other hand, biomass of Anatone (504 mm), Goldar (598 mm), and Whitmar (549 mm), all originating from considerably wetter sites, increased 27.2%, 43.9%, and 59.0%, respectively, from MPPT to HTTP (Table 4, Figure 3b). These post‐establishment data suggest that populations differ in site adaptation based on the AAP of their collection sites. However, the 2011 establishment‐year data did not reveal such a connection between collection‐site and test‐site AAP, suggesting that, in the establishment year, traits relating to immature growth supersede site adaptation.

Wahluke is of particular interest, being collected at only 216 mm AAP (PRISM 2022). Its relative biomass was very low and stable across sites (Table 4). BBWG populations from more‐arid sites in the Great Basin have been reported to be smaller and less vigorous than released populations from the Columbia Plateau Level III ecoregion of the Pacific Northwest (St. Clair et al. 2013). In general, biomass is positively correlated with fitness (Younginger et al. 2017), which would suggest that Wahluke has low fitness. However, trade‐offs have been documented between trait plasticity and resource conservation (Power et al. 2019) and between trait plasticity and fitness homeostasis (Davidson, Jennions, and Nicotra 2011). Thus, Wahluke's low plasticity may relate to high resource conservation and fitness homeostasis rather than to an inherent lack of fitness. In addition, by reducing fuels, Wahluke's low biomass may make it desirable for seeding on rangelands prone to wildfire.

We note that BBWG plant materials tended to consistently segregate into lower biomass (Goldar, Whitmar, and Wahluke) and higher biomass (P‐7, Anatone, Columbia) groups across sites (Figure 2a,b), as well as at individual site‐year grouping (2011, 2012–16) combinations (Table 4). However, at the same time, we saw variation for phenotypic plasticity, as mentioned above. Thus, Whitmar produced more biomass than expected at LPPT in 2011, as did Goldar at HPPT in both 2011 and 2012–16. Together, these observations suggest that biomass of a plant material at a site is jointly determined by genetic growth potential (general adaptation) and phenotypic plasticity in response to environment (specific adaptation).

### 4.3 Objective 2b: Matching SRWG Plant Material to Site

In general, relative biomass of SRWG populations fell at higher AAP sites, as SRWG fared poorer relative to BBWG from MPPT to HPPT (Figure 1, Table 6). However, we report two noteworthy exceptions. In 2011, E‐49X peaked at MPPT relative to the LPPT and HPPT sites, while Discovery's relative biomass rose from LPPT to MPPT/HPPT (Table 6, last column). Nevertheless, for 2012–16 biomass, all five SRWG populations fell from MPPT to HPPT (Table 6, last column). Both establishment‐year and post‐establishment data suggest that E‐58X, Secar78, and Secar are better adapted to drier sites, while Discovery performed relatively better at wetter sites in 2011 and relatively better at drier sites in 2012–16. E‐49X's best relative performance, on the other hand, was at MPPT.

Discovery's 2011 performance improved relative to Secar from drier to wetter sites (Table 6), suggesting the former is better adapted to wetter sites for establishment. Previous research (Jones 2008) also found better (*p* < 0.05) stand establishment for Discovery across two sites in the Intermountain West. While here we found no differences in biomass between Secar and Discovery for 2012–16 (Table 5), the previous study (Jones 2008) reported greater biomass for Discovery for 3 years post‐establishment. Mendola et al. (2015) reported that a southern Illinois ecotype of big bluestem produced more above‐ground biomass at an intermediate site in eastern Kansas and a wetter site in southern Illinois than at a drier site in central Kansas. However, a central Kansas ecotype's biomass did not respond to increased precipitation at the wetter sites, suggesting limited plasticity.

The relative performance of Secar and Secar78 is of interest based on reports that Secar78, a population collected in the vicinity of Secar's collection site, but never released, displays superior growth over Secar (rancher Gabe Williams, Twickenham, OR, pers. comm.). Our data support the superiority (*p* < 0.05) of Secar78 in the critical establishment year (2011), as evidenced by Secar78's higher relative biomass at all three sites (Table 5). However, this superiority did not extend post‐establishment (Table 5), as has been reported from an arid location in central Oregon (rancher Gabe Williams, Twickenham, OR, pers. comm.).

We noted that SRWG may perform better than BBWG at sites with finer soils, as well as at drier and warmer sites. Climate change is expected to create increasingly inhospitable environments for BBWG in our region, especially in the Snake River Plain (Kilkenny 2015). Based on our findings of better SRWG performance relative to BBWG at the warmer and drier MPPT location (relative to HPPT), SRWG may become increasingly preferred over BBWG in the future.

### 4.4 Objective 3: Corresponding BBWG Biomass to Collection‐Site AAP

BBWG populations showed a lack of close correspondence between collection‐site AAP and biomass among sites, though a general trend of higher biomass at higher‐AAP sites may be present. Matching a candidate plant material's seed zone to that of the restoration site is a commonly recommended method of choosing a plant material (Johnson et al. 2010; Bower, St. Clair, and Erickson 2014). However, we contend that plant materials are best evaluated with actual field trials rather than on presumed adaptation based on extrapolation from geographic or climatic data (Jones 2019).

### 4.5 Objective 4: Compare Older and More Recent Releases for Biomass

Relative biomass was greater for twenty‐first century releases (P‐7, Anatone, Columbia) than for both twentieth century releases (Whitmar, Goldar) and unreleased Wahluke. These data suggest that developers of native grass materials have been successful over time in releasing materials that are better adapted to rangeland conditions in the Intermountain West. While the data of St. Clair et al. (2013) suggest they may be more similar than what we report here, they presented no statistical separation.

### 4.6 Objective 5: Evidence for Genetic Shift in P‐7 BBWG

The lack of evidence for increased biomass resulting from genetic shift over three generations of seed increase under irrigated and fertilized conditions suggests that either genetic shift did not occur or phenotypic plasticity overrode genetic shift as the predominant response to seed‐increase environments (Agosta and Klemens 2008; Fraser et al. 2011; Richter et al. 2012; Oduor, Leimu, and Van Kleunen 2016). Thus, any negative consequences of genetic shift away from adaptation to the home site appear to have been avoided.

### 4.7 Objective 6: Assessing the Impact of TSWG Admixture on SRWG Adaptation

E‐58X appeared to perform best relative to other SRWG populations in the establishment year at LPPT and MPPT, though not at HPPT (Table 5). E‐58X's exceptional relative biomass production in 2011 may have resulted from the inclusion of a small amount (1/8) of TSWG germplasm. This species has considerably greater establishment‐year growth than SRWG (Bell, Jones, and Monaco 2019), though it must be noted that E‐49X, a hybrid material with 1/4 TSWG germplasm, displayed only mediocre biomass in 2011. E‐58X was similar in biomass to the non‐hybrid SRWG populations in 2012–16 (Table 5), so it would not be expected to be especially competitive.

## 5 Conclusion

A major purpose of this work was to infer adaptation of BBWG and SRWG to sites. Overall, our data here suggest that SRWG may be favored over BBWG at sites that are drier, warmer, and feature finer soil texture. Previous work (Jones, Bell, and Monaco 2021) had suggested that SRWG displays traits that facilitate growth, while BBWG displays traits that conserve resources, suggesting adaptation to more‐fertile and less‐fertile sites, respectively.

The newer higher‐performing BBWG populations (P‐7, Anatone, Columbia) were similar (*p* > 0.05) for establishment‐year relative biomass overall, but Anatone displayed the most (*p* < 0.05) relative biomass at LPPT and P‐7 at MPPT (Table 4). For post‐establishment biomass, differences were small at MPPT, but Columbia appeared to be less adapted (*p* < 0.05) at HPPT (Table 4). Thus, we can recommend all three plant materials for drier and intermediate sites, but Columbia should be avoided at wetter sites.

Among SRWG populations, Secar78 exceeded (*p* < 0.05) Secar in the establishment year at all sites, though they were similar (*p* > 0.05) across 2012–16 (Table 5). Of the two released SRWG populations, Discovery performed better (*p* < 0.05) than Secar at MPPT and HPPT in the establishment year, but they were similar (*p* > 0.05) at LPPT. Over post‐establishment years, however, the two populations were no different (*p* > 0.05) (Table 5). Of the five SRWG populations, E‐58X displayed the greatest (*p* < 0.05) establishment‐year biomass at LPPT and MPPT, as well as for post‐establishment year biomass at MPPT. Thus, it may have potential for drier sites.

## Author Contributions

**Thomas A. Jones:** conceptualization (equal), data curation (lead), formal analysis (lead), investigation (equal), writing – original draft (equal), writing – review and editing (equal). **Thomas A. Monaco:** conceptualization (equal), investigation (equal), writing – original draft (equal), writing – review and editing (equal).

## Conflicts of Interest

The authors declare no conflicts of interest.

## Supporting information


Appendix S1

## Data Availability

Data are available at https://doi.org/10.15482/USDA.ADC/25029620.
