# Supplementary material for: Using Empirical Performance Data to Source Bluebunch and Snake River Wheatgrass Plant Materials to Restoration Sites in the Eastern Great Basin, USA
Source: Ecol Evol. 2024 Oct 23;14(10):e70392. doi: 10.1002/ece3.70392 (PMC11499301; doi:10.1002/ece3.70392)
Supplement: Supplementary file 1 — Appendix S1 [file ECE3-14-e70392-s001.docx]

LIST OF APPENDICES

Appendix S1. SAS code (TOM289A2.sas) and output for least-squares means (LSmeans) for transformed biomass (TDW_F 11) at three locations in 2011. To generate back-transformed LSmeans, apply the formula DW_F11 = ((TDW_F11/4) + 1)^4^. Bluebunch wheatgrass statistics are presented in Table 4, and Snake River wheatgrass statistics are presented in Table 5.

Appendix S2. SAS code (TOM289S1.sas) and output for least-squares means (LSmeans) for transformed total biomass (TTOTDW) at two locations across 2012-2016. To generate back-transformed LSmeans, apply the formula TOTDW = ((TTOTDW/2) + 1)^2^. Bluebunch wheatgrass statistics are presented in Table 4, and Snake River wheatgrass statistics are presented in Table 5.

Appendix S3. SAS code (TOM289ZZH.sas) and output for standard errors (s.e.) for biomass in 2011 (DW_F11) at three locations and across 2012-2016 (TOTDW) at two locations. Bluebunch wheatgrass statistics are presented in Table 4, and Snake River wheatgrass statistics are presented in Table 5.

Appendix S1. SAS code (TOM289A2.sas) and output for least-squares means (LSmeans) for transformed biomass (TDW_F 11) at three locations in 2011. To generate back-transformed LSmeans, apply the formula DW_F11 = ((TDW_F11/4) + 1)^4^. Bluebunch wheatgrass statistics are presented in Table 4, and Snake River wheatgrass statistics are presented in Table 5.

FILENAME SCREEN DDE 'EXCEL|Sheet1!R5C1:R1408C8';

**DATA** ONE;

INFILE SCREEN LRECL=**5000**;

INPUT LOC $ REP PLOT NO_PLTS ENTRY $ DW_F11 DW_12 DW_13;

OPTION PAGESIZE=**5000**; LRECL=**1000**;

%include 'c:\pdmix8002.SAS';

**DATA** ONE; SET ONE;

DW_F11=DW_F11*(**12**/NO_PLTS);

**DATA** NEPHI; SET ONE; IF LOC='Nephi';

TDW_F11=((DW_F11****0.25**)-**1**)/**0.25**;

IF REP=**1** AND PLOT=**1** THEN TDW_F11=**.**;

IF REP=**2** AND PLOT=**27** THEN TDW_F11=**.**;

IF REP=**7** AND PLOT=**39** THEN TDW_F11=**.**;

**DATA** MV; SET ONE; IF LOC='Mville';

TDW_F11=((DW_F11****0.25**)-**1**)/**0.25**;

**DATA** SHARP; SET ONE; IF LOC='Sharp';

**DATA** SHARP; SET SHARP;

TDW_F11=((DW_F11****0.25**)-**1**)/**0.25**;

**PROC** **MIXED** DATA=SHARP;

TITLE1 'LEE SHARP DRY-MATTER';

TITLE2 'FALL 2011';

CLASS REP ENTRY;

MODEL TDW_F11 = ENTRY/OUTP=RESID5;

RANDOM REP;

LSMEANS ENTRY/PDIFF;

ODS OUTPUT DIFFS=EEE LSMEANS=FFF;

ODS EXCLUDE DIFFS;

**RUN**;

%***PDMIX800*** (EEE,FFF,ALPHA=**0.05**,SORT=YES)

%***PDMIX800*** (EEE,FFF,ALPHA=**0.10**,SORT=YES)

QUIT;

**RUN**;

**PROC** **UNIVARIATE** DATA=RESID5 NORMAL PLOT;

VAR RESID;

HISTOGRAM RESID/NORMAL NOFRAME;

PROBPLOT RESID/NORMAL NOFRAME;

**RUN**;

**PROC** **TRANSREG** DATA=SHARP;

MODEL BOXCOX(TDW_F11)=CLASS(REP ENTRY);

**QUIT**;

**RUN**;

**PROC** **MIXED** DATA=MV;

TITLE1 'MILLVILLE DRY-MATTER';

TITLE2 'FALL 2011';

CLASS REP ENTRY;

MODEL TDW_F11 = ENTRY/OUTP=RESID6;

RANDOM REP;

LSMEANS ENTRY/PDIFF;

ODS OUTPUT DIFFS=EEE LSMEANS=FFF;

ODS EXCLUDE DIFFS;

**RUN**;

%***PDMIX800*** (EEE,FFF,ALPHA=**0.05**,SORT=YES)

%***PDMIX800*** (EEE,FFF,ALPHA=**0.10**,SORT=YES)

QUIT;

**RUN**;

**PROC** **UNIVARIATE** DATA=RESID6 NORMAL PLOT;

VAR RESID;

HISTOGRAM RESID/NORMAL NOFRAME;

PROBPLOT RESID/NORMAL NOFRAME;

**RUN**;

**PROC** **TRANSREG** DATA=MV;

MODEL BOXCOX(TDW_F11)=CLASS(REP ENTRY);

**QUIT**;

**RUN**;

**PROC** **MIXED** DATA=NEPHI;

TITLE1 'NEPHI DRY-MATTER';

TITLE2 'FALL 2011';

CLASS REP ENTRY;

MODEL TDW_F11 = ENTRY/OUTP=RESID7;

RANDOM REP;

LSMEANS ENTRY/PDIFF;

ODS OUTPUT DIFFS=EEE LSMEANS=FFF;

ODS EXCLUDE DIFFS;

**RUN**;

%***PDMIX800*** (EEE,FFF,ALPHA=**0.05**,SORT=YES)

%***PDMIX800*** (EEE,FFF,ALPHA=**0.10**,SORT=YES)

QUIT;

**RUN**;

**PROC** **UNIVARIATE** DATA=RESID7 NORMAL PLOT;

VAR RESID;

HISTOGRAM RESID/NORMAL NOFRAME;

PROBPLOT RESID/NORMAL NOFRAME;

**RUN**;

**PROC** **TRANSREG** DATA=NEPHI;

MODEL BOXCOX(TDW_F11)=CLASS(REP ENTRY);

**QUIT**;

**RUN**;

| LEE SHARP DRY-MATTER |
| --- |
| FALL 2011 |

The Mixed Procedure

| **Model Information** | |
| --- | --- |
| **Data Set** | WORK.SHARP |
| **Dependent Variable** | TDW_F11 |
| **Covariance Structure** | Variance Components |
| **Estimation Method** | REML |
| **Residual Variance Method** | Profile |
| **Fixed Effects SE Method** | Model-Based |
| **Degrees of Freedom Method** | Containment |

| **Class Level Information** | | |
| --- | --- | --- |
| **Class** | **Levels** | **Values** |
| **REP** | 12 | 1 2 3 4 5 6 7 8 9 10 11 12 |
| **ENTRY** | 39 | Acc1156 Acc238 Acc243 Anatone Boardman Discover E49X E58X Goldar P10 P12 P15 P19 P20 P24 P25 P26 P30 P31 P32 P33 P34 P37 P40 P41 P42 P43 P44 P45 P7G3 P7G6 Secar Secar78 T1442 T1561 T1772 T655 Wahluke Whitmar |

| **Dimensions** | |
| --- | --- |
| **Covariance Parameters** | 2 |
| **Columns in X** | 40 |
| **Columns in Z** | 12 |
| **Subjects** | 1 |
| **Max Obs per Subject** | 452 |

| **Number of Observations** | |
| --- | --- |
| **Number of Observations Read** | 468 |
| **Number of Observations Used** | 452 |
| **Number of Observations Not Used** | 16 |

| **Iteration History** | | | |
| --- | --- | --- | --- |
| **Iteration** | **Evaluations** | **-2 Res Log Like** | **Criterion** |
| **0** | 1 | 1443.34942945 |  |
| **1** | 2 | 1348.90530582 | 0.00000003 |
| **2** | 1 | 1348.90529679 | 0.00000000 |

| Convergence criteria met. |
| --- |

| **Covariance Parameter Estimates** | |
| --- | --- |
| **Cov Parm** | **Estimate** |
| **REP** | 0.3897 |
| **Residual** | 1.1354 |

| **Fit Statistics** | |
| --- | --- |
| **-2 Res Log Likelihood** | 1348.9 |
| **AIC (Smaller is Better)** | 1352.9 |
| **AICC (Smaller is Better)** | 1352.9 |
| **BIC (Smaller is Better)** | 1353.9 |

| **Type 3 Tests of Fixed Effects** | | | | |
| --- | --- | --- | --- | --- |
| **Effect** | **Num DF** | **Den DF** | **F Value** | **Pr > F** |
| **ENTRY** | 38 | 402 | 4.60 | <.0001 |

| **Least Squares Means** | | | | | | |
| --- | --- | --- | --- | --- | --- | --- |
| **Effect** | **ENTRY** | **Estimate** | **Standard Error** | **DF** | **t Value** | **Pr > \|t\|** |
| **ENTRY** | **Acc1156** | 3.0922 | 0.3688 | 402 | 8.39 | <.0001 |
| **ENTRY** | **Acc238** | 4.3758 | 0.3565 | 402 | 12.27 | <.0001 |
| **ENTRY** | **Acc243** | 3.8438 | 0.3688 | 402 | 10.42 | <.0001 |
| **ENTRY** | **Anatone** | 4.3743 | 0.3688 | 402 | 11.86 | <.0001 |
| **ENTRY** | **Boardman** | 3.6141 | 0.3565 | 402 | 10.14 | <.0001 |
| **ENTRY** | **Discover** | 3.3069 | 0.3565 | 402 | 9.28 | <.0001 |
| **ENTRY** | **E49X** | 3.4041 | 0.3688 | 402 | 9.23 | <.0001 |
| **ENTRY** | **E58X** | 5.0727 | 0.3565 | 402 | 14.23 | <.0001 |
| **ENTRY** | **Goldar** | 2.4527 | 0.3565 | 402 | 6.88 | <.0001 |
| **ENTRY** | **P10** | 3.9576 | 0.3565 | 402 | 11.10 | <.0001 |
| **ENTRY** | **P12** | 4.0333 | 0.3688 | 402 | 10.94 | <.0001 |
| **ENTRY** | **P15** | 3.8168 | 0.3565 | 402 | 10.71 | <.0001 |
| **ENTRY** | **P19** | 3.4882 | 0.3565 | 402 | 9.78 | <.0001 |
| **ENTRY** | **P20** | 2.3589 | 0.3565 | 402 | 6.62 | <.0001 |
| **ENTRY** | **P24** | 3.1717 | 0.3565 | 402 | 8.90 | <.0001 |
| **ENTRY** | **P25** | 4.4057 | 0.3565 | 402 | 12.36 | <.0001 |
| **ENTRY** | **P26** | 4.1546 | 0.3565 | 402 | 11.65 | <.0001 |
| **ENTRY** | **P30** | 3.9544 | 0.3565 | 402 | 11.09 | <.0001 |
| **ENTRY** | **P31** | 4.0860 | 0.3565 | 402 | 11.46 | <.0001 |
| **ENTRY** | **P32** | 3.4128 | 0.3565 | 402 | 9.57 | <.0001 |
| **ENTRY** | **P33** | 4.8676 | 0.3688 | 402 | 13.20 | <.0001 |
| **ENTRY** | **P34** | 4.8730 | 0.3688 | 402 | 13.21 | <.0001 |
| **ENTRY** | **P37** | 3.5407 | 0.3565 | 402 | 9.93 | <.0001 |
| **ENTRY** | **P40** | 4.2590 | 0.3688 | 402 | 11.55 | <.0001 |
| **ENTRY** | **P41** | 2.6891 | 0.3565 | 402 | 7.54 | <.0001 |
| **ENTRY** | **P42** | 3.6215 | 0.3565 | 402 | 10.16 | <.0001 |
| **ENTRY** | **P43** | 4.1091 | 0.3687 | 402 | 11.15 | <.0001 |
| **ENTRY** | **P44** | 3.5144 | 0.3688 | 402 | 9.53 | <.0001 |
| **ENTRY** | **P45** | 4.2793 | 0.3565 | 402 | 12.00 | <.0001 |
| **ENTRY** | **P7G3** | 3.6096 | 0.3687 | 402 | 9.79 | <.0001 |
| **ENTRY** | **P7G6** | 4.0350 | 0.3688 | 402 | 10.94 | <.0001 |
| **ENTRY** | **Secar** | 3.7215 | 0.3565 | 402 | 10.44 | <.0001 |
| **ENTRY** | **Secar78** | 4.6764 | 0.3565 | 402 | 13.12 | <.0001 |
| **ENTRY** | **T1442** | 3.3360 | 0.3688 | 402 | 9.05 | <.0001 |
| **ENTRY** | **T1561** | 2.2631 | 0.3687 | 402 | 6.14 | <.0001 |
| **ENTRY** | **T1772** | 3.1824 | 0.3565 | 402 | 8.93 | <.0001 |
| **ENTRY** | **T655** | 3.9789 | 0.3687 | 402 | 10.79 | <.0001 |
| **ENTRY** | **Wahluke** | 2.9527 | 0.3565 | 402 | 8.28 | <.0001 |
| **ENTRY** | **Whitmar** | 3.9495 | 0.3688 | 402 | 10.71 | <.0001 |

| LEE SHARP DRY-MATTER |
| --- |
| FALL 2011 |

Effect=ENTRY Method=LSD(P<0.05) Set=1

| **Obs** | **ENTRY** | **Estimate** | **Standard Error** | **Letter Group** |
| --- | --- | --- | --- | --- |
| **1** | E58X | 5.0727 | 0.3565 | A |
| **2** | P34 | 4.8730 | 0.3688 | AB |
| **3** | P33 | 4.8676 | 0.3688 | AB |
| **4** | Secar78 | 4.6764 | 0.3565 | ABC |
| **5** | P25 | 4.4057 | 0.3565 | ABCD |
| **6** | Acc238 | 4.3758 | 0.3565 | ABCDE |
| **7** | Anatone | 4.3743 | 0.3688 | ABCDE |
| **8** | P45 | 4.2793 | 0.3565 | ABCDEF |
| **9** | P40 | 4.2590 | 0.3688 | ABCDEFG |
| **10** | P26 | 4.1546 | 0.3565 | BCDEFGH |
| **11** | P43 | 4.1091 | 0.3687 | BCDEFGH |
| **12** | P31 | 4.0860 | 0.3565 | BCDEFGH |
| **13** | P7G6 | 4.0350 | 0.3688 | BCDEFGHI |
| **14** | P12 | 4.0333 | 0.3688 | BCDEFGHI |
| **15** | T655 | 3.9789 | 0.3687 | BCDEFGHIJ |
| **16** | P10 | 3.9576 | 0.3565 | CDEFGHIJ |
| **17** | P30 | 3.9544 | 0.3565 | CDEFGHIJ |
| **18** | Whitmar | 3.9495 | 0.3688 | CDEFGHIJ |
| **19** | Acc243 | 3.8438 | 0.3688 | CDEFGHIJ |
| **20** | P15 | 3.8168 | 0.3565 | DEFGHIJ |
| **21** | Secar | 3.7215 | 0.3565 | DEFGHIJK |
| **22** | P42 | 3.6215 | 0.3565 | DEFGHIJK |
| **23** | Boardman | 3.6141 | 0.3565 | DEFGHIJK |
| **24** | P7G3 | 3.6096 | 0.3687 | DEFGHIJK |
| **25** | P37 | 3.5407 | 0.3565 | EFGHIJKL |
| **26** | P44 | 3.5144 | 0.3688 | EFGHIJKL |
| **27** | P19 | 3.4882 | 0.3565 | FGHIJKL |
| **28** | P32 | 3.4128 | 0.3565 | GHIJKL |
| **29** | E49X | 3.4041 | 0.3688 | GHIJKL |
| **30** | T1442 | 3.3360 | 0.3688 | HIJKL |
| **31** | Discover | 3.3069 | 0.3565 | HIJKLM |
| **32** | T1772 | 3.1824 | 0.3565 | IJKLMN |
| **33** | P24 | 3.1717 | 0.3565 | IJKLMN |
| **34** | Acc1156 | 3.0922 | 0.3688 | JKLMNO |
| **35** | Wahluke | 2.9527 | 0.3565 | KLMNO |
| **36** | P41 | 2.6891 | 0.3565 | LMNO |
| **37** | Goldar | 2.4527 | 0.3565 | MNO |
| **38** | P20 | 2.3589 | 0.3565 | NO |
| **39** | T1561 | 2.2631 | 0.3687 | O |

| LEE SHARP DRY-MATTER |
| --- |
| FALL 2011 |

Effect=ENTRY Method=LSD(P<0.10) Set=1

| **Obs** | **ENTRY** | **Estimate** | **Standard Error** | **Letter Group** |
| --- | --- | --- | --- | --- |
| **1** | E58X | 5.0727 | 0.3565 | A |
| **2** | P34 | 4.8730 | 0.3688 | AB |
| **3** | P33 | 4.8676 | 0.3688 | AB |
| **4** | Secar78 | 4.6764 | 0.3565 | ABC |
| **5** | P25 | 4.4057 | 0.3565 | ABCD |
| **6** | Acc238 | 4.3758 | 0.3565 | ABCD |
| **7** | Anatone | 4.3743 | 0.3688 | ABCD |
| **8** | P45 | 4.2793 | 0.3565 | BCDE |
| **9** | P40 | 4.2590 | 0.3688 | BCDEF |
| **10** | P26 | 4.1546 | 0.3565 | BCDEFG |
| **11** | P43 | 4.1091 | 0.3687 | CDEFGH |
| **12** | P31 | 4.0860 | 0.3565 | CDEFGH |
| **13** | P7G6 | 4.0350 | 0.3688 | CDEFGHI |
| **14** | P12 | 4.0333 | 0.3688 | CDEFGHI |
| **15** | T655 | 3.9789 | 0.3687 | CDEFGHI |
| **16** | P10 | 3.9576 | 0.3565 | DEFGHI |
| **17** | P30 | 3.9544 | 0.3565 | DEFGHI |
| **18** | Whitmar | 3.9495 | 0.3688 | CDEFGHI |
| **19** | Acc243 | 3.8438 | 0.3688 | DEFGHIJ |
| **20** | P15 | 3.8168 | 0.3565 | DEFGHIJK |
| **21** | Secar | 3.7215 | 0.3565 | DEFGHIJK |
| **22** | P42 | 3.6215 | 0.3565 | EFGHIJKL |
| **23** | Boardman | 3.6141 | 0.3565 | EFGHIJKL |
| **24** | P7G3 | 3.6096 | 0.3687 | EFGHIJKL |
| **25** | P37 | 3.5407 | 0.3565 | FGHIJKL |
| **26** | P44 | 3.5144 | 0.3688 | FGHIJKL |
| **27** | P19 | 3.4882 | 0.3565 | GHIJKL |
| **28** | P32 | 3.4128 | 0.3565 | HIJKL |
| **29** | E49X | 3.4041 | 0.3688 | HIJKLM |
| **30** | T1442 | 3.3360 | 0.3688 | IJKLM |
| **31** | Discover | 3.3069 | 0.3565 | IJKLM |
| **32** | T1772 | 3.1824 | 0.3565 | JKLM |
| **33** | P24 | 3.1717 | 0.3565 | JKLM |
| **34** | Acc1156 | 3.0922 | 0.3688 | KLMN |
| **35** | Wahluke | 2.9527 | 0.3565 | LMNO |
| **36** | P41 | 2.6891 | 0.3565 | MNO |
| **37** | Goldar | 2.4527 | 0.3565 | NO |
| **38** | P20 | 2.3589 | 0.3565 | NO |
| **39** | T1561 | 2.2631 | 0.3687 | O |

| LEE SHARP DRY-MATTER |
| --- |
| FALL 2011 |

The UNIVARIATE Procedure

Variable: Resid (Residual)

| **Moments** | | | |
| --- | --- | --- | --- |
| **N** | 452 | **Sum Weights** | 452 |
| **Mean** | 0 | **Sum Observations** | 0 |
| **Std Deviation** | 1.0070086 | **Variance** | 1.01406632 |
| **Skewness** | -0.0909068 | **Kurtosis** | -0.1563002 |
| **Uncorrected SS** | 457.343912 | **Corrected SS** | 457.343912 |
| **Coeff Variation** | . | **Std Error Mean** | 0.0473657 |

| **Basic Statistical Measures** | | | |
| --- | --- | --- | --- |
| **Location** | | **Variability** | |
| **Mean** | 0.000000 | **Std Deviation** | 1.00701 |
| **Median** | 0.016844 | **Variance** | 1.01407 |
| **Mode** | . | **Range** | 5.96694 |
|  |  | **Interquartile Range** | 1.43212 |

| **Tests for Location: Mu0=0** | | | | |
| --- | --- | --- | --- | --- |
| **Test** | **Statistic** | | **p Value** | |
| **Student's t** | **t** | 0 | **Pr > \|t\|** | 1.0000 |
| **Sign** | **M** | 3 | **Pr >= \|M\|** | 0.8141 |
| **Signed Rank** | **S** | 411 | **Pr >= \|S\|** | 0.8826 |

| **Tests for Normality** | | | | |
| --- | --- | --- | --- | --- |
| **Test** | **Statistic** | | **p Value** | |
| **Shapiro-Wilk** | **W** | 0.997869 | **Pr < W** | 0.8445 |
| **Kolmogorov-Smirnov** | **D** | 0.032338 | **Pr > D** | >0.1500 |
| **Cramer-von Mises** | **W-Sq** | 0.046173 | **Pr > W-Sq** | >0.2500 |
| **Anderson-Darling** | **A-Sq** | 0.281093 | **Pr > A-Sq** | >0.2500 |

| **Quantiles (Definition 5)** | |
| --- | --- |
| **Level** | **Quantile** |
| **100% Max** | 2.9586078 |
| **99%** | 2.2093581 |
| **95%** | 1.6281810 |
| **90%** | 1.3028452 |
| **75% Q3** | 0.7231456 |
| **50% Median** | 0.0168439 |
| **25% Q1** | -0.7089763 |
| **10%** | -1.3510165 |
| **5%** | -1.6321218 |
| **1%** | -2.3390826 |
| **0% Min** | -3.0083328 |

| **Extreme Observations** | | | |
| --- | --- | --- | --- |
| **Lowest** | | **Highest** | |
| **Value** | **Obs** | **Value** | **Obs** |
| -3.00833 | 384 | 2.20936 | 11 |
| -2.60805 | 146 | 2.39041 | 10 |
| -2.60362 | 449 | 2.39436 | 364 |
| -2.34544 | 151 | 2.48821 | 353 |
| -2.33908 | 221 | 2.95861 | 324 |

| **Missing Values** | | | |
| --- | --- | --- | --- |
| **Missing Value** | **Count** | **Percent Of** | |
|  |  | **All Obs** | **Missing Obs** |
| . | 16 | 3.42 | 100.00 |


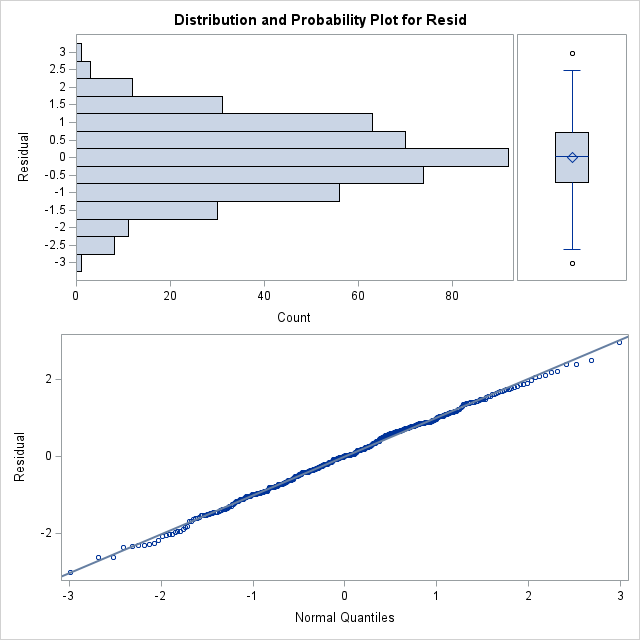


| LEE SHARP DRY-MATTER |
| --- |
| FALL 2011 |

The UNIVARIATE Procedure


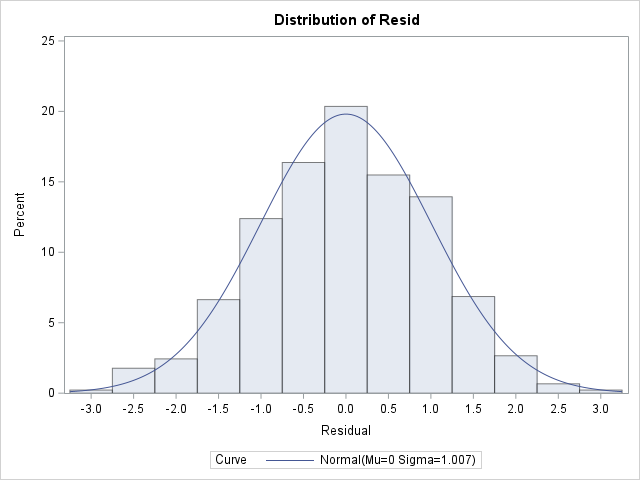


| LEE SHARP DRY-MATTER |
| --- |
| FALL 2011 |

The UNIVARIATE Procedure

Fitted Normal Distribution for Resid (Residual)

| **Parameters for Normal Distribution** | | |
| --- | --- | --- |
| **Parameter** | **Symbol** | **Estimate** |
| **Mean** | Mu | 0 |
| **Std Dev** | Sigma | 1.007009 |

| **Goodness-of-Fit Tests for Normal Distribution** | | | | |
| --- | --- | --- | --- | --- |
| **Test** | **Statistic** | | **p Value** | |
| **Kolmogorov-Smirnov** | **D** | 0.03233845 | **Pr > D** | >0.150 |
| **Cramer-von Mises** | **W-Sq** | 0.04617316 | **Pr > W-Sq** | >0.250 |
| **Anderson-Darling** | **A-Sq** | 0.28109301 | **Pr > A-Sq** | >0.250 |

| **Quantiles for Normal Distribution** | | |
| --- | --- | --- |
| **Percent** | **Quantile** | |
|  | **Observed** | **Estimated** |
| **1.0** | -2.33908 | -2.34265 |
| **5.0** | -1.63212 | -1.65638 |
| **10.0** | -1.35102 | -1.29053 |
| **25.0** | -0.70898 | -0.67922 |
| **50.0** | 0.01684 | 0.00000 |
| **75.0** | 0.72315 | 0.67922 |
| **90.0** | 1.30285 | 1.29053 |
| **95.0** | 1.62818 | 1.65638 |
| **99.0** | 2.20936 | 2.34265 |

| LEE SHARP DRY-MATTER |
| --- |
| FALL 2011 |

The UNIVARIATE Procedure


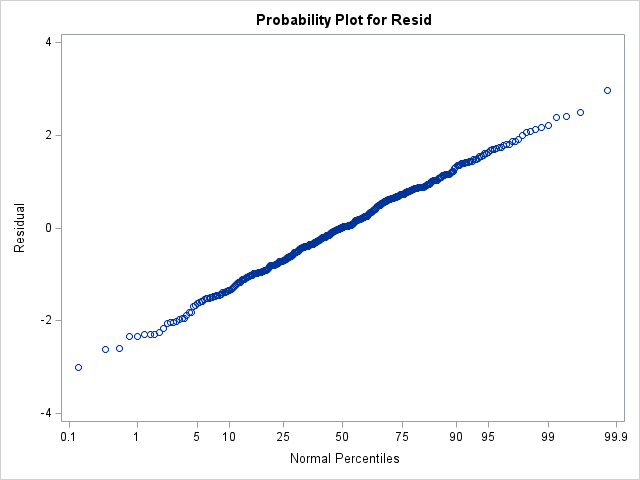


| MILLVILLE DRY-MATTER |
| --- |
| FALL 2011 |

The Mixed Procedure

| **Model Information** | |
| --- | --- |
| **Data Set** | WORK.MV |
| **Dependent Variable** | TDW_F11 |
| **Covariance Structure** | Variance Components |
| **Estimation Method** | REML |
| **Residual Variance Method** | Profile |
| **Fixed Effects SE Method** | Model-Based |
| **Degrees of Freedom Method** | Containment |

| **Class Level Information** | | |
| --- | --- | --- |
| **Class** | **Levels** | **Values** |
| **REP** | 12 | 1 2 3 4 5 6 7 8 9 10 11 12 |
| **ENTRY** | 39 | Acc1156 Acc238 Acc243 Anatone Boardman Discover E49X E58X Goldar P10 P12 P15 P19 P20 P24 P25 P26 P30 P31 P32 P33 P34 P37 P40 P41 P42 P43 P44 P45 P7G3 P7G6 Secar Secar78 T1442 T1561 T1772 T655 Wahluke Whitmar |

| **Dimensions** | |
| --- | --- |
| **Covariance Parameters** | 2 |
| **Columns in X** | 40 |
| **Columns in Z** | 12 |
| **Subjects** | 1 |
| **Max Obs per Subject** | 450 |

| **Number of Observations** | |
| --- | --- |
| **Number of Observations Read** | 468 |
| **Number of Observations Used** | 450 |
| **Number of Observations Not Used** | 18 |

| **Iteration History** | | | |
| --- | --- | --- | --- |
| **Iteration** | **Evaluations** | **-2 Res Log Like** | **Criterion** |
| **0** | 1 | 1153.55171731 |  |
| **1** | 2 | 1009.13213471 | 0.00005525 |
| **2** | 1 | 1009.12481978 | 0.00000026 |
| **3** | 1 | 1009.12478642 | 0.00000000 |

| Convergence criteria met. |
| --- |

| **Covariance Parameter Estimates** | |
| --- | --- |
| **Cov Parm** | **Estimate** |
| **REP** | 0.2905 |
| **Residual** | 0.4976 |

| **Fit Statistics** | |
| --- | --- |
| **-2 Res Log Likelihood** | 1009.1 |
| **AIC (Smaller is Better)** | 1013.1 |
| **AICC (Smaller is Better)** | 1013.2 |
| **BIC (Smaller is Better)** | 1014.1 |

| **Type 3 Tests of Fixed Effects** | | | | |
| --- | --- | --- | --- | --- |
| **Effect** | **Num DF** | **Den DF** | **F Value** | **Pr > F** |
| **ENTRY** | 38 | 400 | 16.92 | <.0001 |

| **Least Squares Means** | | | | | | |
| --- | --- | --- | --- | --- | --- | --- |
| **Effect** | **ENTRY** | **Estimate** | **Standard Error** | **DF** | **t Value** | **Pr > \|t\|** |
| **ENTRY** | **Acc1156** | 5.7406 | 0.2567 | 400 | 22.36 | <.0001 |
| **ENTRY** | **Acc238** | 6.6454 | 0.2724 | 400 | 24.39 | <.0001 |
| **ENTRY** | **Acc243** | 6.7169 | 0.2638 | 400 | 25.47 | <.0001 |
| **ENTRY** | **Anatone** | 6.8184 | 0.2563 | 400 | 26.61 | <.0001 |
| **ENTRY** | **Boardman** | 5.4217 | 0.2563 | 400 | 21.16 | <.0001 |
| **ENTRY** | **Discover** | 6.2103 | 0.2563 | 400 | 24.23 | <.0001 |
| **ENTRY** | **E49X** | 5.9344 | 0.2638 | 400 | 22.50 | <.0001 |
| **ENTRY** | **E58X** | 6.0560 | 0.2724 | 400 | 22.23 | <.0001 |
| **ENTRY** | **Goldar** | 6.4017 | 0.2563 | 400 | 24.98 | <.0001 |
| **ENTRY** | **P10** | 6.8476 | 0.2724 | 400 | 25.14 | <.0001 |
| **ENTRY** | **P12** | 6.5386 | 0.2638 | 400 | 24.79 | <.0001 |
| **ENTRY** | **P15** | 6.4832 | 0.2638 | 400 | 24.58 | <.0001 |
| **ENTRY** | **P19** | 7.0056 | 0.2637 | 400 | 26.56 | <.0001 |
| **ENTRY** | **P20** | 5.8156 | 0.2563 | 400 | 22.69 | <.0001 |
| **ENTRY** | **P24** | 6.1730 | 0.2563 | 400 | 24.09 | <.0001 |
| **ENTRY** | **P25** | 7.1422 | 0.2563 | 400 | 27.87 | <.0001 |
| **ENTRY** | **P26** | 7.3791 | 0.2563 | 400 | 28.79 | <.0001 |
| **ENTRY** | **P30** | 4.6826 | 0.2563 | 400 | 18.27 | <.0001 |
| **ENTRY** | **P31** | 7.2262 | 0.2563 | 400 | 28.20 | <.0001 |
| **ENTRY** | **P32** | 6.9434 | 0.2563 | 400 | 27.09 | <.0001 |
| **ENTRY** | **P33** | 7.1664 | 0.2563 | 400 | 27.96 | <.0001 |
| **ENTRY** | **P34** | 6.5595 | 0.2637 | 400 | 24.87 | <.0001 |
| **ENTRY** | **P37** | 4.7350 | 0.2563 | 400 | 18.48 | <.0001 |
| **ENTRY** | **P40** | 7.0722 | 0.2638 | 400 | 26.81 | <.0001 |
| **ENTRY** | **P41** | 6.1945 | 0.2563 | 400 | 24.17 | <.0001 |
| **ENTRY** | **P42** | 5.9067 | 0.2563 | 400 | 23.05 | <.0001 |
| **ENTRY** | **P43** | 6.9787 | 0.2563 | 400 | 27.23 | <.0001 |
| **ENTRY** | **P44** | 7.0333 | 0.2563 | 400 | 27.45 | <.0001 |
| **ENTRY** | **P45** | 6.9087 | 0.2563 | 400 | 26.96 | <.0001 |
| **ENTRY** | **P7G3** | 6.6018 | 0.2563 | 400 | 25.76 | <.0001 |
| **ENTRY** | **P7G6** | 7.0356 | 0.2563 | 400 | 27.45 | <.0001 |
| **ENTRY** | **Secar** | 4.5424 | 0.2563 | 400 | 17.73 | <.0001 |
| **ENTRY** | **Secar78** | 5.7501 | 0.2563 | 400 | 22.44 | <.0001 |
| **ENTRY** | **T1442** | 4.5087 | 0.2563 | 400 | 17.59 | <.0001 |
| **ENTRY** | **T1561** | 4.5978 | 0.2724 | 400 | 16.88 | <.0001 |
| **ENTRY** | **T1772** | 4.7854 | 0.2638 | 400 | 18.14 | <.0001 |
| **ENTRY** | **T655** | 6.8057 | 0.2724 | 400 | 24.98 | <.0001 |
| **ENTRY** | **Wahluke** | 5.4590 | 0.2563 | 400 | 21.30 | <.0001 |
| **ENTRY** | **Whitmar** | 5.7571 | 0.2563 | 400 | 22.47 | <.0001 |

| MILLVILLE DRY-MATTER |
| --- |
| FALL 2011 |

Effect=ENTRY Method=LSD(P<0.05) Set=1

| **Obs** | **ENTRY** | **Estimate** | **Standard Error** | **Letter Group** |
| --- | --- | --- | --- | --- |
| **1** | P26 | 7.3791 | 0.2563 | A |
| **2** | P31 | 7.2262 | 0.2563 | AB |
| **3** | P33 | 7.1664 | 0.2563 | ABC |
| **4** | P25 | 7.1422 | 0.2563 | ABC |
| **5** | P40 | 7.0722 | 0.2638 | ABCD |
| **6** | P7G6 | 7.0356 | 0.2563 | ABCD |
| **7** | P44 | 7.0333 | 0.2563 | ABCD |
| **8** | P19 | 7.0056 | 0.2637 | ABCD |
| **9** | P43 | 6.9787 | 0.2563 | ABCD |
| **10** | P32 | 6.9434 | 0.2563 | ABCDE |
| **11** | P45 | 6.9087 | 0.2563 | ABCDE |
| **12** | P10 | 6.8476 | 0.2724 | ABCDE |
| **13** | Anatone | 6.8184 | 0.2563 | ABCDE |
| **14** | T655 | 6.8057 | 0.2724 | ABCDE |
| **15** | Acc243 | 6.7169 | 0.2638 | BCDEF |
| **16** | Acc238 | 6.6454 | 0.2724 | BCDEFG |
| **17** | P7G3 | 6.6018 | 0.2563 | CDEFG |
| **18** | P34 | 6.5595 | 0.2637 | DEFG |
| **19** | P12 | 6.5386 | 0.2638 | DEFG |
| **20** | P15 | 6.4832 | 0.2638 | DEFGH |
| **21** | Goldar | 6.4017 | 0.2563 | EFGH |
| **22** | Discover | 6.2103 | 0.2563 | FGHI |
| **23** | P41 | 6.1945 | 0.2563 | FGHI |
| **24** | P24 | 6.1730 | 0.2563 | FGHI |
| **25** | E58X | 6.0560 | 0.2724 | GHI |
| **26** | E49X | 5.9344 | 0.2638 | HIJ |
| **27** | P42 | 5.9067 | 0.2563 | HIJ |
| **28** | P20 | 5.8156 | 0.2563 | IJ |
| **29** | Whitmar | 5.7571 | 0.2563 | IJ |
| **30** | Secar78 | 5.7501 | 0.2563 | IJ |
| **31** | Acc1156 | 5.7406 | 0.2567 | IJ |
| **32** | Wahluke | 5.4590 | 0.2563 | J |
| **33** | Boardman | 5.4217 | 0.2563 | J |
| **34** | T1772 | 4.7854 | 0.2638 | K |
| **35** | P37 | 4.7350 | 0.2563 | K |
| **36** | P30 | 4.6826 | 0.2563 | K |
| **37** | T1561 | 4.5978 | 0.2724 | K |
| **38** | Secar | 4.5424 | 0.2563 | K |
| **39** | T1442 | 4.5087 | 0.2563 | K |

| MILLVILLE DRY-MATTER |
| --- |
| FALL 2011 |

Effect=ENTRY Method=LSD(P<0.10) Set=1

| **Obs** | **ENTRY** | **Estimate** | **Standard Error** | **Letter Group** |
| --- | --- | --- | --- | --- |
| **1** | P26 | 7.3791 | 0.2563 | A |
| **2** | P31 | 7.2262 | 0.2563 | AB |
| **3** | P33 | 7.1664 | 0.2563 | ABC |
| **4** | P25 | 7.1422 | 0.2563 | ABCD |
| **5** | P40 | 7.0722 | 0.2638 | ABCDE |
| **6** | P7G6 | 7.0356 | 0.2563 | ABCDEF |
| **7** | P44 | 7.0333 | 0.2563 | ABCDEF |
| **8** | P19 | 7.0056 | 0.2637 | ABCDEFG |
| **9** | P43 | 6.9787 | 0.2563 | ABCDEFG |
| **10** | P32 | 6.9434 | 0.2563 | ABCDEFGH |
| **11** | P45 | 6.9087 | 0.2563 | ABCDEFGH |
| **12** | P10 | 6.8476 | 0.2724 | BCDEFGHI |
| **13** | Anatone | 6.8184 | 0.2563 | BCDEFGHI |
| **14** | T655 | 6.8057 | 0.2724 | BCDEFGHI |
| **15** | Acc243 | 6.7169 | 0.2638 | CDEFGHI |
| **16** | Acc238 | 6.6454 | 0.2724 | DEFGHIJ |
| **17** | P7G3 | 6.6018 | 0.2563 | EFGHIJ |
| **18** | P34 | 6.5595 | 0.2637 | FGHIJK |
| **19** | P12 | 6.5386 | 0.2638 | GHIJK |
| **20** | P15 | 6.4832 | 0.2638 | HIJK |
| **21** | Goldar | 6.4017 | 0.2563 | IJKL |
| **22** | Discover | 6.2103 | 0.2563 | JKLM |
| **23** | P41 | 6.1945 | 0.2563 | JKLM |
| **24** | P24 | 6.1730 | 0.2563 | JKLM |
| **25** | E58X | 6.0560 | 0.2724 | KLM |
| **26** | E49X | 5.9344 | 0.2638 | LMN |
| **27** | P42 | 5.9067 | 0.2563 | MN |
| **28** | P20 | 5.8156 | 0.2563 | MNO |
| **29** | Whitmar | 5.7571 | 0.2563 | MNO |
| **30** | Secar78 | 5.7501 | 0.2563 | MNO |
| **31** | Acc1156 | 5.7406 | 0.2567 | MNO |
| **32** | Wahluke | 5.4590 | 0.2563 | NO |
| **33** | Boardman | 5.4217 | 0.2563 | O |
| **34** | T1772 | 4.7854 | 0.2638 | P |
| **35** | P37 | 4.7350 | 0.2563 | P |
| **36** | P30 | 4.6826 | 0.2563 | P |
| **37** | T1561 | 4.5978 | 0.2724 | P |
| **38** | Secar | 4.5424 | 0.2563 | P |
| **39** | T1442 | 4.5087 | 0.2563 | P |

| MILLVILLE DRY-MATTER |
| --- |
| FALL 2011 |

The UNIVARIATE Procedure

Variable: Resid (Residual)

| **Moments** | | | |
| --- | --- | --- | --- |
| **N** | 450 | **Sum Weights** | 450 |
| **Mean** | 0 | **Sum Observations** | 0 |
| **Std Deviation** | 0.66618792 | **Variance** | 0.44380634 |
| **Skewness** | 0.11692726 | **Kurtosis** | 0.20473859 |
| **Uncorrected SS** | 199.269047 | **Corrected SS** | 199.269047 |
| **Coeff Variation** | . | **Std Error Mean** | 0.0314044 |

| **Basic Statistical Measures** | | | |
| --- | --- | --- | --- |
| **Location** | | **Variability** | |
| **Mean** | 0.00000 | **Std Deviation** | 0.66619 |
| **Median** | -0.00226 | **Variance** | 0.44381 |
| **Mode** | . | **Range** | 3.83582 |
|  |  | **Interquartile Range** | 0.86908 |

| **Tests for Location: Mu0=0** | | | | |
| --- | --- | --- | --- | --- |
| **Test** | **Statistic** | | **p Value** | |
| **Student's t** | **t** | 0 | **Pr > \|t\|** | 1.0000 |
| **Sign** | **M** | -2 | **Pr >= \|M\|** | 0.8876 |
| **Signed Rank** | **S** | -652.5 | **Pr >= \|S\|** | 0.8134 |

| **Tests for Normality** | | | | |
| --- | --- | --- | --- | --- |
| **Test** | **Statistic** | | **p Value** | |
| **Shapiro-Wilk** | **W** | 0.996172 | **Pr < W** | 0.3527 |
| **Kolmogorov-Smirnov** | **D** | 0.028615 | **Pr > D** | >0.1500 |
| **Cramer-von Mises** | **W-Sq** | 0.051051 | **Pr > W-Sq** | >0.2500 |
| **Anderson-Darling** | **A-Sq** | 0.346619 | **Pr > A-Sq** | >0.2500 |

| **Quantiles (Definition 5)** | |
| --- | --- |
| **Level** | **Quantile** |
| **100% Max** | 1.98363050 |
| **99%** | 1.84782448 |
| **95%** | 1.11048107 |
| **90%** | 0.87754171 |
| **75% Q3** | 0.42367202 |
| **50% Median** | -0.00225526 |
| **25% Q1** | -0.44540575 |
| **10%** | -0.82631578 |
| **5%** | -1.13198220 |
| **1%** | -1.66807464 |
| **0% Min** | -1.85219267 |

| **Extreme Observations** | | | |
| --- | --- | --- | --- |
| **Lowest** | | **Highest** | |
| **Value** | **Obs** | **Value** | **Obs** |
| -1.85219 | 297 | 1.84782 | 99 |
| -1.81766 | 197 | 1.85417 | 100 |
| -1.78274 | 332 | 1.85798 | 464 |
| -1.72757 | 153 | 1.85882 | 27 |
| -1.66807 | 4 | 1.98363 | 13 |

| **Missing Values** | | | |
| --- | --- | --- | --- |
| **Missing Value** | **Count** | **Percent Of** | |
|  |  | **All Obs** | **Missing Obs** |
| . | 18 | 3.85 | 100.00 |


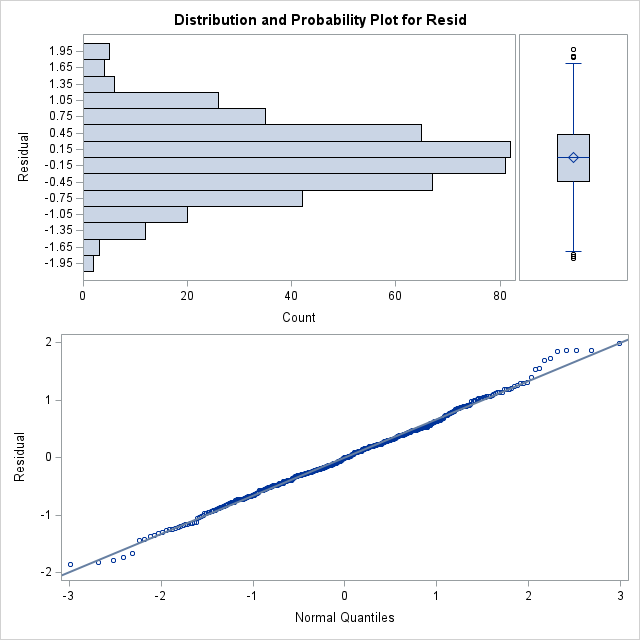


| MILLVILLE DRY-MATTER |
| --- |
| FALL 2011 |

The UNIVARIATE Procedure


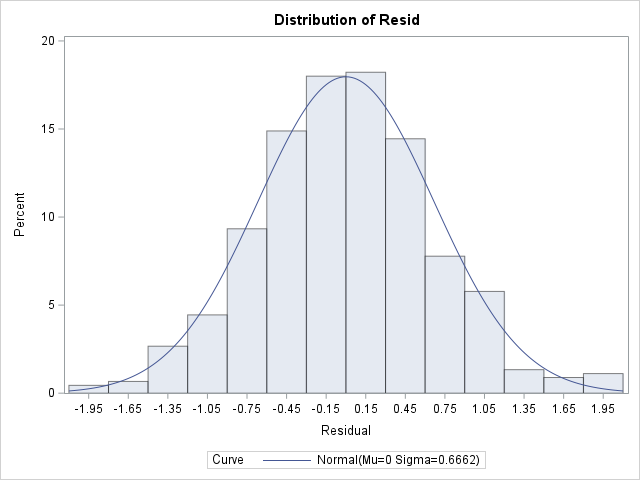


| MILLVILLE DRY-MATTER |
| --- |
| FALL 2011 |

The UNIVARIATE Procedure

Fitted Normal Distribution for Resid (Residual)

| **Parameters for Normal Distribution** | | |
| --- | --- | --- |
| **Parameter** | **Symbol** | **Estimate** |
| **Mean** | Mu | 0 |
| **Std Dev** | Sigma | 0.666188 |

| **Goodness-of-Fit Tests for Normal Distribution** | | | | |
| --- | --- | --- | --- | --- |
| **Test** | **Statistic** | | **p Value** | |
| **Kolmogorov-Smirnov** | **D** | 0.02861521 | **Pr > D** | >0.150 |
| **Cramer-von Mises** | **W-Sq** | 0.05105142 | **Pr > W-Sq** | >0.250 |
| **Anderson-Darling** | **A-Sq** | 0.34661904 | **Pr > A-Sq** | >0.250 |

| **Quantiles for Normal Distribution** | | |
| --- | --- | --- |
| **Percent** | **Quantile** | |
|  | **Observed** | **Estimated** |
| **1.0** | -1.66807 | -1.54978 |
| **5.0** | -1.13198 | -1.09578 |
| **10.0** | -0.82632 | -0.85375 |
| **25.0** | -0.44541 | -0.44934 |
| **50.0** | -0.00226 | 0.00000 |
| **75.0** | 0.42367 | 0.44934 |
| **90.0** | 0.87754 | 0.85375 |
| **95.0** | 1.11048 | 1.09578 |
| **99.0** | 1.84782 | 1.54978 |

| MILLVILLE DRY-MATTER |
| --- |
| FALL 2011 |

The UNIVARIATE Procedure


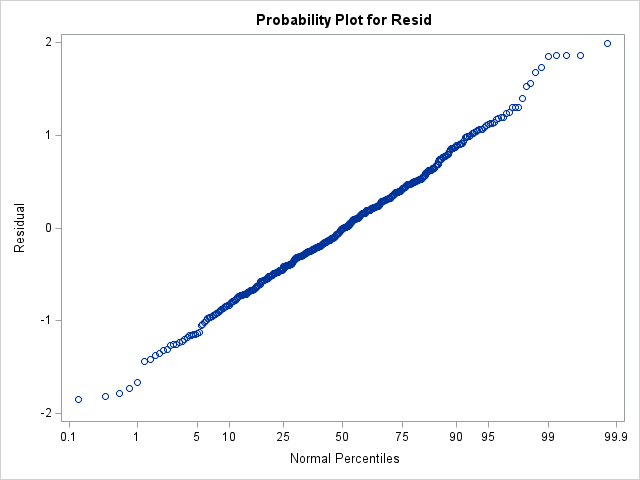


| MILLVILLE DRY-MATTER |
| --- |
| FALL 2011 |

The TRANSREG Procedure


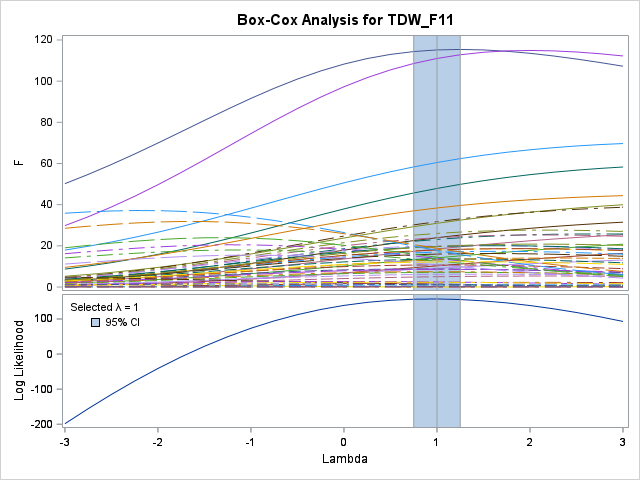


| MILLVILLE DRY-MATTER |
| --- |
| FALL 2011 |

The TRANSREG Procedure

| **TRANSREG Univariate Algorithm Iteration History for BoxCox(TDW_F11)** | | | | | |
| --- | --- | --- | --- | --- | --- |
| **Iteration Number** | **Average Change** | **Maximum Change** | **R-Square** | **Criterion Change** | **Note** |
| **1** | 0.00000 | 0.00000 | 0.68733 |  | Converged |

| Algorithm converged. |
| --- |

| NEPHI DRY-MATTER |
| --- |
| FALL 2011 |

The Mixed Procedure

| **Model Information** | |
| --- | --- |
| **Data Set** | WORK.NEPHI |
| **Dependent Variable** | TDW_F11 |
| **Covariance Structure** | Variance Components |
| **Estimation Method** | REML |
| **Residual Variance Method** | Profile |
| **Fixed Effects SE Method** | Model-Based |
| **Degrees of Freedom Method** | Containment |

| **Class Level Information** | | |
| --- | --- | --- |
| **Class** | **Levels** | **Values** |
| **REP** | 12 | 1 2 3 4 5 6 7 8 9 10 11 12 |
| **ENTRY** | 39 | Acc1156 Acc238 Acc243 Anatone Boardman Discover E49X E58X Goldar P10 P12 P15 P19 P20 P24 P25 P26 P30 P31 P32 P33 P34 P37 P40 P41 P42 P43 P44 P45 P7G3 P7G6 Secar Secar78 T1442 T1561 T1772 T655 Wahluke Whitmar |

| **Dimensions** | |
| --- | --- |
| **Covariance Parameters** | 2 |
| **Columns in X** | 40 |
| **Columns in Z** | 12 |
| **Subjects** | 1 |
| **Max Obs per Subject** | 454 |

| **Number of Observations** | |
| --- | --- |
| **Number of Observations Read** | 468 |
| **Number of Observations Used** | 454 |
| **Number of Observations Not Used** | 14 |

| **Iteration History** | | | |
| --- | --- | --- | --- |
| **Iteration** | **Evaluations** | **-2 Res Log Like** | **Criterion** |
| **0** | 1 | 1381.90489132 |  |
| **1** | 2 | 1260.43501380 | 0.00003772 |
| **2** | 1 | 1260.42516155 | 0.00000024 |
| **3** | 1 | 1260.42510238 | 0.00000000 |

| Convergence criteria met. |
| --- |

| **Covariance Parameter Estimates** | |
| --- | --- |
| **Cov Parm** | **Estimate** |
| **REP** | 0.4316 |
| **Residual** | 0.8963 |

| **Fit Statistics** | |
| --- | --- |
| **-2 Res Log Likelihood** | 1260.4 |
| **AIC (Smaller is Better)** | 1264.4 |
| **AICC (Smaller is Better)** | 1264.5 |
| **BIC (Smaller is Better)** | 1265.4 |

| **Type 3 Tests of Fixed Effects** | | | | |
| --- | --- | --- | --- | --- |
| **Effect** | **Num DF** | **Den DF** | **F Value** | **Pr > F** |
| **ENTRY** | 38 | 404 | 16.61 | <.0001 |

| **Least Squares Means** | | | | | | |
| --- | --- | --- | --- | --- | --- | --- |
| **Effect** | **ENTRY** | **Estimate** | **Standard Error** | **DF** | **t Value** | **Pr > \|t\|** |
| **ENTRY** | **Acc1156** | 5.0792 | 0.3550 | 404 | 14.31 | <.0001 |
| **ENTRY** | **Acc238** | 6.1566 | 0.3327 | 404 | 18.51 | <.0001 |
| **ENTRY** | **Acc243** | 6.6542 | 0.3327 | 404 | 20.00 | <.0001 |
| **ENTRY** | **Anatone** | 6.6910 | 0.3430 | 404 | 19.51 | <.0001 |
| **ENTRY** | **Boardman** | 5.1331 | 0.3430 | 404 | 14.97 | <.0001 |
| **ENTRY** | **Discover** | 6.7098 | 0.3327 | 404 | 20.17 | <.0001 |
| **ENTRY** | **E49X** | 7.0068 | 0.3327 | 404 | 21.06 | <.0001 |
| **ENTRY** | **E58X** | 8.3568 | 0.3327 | 404 | 25.12 | <.0001 |
| **ENTRY** | **Goldar** | 5.8810 | 0.3430 | 404 | 17.15 | <.0001 |
| **ENTRY** | **P10** | 7.0490 | 0.3430 | 404 | 20.55 | <.0001 |
| **ENTRY** | **P12** | 6.1387 | 0.3691 | 404 | 16.63 | <.0001 |
| **ENTRY** | **P15** | 7.0838 | 0.3550 | 404 | 19.96 | <.0001 |
| **ENTRY** | **P19** | 8.1713 | 0.3430 | 404 | 23.82 | <.0001 |
| **ENTRY** | **P20** | 6.8292 | 0.3327 | 404 | 20.53 | <.0001 |
| **ENTRY** | **P24** | 6.0928 | 0.3327 | 404 | 18.32 | <.0001 |
| **ENTRY** | **P25** | 6.2736 | 0.3327 | 404 | 18.86 | <.0001 |
| **ENTRY** | **P26** | 7.4649 | 0.3327 | 404 | 22.44 | <.0001 |
| **ENTRY** | **P30** | 5.1167 | 0.3327 | 404 | 15.38 | <.0001 |
| **ENTRY** | **P31** | 7.4606 | 0.3327 | 404 | 22.43 | <.0001 |
| **ENTRY** | **P32** | 7.0463 | 0.3327 | 404 | 21.18 | <.0001 |
| **ENTRY** | **P33** | 7.2554 | 0.3327 | 404 | 21.81 | <.0001 |
| **ENTRY** | **P34** | 6.5957 | 0.3327 | 404 | 19.83 | <.0001 |
| **ENTRY** | **P37** | 4.3249 | 0.3430 | 404 | 12.61 | <.0001 |
| **ENTRY** | **P40** | 7.4385 | 0.3327 | 404 | 22.36 | <.0001 |
| **ENTRY** | **P41** | 6.6772 | 0.3327 | 404 | 20.07 | <.0001 |
| **ENTRY** | **P42** | 5.9615 | 0.3327 | 404 | 17.92 | <.0001 |
| **ENTRY** | **P43** | 7.1043 | 0.3327 | 404 | 21.36 | <.0001 |
| **ENTRY** | **P44** | 6.5327 | 0.3327 | 404 | 19.64 | <.0001 |
| **ENTRY** | **P45** | 6.9501 | 0.3327 | 404 | 20.89 | <.0001 |
| **ENTRY** | **P7G3** | 7.6191 | 0.3430 | 404 | 22.21 | <.0001 |
| **ENTRY** | **P7G6** | 7.5095 | 0.3327 | 404 | 22.57 | <.0001 |
| **ENTRY** | **Secar** | 5.6460 | 0.3327 | 404 | 16.97 | <.0001 |
| **ENTRY** | **Secar78** | 7.0024 | 0.3327 | 404 | 21.05 | <.0001 |
| **ENTRY** | **T1442** | 3.9220 | 0.3327 | 404 | 11.79 | <.0001 |
| **ENTRY** | **T1561** | 3.3022 | 0.3327 | 404 | 9.93 | <.0001 |
| **ENTRY** | **T1772** | 4.3826 | 0.3327 | 404 | 13.17 | <.0001 |
| **ENTRY** | **T655** | 6.8603 | 0.3327 | 404 | 20.62 | <.0001 |
| **ENTRY** | **Wahluke** | 5.8079 | 0.3327 | 404 | 17.46 | <.0001 |
| **ENTRY** | **Whitmar** | 5.7708 | 0.3327 | 404 | 17.35 | <.0001 |

| NEPHI DRY-MATTER |
| --- |
| FALL 2011 |

Effect=ENTRY Method=LSD(P<0.05) Set=1

| **Obs** | **ENTRY** | **Estimate** | **Standard Error** | **Letter Group** |
| --- | --- | --- | --- | --- |
| **1** | E58X | 8.3568 | 0.3327 | A |
| **2** | P19 | 8.1713 | 0.3430 | AB |
| **3** | P7G3 | 7.6191 | 0.3430 | ABC |
| **4** | P7G6 | 7.5095 | 0.3327 | BCD |
| **5** | P26 | 7.4649 | 0.3327 | BCDE |
| **6** | P31 | 7.4606 | 0.3327 | BCDE |
| **7** | P40 | 7.4385 | 0.3327 | BCDE |
| **8** | P33 | 7.2554 | 0.3327 | CDEF |
| **9** | P43 | 7.1043 | 0.3327 | CDEF |
| **10** | P15 | 7.0838 | 0.3550 | CDEF |
| **11** | P10 | 7.0490 | 0.3430 | CDEFG |
| **12** | P32 | 7.0463 | 0.3327 | CDEF |
| **13** | E49X | 7.0068 | 0.3327 | CDEFG |
| **14** | Secar78 | 7.0024 | 0.3327 | CDEFG |
| **15** | P45 | 6.9501 | 0.3327 | CDEFGH |
| **16** | T655 | 6.8603 | 0.3327 | CDEFGHI |
| **17** | P20 | 6.8292 | 0.3327 | DEFGHIJ |
| **18** | Discover | 6.7098 | 0.3327 | EFGHIJK |
| **19** | Anatone | 6.6910 | 0.3430 | EFGHIJK |
| **20** | P41 | 6.6772 | 0.3327 | FGHIJK |
| **21** | Acc243 | 6.6542 | 0.3327 | FGHIJKL |
| **22** | P34 | 6.5957 | 0.3327 | FGHIJKL |
| **23** | P44 | 6.5327 | 0.3327 | FGHIJKLM |
| **24** | P25 | 6.2736 | 0.3327 | GHIJKLMN |
| **25** | Acc238 | 6.1566 | 0.3327 | IJKLMN |
| **26** | P12 | 6.1387 | 0.3691 | HIJKLMN |
| **27** | P24 | 6.0928 | 0.3327 | JKLMN |
| **28** | P42 | 5.9615 | 0.3327 | KLMN |
| **29** | Goldar | 5.8810 | 0.3430 | LMNO |
| **30** | Wahluke | 5.8079 | 0.3327 | MNO |
| **31** | Whitmar | 5.7708 | 0.3327 | NO |
| **32** | Secar | 5.6460 | 0.3327 | NO |
| **33** | Boardman | 5.1331 | 0.3430 | OP |
| **34** | P30 | 5.1167 | 0.3327 | OP |
| **35** | Acc1156 | 5.0792 | 0.3550 | OPQ |
| **36** | T1772 | 4.3826 | 0.3327 | PQR |
| **37** | P37 | 4.3249 | 0.3430 | QR |
| **38** | T1442 | 3.9220 | 0.3327 | RS |
| **39** | T1561 | 3.3022 | 0.3327 | S |

| NEPHI DRY-MATTER |
| --- |
| FALL 2011 |

Effect=ENTRY Method=LSD(P<0.10) Set=1

| **Obs** | **ENTRY** | **Estimate** | **Standard Error** | **Letter Group** |
| --- | --- | --- | --- | --- |
| **1** | E58X | 8.3568 | 0.3327 | A |
| **2** | P19 | 8.1713 | 0.3430 | AB |
| **3** | P7G3 | 7.6191 | 0.3430 | BC |
| **4** | P7G6 | 7.5095 | 0.3327 | CD |
| **5** | P26 | 7.4649 | 0.3327 | CDE |
| **6** | P31 | 7.4606 | 0.3327 | CDE |
| **7** | P40 | 7.4385 | 0.3327 | CDE |
| **8** | P33 | 7.2554 | 0.3327 | CDEF |
| **9** | P43 | 7.1043 | 0.3327 | CDEFG |
| **10** | P15 | 7.0838 | 0.3550 | CDEFG |
| **11** | P10 | 7.0490 | 0.3430 | CDEFG |
| **12** | P32 | 7.0463 | 0.3327 | CDEFG |
| **13** | E49X | 7.0068 | 0.3327 | CDEFG |
| **14** | Secar78 | 7.0024 | 0.3327 | CDEFG |
| **15** | P45 | 6.9501 | 0.3327 | DEFG |
| **16** | T655 | 6.8603 | 0.3327 | EFGH |
| **17** | P20 | 6.8292 | 0.3327 | EFGH |
| **18** | Discover | 6.7098 | 0.3327 | FGHI |
| **19** | Anatone | 6.6910 | 0.3430 | FGHI |
| **20** | P41 | 6.6772 | 0.3327 | FGHI |
| **21** | Acc243 | 6.6542 | 0.3327 | FGHI |
| **22** | P34 | 6.5957 | 0.3327 | GHIJ |
| **23** | P44 | 6.5327 | 0.3327 | GHIJK |
| **24** | P25 | 6.2736 | 0.3327 | HIJKL |
| **25** | Acc238 | 6.1566 | 0.3327 | IJKL |
| **26** | P12 | 6.1387 | 0.3691 | IJKL |
| **27** | P24 | 6.0928 | 0.3327 | IJKL |
| **28** | P42 | 5.9615 | 0.3327 | JKL |
| **29** | Goldar | 5.8810 | 0.3430 | KL |
| **30** | Wahluke | 5.8079 | 0.3327 | L |
| **31** | Whitmar | 5.7708 | 0.3327 | LM |
| **32** | Secar | 5.6460 | 0.3327 | LMN |
| **33** | Boardman | 5.1331 | 0.3430 | MN |
| **34** | P30 | 5.1167 | 0.3327 | N |
| **35** | Acc1156 | 5.0792 | 0.3550 | N |
| **36** | T1772 | 4.3826 | 0.3327 | O |
| **37** | P37 | 4.3249 | 0.3430 | O |
| **38** | T1442 | 3.9220 | 0.3327 | OP |
| **39** | T1561 | 3.3022 | 0.3327 | P |

| NEPHI DRY-MATTER |
| --- |
| FALL 2011 |

The UNIVARIATE Procedure

Variable: Resid (Residual)

| **Moments** | | | |
| --- | --- | --- | --- |
| **N** | 454 | **Sum Weights** | 454 |
| **Mean** | 0 | **Sum Observations** | 0 |
| **Std Deviation** | 0.89471873 | **Variance** | 0.80052161 |
| **Skewness** | -0.2302582 | **Kurtosis** | 0.02633328 |
| **Uncorrected SS** | 362.636289 | **Corrected SS** | 362.636289 |
| **Coeff Variation** | . | **Std Error Mean** | 0.04199123 |

| **Basic Statistical Measures** | | | |
| --- | --- | --- | --- |
| **Location** | | **Variability** | |
| **Mean** | 0.000000 | **Std Deviation** | 0.89472 |
| **Median** | 0.055268 | **Variance** | 0.80052 |
| **Mode** | . | **Range** | 5.23927 |
|  |  | **Interquartile Range** | 1.15911 |

| **Tests for Location: Mu0=0** | | | | |
| --- | --- | --- | --- | --- |
| **Test** | **Statistic** | | **p Value** | |
| **Student's t** | **t** | 0 | **Pr > \|t\|** | 1.0000 |
| **Sign** | **M** | 10 | **Pr >= \|M\|** | 0.3726 |
| **Signed Rank** | **S** | 1368.5 | **Pr >= \|S\|** | 0.6252 |

| **Tests for Normality** | | | | |
| --- | --- | --- | --- | --- |
| **Test** | **Statistic** | | **p Value** | |
| **Shapiro-Wilk** | **W** | 0.995589 | **Pr < W** | 0.2307 |
| **Kolmogorov-Smirnov** | **D** | 0.028738 | **Pr > D** | >0.1500 |
| **Cramer-von Mises** | **W-Sq** | 0.084745 | **Pr > W-Sq** | 0.1862 |
| **Anderson-Darling** | **A-Sq** | 0.533956 | **Pr > A-Sq** | 0.1787 |

| **Quantiles (Definition 5)** | |
| --- | --- |
| **Level** | **Quantile** |
| **100% Max** | 2.4032049 |
| **99%** | 1.9623414 |
| **95%** | 1.3503506 |
| **90%** | 1.1055152 |
| **75% Q3** | 0.5928770 |
| **50% Median** | 0.0552677 |
| **25% Q1** | -0.5662316 |
| **10%** | -1.1918787 |
| **5%** | -1.5692832 |
| **1%** | -2.2054255 |
| **0% Min** | -2.8360689 |

| **Extreme Observations** | | | |
| --- | --- | --- | --- |
| **Lowest** | | **Highest** | |
| **Value** | **Obs** | **Value** | **Obs** |
| -2.83607 | 182 | 1.96234 | 109 |
| -2.49974 | 160 | 2.12694 | 51 |
| -2.31268 | 112 | 2.24887 | 417 |
| -2.26382 | 3 | 2.26056 | 23 |
| -2.20543 | 258 | 2.40320 | 31 |

| **Missing Values** | | | |
| --- | --- | --- | --- |
| **Missing Value** | **Count** | **Percent Of** | |
|  |  | **All Obs** | **Missing Obs** |
| . | 14 | 2.99 | 100.00 |


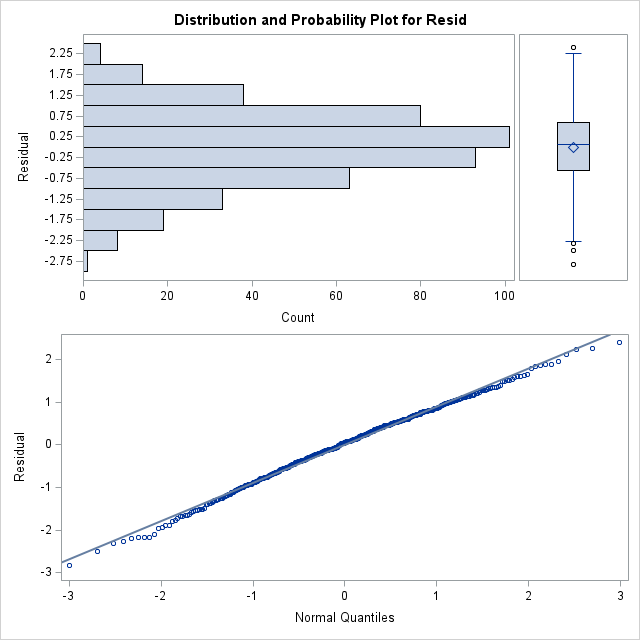


| NEPHI DRY-MATTER |
| --- |
| FALL 2011 |

The UNIVARIATE Procedure


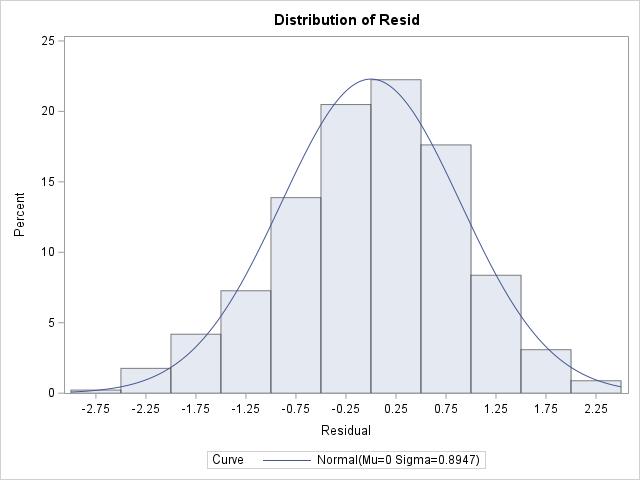


| NEPHI DRY-MATTER |
| --- |
| FALL 2011 |

The UNIVARIATE Procedure

Fitted Normal Distribution for Resid (Residual)

| **Parameters for Normal Distribution** | | |
| --- | --- | --- |
| **Parameter** | **Symbol** | **Estimate** |
| **Mean** | Mu | 0 |
| **Std Dev** | Sigma | 0.894719 |

| **Goodness-of-Fit Tests for Normal Distribution** | | | | |
| --- | --- | --- | --- | --- |
| **Test** | **Statistic** | | **p Value** | |
| **Kolmogorov-Smirnov** | **D** | 0.02873781 | **Pr > D** | >0.150 |
| **Cramer-von Mises** | **W-Sq** | 0.08474461 | **Pr > W-Sq** | 0.186 |
| **Anderson-Darling** | **A-Sq** | 0.53395559 | **Pr > A-Sq** | 0.179 |

| **Quantiles for Normal Distribution** | | |
| --- | --- | --- |
| **Percent** | **Quantile** | |
|  | **Observed** | **Estimated** |
| **1.0** | -2.20543 | -2.08143 |
| **5.0** | -1.56928 | -1.47168 |
| **10.0** | -1.19188 | -1.14663 |
| **25.0** | -0.56623 | -0.60348 |
| **50.0** | 0.05527 | 0.00000 |
| **75.0** | 0.59288 | 0.60348 |
| **90.0** | 1.10552 | 1.14663 |
| **95.0** | 1.35035 | 1.47168 |
| **99.0** | 1.96234 | 2.08143 |

| NEPHI DRY-MATTER |
| --- |
| FALL 2011 |

The UNIVARIATE Procedure


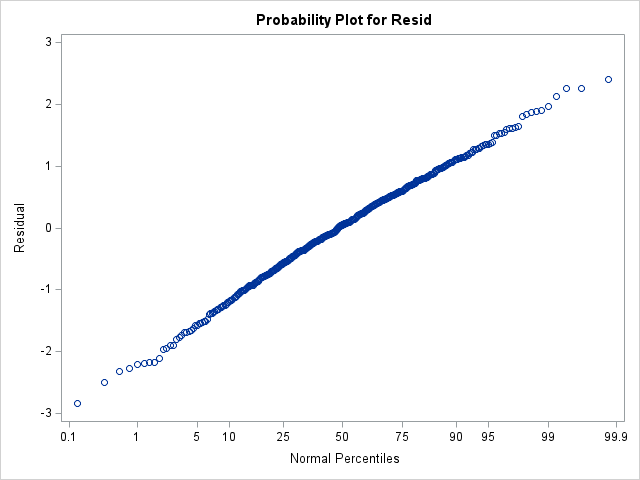


| NEPHI DRY-MATTER |
| --- |
| FALL 2011 |

The TRANSREG Procedure


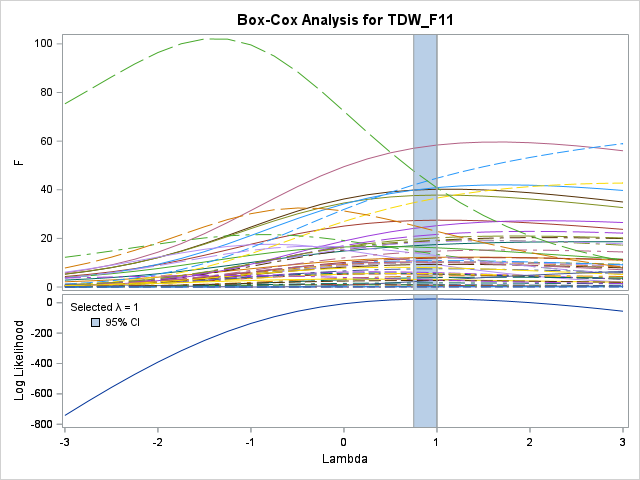


| NEPHI DRY-MATTER |
| --- |
| FALL 2011 |

The TRANSREG Procedure

| **TRANSREG Univariate Algorithm Iteration History for BoxCox(TDW_F11)** | | | | | |
| --- | --- | --- | --- | --- | --- |
| **Iteration Number** | **Average Change** | **Maximum Change** | **R-Square** | **Criterion Change** | **Note** |
| **1** | 0.03305 | 2.39605 | 0.65758 |  |  |
| **2** | 0.00775 | 0.34783 | 0.68101 | 0.02343 |  |
| **3** | 0.00193 | 0.07728 | 0.68167 | 0.00066 |  |
| **4** | 0.00055 | 0.02429 | 0.68172 | 0.00005 |  |
| **5** | 0.00017 | 0.00777 | 0.68172 | 0.00000 |  |
| **6** | 0.00005 | 0.00250 | 0.68172 | 0.00000 |  |
| **7** | 0.00002 | 0.00080 | 0.68172 | 0.00000 |  |
| **8** | 0.00001 | 0.00026 | 0.68172 | 0.00000 | Converged |

| Algorithm converged. |
| --- |

Appendix S2. SAS code (TOM289S1.sas) and output for least-squares means (LSmeans) for transformed total biomass (TTOTDW) at two locations across 2012-2016. To generate back-transformed LSmeans, apply the formula TOTDW = ((TTOTDW/2) + 1)^2^. Bluebunch wheatgrass statistics are presented in Table 4, and Snake River wheatgrass statistics are presented in Table 5.

FILENAME SCREEN DDE 'EXCEL|Sheet1!R5C1:R1408C15';

**DATA** ONE;

INFILE SCREEN LRECL=**5000**;

INPUT LOC $ REP PLOT NO_PLTS ENTRY $ DW_F11 DW_12 DW_13 SPK_13 PLTNO_14 DW_14 DW_15 DW_16 DW_11_16 X1;

OPTION PAGESIZE=**5000**; LRECL=**1000**;

%include 'c:\pdmix8002.SAS';

**DATA** ONE; SET ONE;

DW_F11=DW_F11*(**12**/NO_PLTS);

DW_12=DW_12*(**12**/NO_PLTS);

DW_13=DW_13*(**12**/NO_PLTS);

DW_14=DW_14*(**12**/PLTNO_14);

DW_15=DW_15*(**12**/PLTNO_14);

DW_16=DW_16*(**12**/PLTNO_14);

**DATA** ONE; SET ONE;

TOTDW=DW_12+DW_13+DW_14+DW_15+DW_16;

**DATA** ONE; SET ONE;

TTOTDW=((TOTDW****0.5**)-**1**)/**0.5**;

**DATA** NEPHI; SET ONE; IF LOC='Nephi';

**DATA** MV; SET ONE; IF LOC='Mville';

**PROC** **MIXED** DATA=NEPHI;

TITLE1 'NEPHI DRY-MATTER';

TITLE2 '2012 + 2013 + 2014 + 2015 + 2016';

CLASS REP ENTRY;

MODEL TTOTDW = ENTRY/OUTP=RESID3;

RANDOM REP;

LSMEANS ENTRY/PDIFF;

ODS OUTPUT DIFFS=AAA LSMEANS=BBB;

ODS EXCLUDE DIFFS;

**RUN**;

%***PDMIX800*** (AAA,BBB,ALPHA=**0.05**,SORT=YES)

%***PDMIX800*** (AAA,BBB,ALPHA=**0.10**,SORT=YES)

QUIT;

**RUN**;

**PROC** **UNIVARIATE** DATA=RESID3 NORMAL PLOT;

VAR RESID;

HISTOGRAM RESID/NORMAL NOFRAME;

PROBPLOT RESID/NORMAL NOFRAME;

**RUN**;

**PROC** **TRANSREG** DATA=NEPHI;

MODEL BOXCOX(TTOTDW)=CLASS(REP ENTRY);

**RUN**; **QUIT**; **RUN**;

**PROC** **MIXED** DATA=MV;

TITLE1 'MILLVILLE DRY-MATTER';

TITLE2 '2012 + 2013 + 2014 + 2015 + 2016';

CLASS REP ENTRY;

MODEL TTOTDW = ENTRY/OUTP=RESID3;

RANDOM REP;

LSMEANS ENTRY/PDIFF;

ODS OUTPUT DIFFS=CCC LSMEANS=DDD;

ODS EXCLUDE DIFFS;

**RUN**;

%***PDMIX800*** (CCC,DDD,ALPHA=**0.05**,SORT=YES)

%***PDMIX800*** (CCC,DDD,ALPHA=**0.10**,SORT=YES)

QUIT;

**RUN**;

**PROC** **UNIVARIATE** DATA=RESID3 NORMAL PLOT;

VAR RESID;

HISTOGRAM RESID/NORMAL NOFRAME;

PROBPLOT RESID/NORMAL NOFRAME;

**RUN**;

**PROC** **TRANSREG** DATA=MV;

MODEL BOXCOX(TTOTDW)=CLASS(REP ENTRY);

**RUN**; **QUIT**; **RUN**;

| NEPHI DRY-MATTER |
| --- |
| 2012 + 2013 + 2014 + 2015 + 2016 |

The Mixed Procedure

| **Model Information** | |
| --- | --- |
| **Data Set** | WORK.NEPHI |
| **Dependent Variable** | TTOTDW |
| **Covariance Structure** | Variance Components |
| **Estimation Method** | REML |
| **Residual Variance Method** | Profile |
| **Fixed Effects SE Method** | Model-Based |
| **Degrees of Freedom Method** | Containment |

| **Class Level Information** | | |
| --- | --- | --- |
| **Class** | **Levels** | **Values** |
| **REP** | 12 | 1 2 3 4 5 6 7 8 9 10 11 12 |
| **ENTRY** | 39 | Acc1156 Acc238 Acc243 Anatone Boardman Discover E49X E58X Goldar P10 P12 P15 P19 P20 P24 P25 P26 P30 P31 P32 P33 P34 P37 P40 P41 P42 P43 P44 P45 P7G3 P7G6 Secar Secar78 T1442 T1561 T1772 T655 Wahluke Whitmar |

| **Dimensions** | |
| --- | --- |
| **Covariance Parameters** | 2 |
| **Columns in X** | 40 |
| **Columns in Z** | 12 |
| **Subjects** | 1 |
| **Max Obs per Subject** | 453 |

| **Number of Observations** | |
| --- | --- |
| **Number of Observations Read** | 468 |
| **Number of Observations Used** | 453 |
| **Number of Observations Not Used** | 15 |

| **Iteration History** | | | |
| --- | --- | --- | --- |
| **Iteration** | **Evaluations** | **-2 Res Log Like** | **Criterion** |
| **0** | 1 | 3115.14801393 |  |
| **1** | 2 | 3110.70904128 | 0.00000000 |

| Convergence criteria met. |
| --- |

| **Covariance Parameter Estimates** | |
| --- | --- |
| **Cov Parm** | **Estimate** |
| **REP** | 2.7150 |
| **Residual** | 83.4130 |

| **Fit Statistics** | |
| --- | --- |
| **-2 Res Log Likelihood** | 3110.7 |
| **AIC (Smaller is Better)** | 3114.7 |
| **AICC (Smaller is Better)** | 3114.7 |
| **BIC (Smaller is Better)** | 3115.7 |

| **Type 3 Tests of Fixed Effects** | | | | |
| --- | --- | --- | --- | --- |
| **Effect** | **Num DF** | **Den DF** | **F Value** | **Pr > F** |
| **ENTRY** | 38 | 403 | 39.42 | <.0001 |

| **Least Squares Means** | | | | | | |
| --- | --- | --- | --- | --- | --- | --- |
| **Effect** | **ENTRY** | **Estimate** | **Standard Error** | **DF** | **t Value** | **Pr > \|t\|** |
| **ENTRY** | **Acc1156** | 81.5595 | 2.9307 | 403 | 27.83 | <.0001 |
| **ENTRY** | **Acc238** | 72.7919 | 2.6791 | 403 | 27.17 | <.0001 |
| **ENTRY** | **Acc243** | 64.6356 | 2.6791 | 403 | 24.13 | <.0001 |
| **ENTRY** | **Anatone** | 83.5553 | 2.6791 | 403 | 31.19 | <.0001 |
| **ENTRY** | **Boardman** | 38.0216 | 2.6791 | 403 | 14.19 | <.0001 |
| **ENTRY** | **Discover** | 87.2421 | 2.7962 | 403 | 31.20 | <.0001 |
| **ENTRY** | **E49X** | 86.4133 | 2.6791 | 403 | 32.26 | <.0001 |
| **ENTRY** | **E58X** | 94.7668 | 2.7962 | 403 | 33.89 | <.0001 |
| **ENTRY** | **Goldar** | 71.9303 | 2.6791 | 403 | 26.85 | <.0001 |
| **ENTRY** | **P10** | 87.0369 | 2.7962 | 403 | 31.13 | <.0001 |
| **ENTRY** | **P12** | 72.6674 | 2.9307 | 403 | 24.80 | <.0001 |
| **ENTRY** | **P15** | 82.8898 | 2.9306 | 403 | 28.28 | <.0001 |
| **ENTRY** | **P19** | 88.2573 | 2.6791 | 403 | 32.94 | <.0001 |
| **ENTRY** | **P20** | 83.6086 | 2.7962 | 403 | 29.90 | <.0001 |
| **ENTRY** | **P24** | 70.0878 | 2.6791 | 403 | 26.16 | <.0001 |
| **ENTRY** | **P25** | 75.4612 | 2.7963 | 403 | 26.99 | <.0001 |
| **ENTRY** | **P26** | 81.7640 | 2.6791 | 403 | 30.52 | <.0001 |
| **ENTRY** | **P30** | 48.0829 | 2.6791 | 403 | 17.95 | <.0001 |
| **ENTRY** | **P31** | 85.3919 | 2.6791 | 403 | 31.87 | <.0001 |
| **ENTRY** | **P32** | 80.2222 | 2.6791 | 403 | 29.94 | <.0001 |
| **ENTRY** | **P33** | 79.2568 | 2.6791 | 403 | 29.58 | <.0001 |
| **ENTRY** | **P34** | 88.9795 | 2.6791 | 403 | 33.21 | <.0001 |
| **ENTRY** | **P37** | 40.1816 | 2.6791 | 403 | 15.00 | <.0001 |
| **ENTRY** | **P40** | 83.9134 | 2.6791 | 403 | 31.32 | <.0001 |
| **ENTRY** | **P41** | 72.0789 | 2.6791 | 403 | 26.90 | <.0001 |
| **ENTRY** | **P42** | 85.0926 | 2.6791 | 403 | 31.76 | <.0001 |
| **ENTRY** | **P43** | 86.9969 | 2.6791 | 403 | 32.47 | <.0001 |
| **ENTRY** | **P44** | 85.7879 | 2.6791 | 403 | 32.02 | <.0001 |
| **ENTRY** | **P45** | 90.7320 | 2.6791 | 403 | 33.87 | <.0001 |
| **ENTRY** | **P7G3** | 87.2629 | 2.7963 | 403 | 31.21 | <.0001 |
| **ENTRY** | **P7G6** | 84.2911 | 2.6791 | 403 | 31.46 | <.0001 |
| **ENTRY** | **Secar** | 88.8547 | 2.7963 | 403 | 31.78 | <.0001 |
| **ENTRY** | **Secar78** | 87.4429 | 2.6791 | 403 | 32.64 | <.0001 |
| **ENTRY** | **T1442** | 40.3389 | 2.6791 | 403 | 15.06 | <.0001 |
| **ENTRY** | **T1561** | 30.9550 | 2.6791 | 403 | 11.55 | <.0001 |
| **ENTRY** | **T1772** | 52.3033 | 2.7963 | 403 | 18.70 | <.0001 |
| **ENTRY** | **T655** | 78.3180 | 2.7962 | 403 | 28.01 | <.0001 |
| **ENTRY** | **Wahluke** | 57.4419 | 2.6791 | 403 | 21.44 | <.0001 |
| **ENTRY** | **Whitmar** | 60.0902 | 2.6791 | 403 | 22.43 | <.0001 |

| NEPHI DRY-MATTER |
| --- |
| 2012 + 2013 + 2014 + 2015 + 2016 |

Effect=ENTRY Method=LSD(P<0.05) Set=1

| **Obs** | **ENTRY** | **Estimate** | **Standard Error** | **Letter Group** |
| --- | --- | --- | --- | --- |
| **1** | E58X | 94.7668 | 2.7962 | A |
| **2** | P45 | 90.7320 | 2.6791 | AB |
| **3** | P34 | 88.9795 | 2.6791 | ABC |
| **4** | Secar | 88.8547 | 2.7963 | ABC |
| **5** | P19 | 88.2573 | 2.6791 | ABC |
| **6** | Secar78 | 87.4429 | 2.6791 | ABCD |
| **7** | P7G3 | 87.2629 | 2.7963 | ABCD |
| **8** | Discover | 87.2421 | 2.7962 | ABCD |
| **9** | P10 | 87.0369 | 2.7962 | BCD |
| **10** | P43 | 86.9969 | 2.6791 | BCD |
| **11** | E49X | 86.4133 | 2.6791 | BCDE |
| **12** | P44 | 85.7879 | 2.6791 | BCDEF |
| **13** | P31 | 85.3919 | 2.6791 | BCDEF |
| **14** | P42 | 85.0926 | 2.6791 | BCDEF |
| **15** | P7G6 | 84.2911 | 2.6791 | BCDEF |
| **16** | P40 | 83.9134 | 2.6791 | BCDEF |
| **17** | P20 | 83.6086 | 2.7962 | BCDEF |
| **18** | Anatone | 83.5553 | 2.6791 | BCDEF |
| **19** | P15 | 82.8898 | 2.9306 | CDEFG |
| **20** | P26 | 81.7640 | 2.6791 | CDEFG |
| **21** | Acc1156 | 81.5595 | 2.9307 | CDEFG |
| **22** | P32 | 80.2222 | 2.6791 | DEFGH |
| **23** | P33 | 79.2568 | 2.6791 | EFGHI |
| **24** | T655 | 78.3180 | 2.7962 | FGHI |
| **25** | P25 | 75.4612 | 2.7963 | GHIJ |
| **26** | Acc238 | 72.7919 | 2.6791 | IJ |
| **27** | P12 | 72.6674 | 2.9307 | HIJ |
| **28** | P41 | 72.0789 | 2.6791 | IJ |
| **29** | Goldar | 71.9303 | 2.6791 | IJK |
| **30** | P24 | 70.0878 | 2.6791 | JK |
| **31** | Acc243 | 64.6356 | 2.6791 | KL |
| **32** | Whitmar | 60.0902 | 2.6791 | L |
| **33** | Wahluke | 57.4419 | 2.6791 | LM |
| **34** | T1772 | 52.3033 | 2.7963 | MN |
| **35** | P30 | 48.0829 | 2.6791 | N |
| **36** | T1442 | 40.3389 | 2.6791 | O |
| **37** | P37 | 40.1816 | 2.6791 | O |
| **38** | Boardman | 38.0216 | 2.6791 | OP |
| **39** | T1561 | 30.9550 | 2.6791 | P |

| NEPHI DRY-MATTER |
| --- |
| 2012 + 2013 + 2014 + 2015 + 2016 |

Effect=ENTRY Method=LSD(P<0.10) Set=1

| **Obs** | **ENTRY** | **Estimate** | **Standard Error** | **Letter Group** |
| --- | --- | --- | --- | --- |
| **1** | E58X | 94.7668 | 2.7962 | A |
| **2** | P45 | 90.7320 | 2.6791 | AB |
| **3** | P34 | 88.9795 | 2.6791 | ABC |
| **4** | Secar | 88.8547 | 2.7963 | ABC |
| **5** | P19 | 88.2573 | 2.6791 | BC |
| **6** | Secar78 | 87.4429 | 2.6791 | BCD |
| **7** | P7G3 | 87.2629 | 2.7963 | BCD |
| **8** | Discover | 87.2421 | 2.7962 | BCD |
| **9** | P10 | 87.0369 | 2.7962 | BCD |
| **10** | P43 | 86.9969 | 2.6791 | BCD |
| **11** | E49X | 86.4133 | 2.6791 | BCD |
| **12** | P44 | 85.7879 | 2.6791 | BCDE |
| **13** | P31 | 85.3919 | 2.6791 | BCDEF |
| **14** | P42 | 85.0926 | 2.6791 | BCDEF |
| **15** | P7G6 | 84.2911 | 2.6791 | CDEFG |
| **16** | P40 | 83.9134 | 2.6791 | CDEFG |
| **17** | P20 | 83.6086 | 2.7962 | CDEFG |
| **18** | Anatone | 83.5553 | 2.6791 | CDEFG |
| **19** | P15 | 82.8898 | 2.9306 | CDEFG |
| **20** | P26 | 81.7640 | 2.6791 | DEFG |
| **21** | Acc1156 | 81.5595 | 2.9307 | DEFGH |
| **22** | P32 | 80.2222 | 2.6791 | EFGH |
| **23** | P33 | 79.2568 | 2.6791 | FGH |
| **24** | T655 | 78.3180 | 2.7962 | GHI |
| **25** | P25 | 75.4612 | 2.7963 | HIJ |
| **26** | Acc238 | 72.7919 | 2.6791 | IJ |
| **27** | P12 | 72.6674 | 2.9307 | IJ |
| **28** | P41 | 72.0789 | 2.6791 | IJ |
| **29** | Goldar | 71.9303 | 2.6791 | J |
| **30** | P24 | 70.0878 | 2.6791 | JK |
| **31** | Acc243 | 64.6356 | 2.6791 | KL |
| **32** | Whitmar | 60.0902 | 2.6791 | LM |
| **33** | Wahluke | 57.4419 | 2.6791 | MN |
| **34** | T1772 | 52.3033 | 2.7963 | NO |
| **35** | P30 | 48.0829 | 2.6791 | O |
| **36** | T1442 | 40.3389 | 2.6791 | P |
| **37** | P37 | 40.1816 | 2.6791 | P |
| **38** | Boardman | 38.0216 | 2.6791 | P |
| **39** | T1561 | 30.9550 | 2.6791 | Q |

| NEPHI DRY-MATTER |
| --- |
| 2012 + 2013 + 2014 + 2015 + 2016 |

The UNIVARIATE Procedure

Variable: Resid (Residual)

| **Moments** | | | |
| --- | --- | --- | --- |
| **N** | 453 | **Sum Weights** | 453 |
| **Mean** | 0 | **Sum Observations** | 0 |
| **Std Deviation** | 8.67661345 | **Variance** | 75.283621 |
| **Skewness** | -0.2019626 | **Kurtosis** | 0.37007524 |
| **Uncorrected SS** | 34028.1967 | **Corrected SS** | 34028.1967 |
| **Coeff Variation** | . | **Std Error Mean** | 0.40766286 |

| **Basic Statistical Measures** | | | |
| --- | --- | --- | --- |
| **Location** | | **Variability** | |
| **Mean** | 0.00000 | **Std Deviation** | 8.67661 |
| **Median** | -0.11711 | **Variance** | 75.28362 |
| **Mode** | . | **Range** | 52.64362 |
|  |  | **Interquartile Range** | 10.52391 |

| **Tests for Location: Mu0=0** | | | | |
| --- | --- | --- | --- | --- |
| **Test** | **Statistic** | | **p Value** | |
| **Student's t** | **t** | 0 | **Pr > \|t\|** | 1.0000 |
| **Sign** | **M** | -3.5 | **Pr >= \|M\|** | 0.7781 |
| **Signed Rank** | **S** | 1045.5 | **Pr >= \|S\|** | 0.7081 |

| **Tests for Normality** | | | | |
| --- | --- | --- | --- | --- |
| **Test** | **Statistic** | | **p Value** | |
| **Shapiro-Wilk** | **W** | 0.993547 | **Pr < W** | 0.0503 |
| **Kolmogorov-Smirnov** | **D** | 0.053607 | **Pr > D** | <0.0100 |
| **Cramer-von Mises** | **W-Sq** | 0.156934 | **Pr > W-Sq** | 0.0204 |
| **Anderson-Darling** | **A-Sq** | 0.925504 | **Pr > A-Sq** | 0.0200 |

| **Quantiles (Definition 5)** | |
| --- | --- |
| **Level** | **Quantile** |
| **100% Max** | 24.774212 |
| **99%** | 20.929349 |
| **95%** | 14.782053 |
| **90%** | 10.454723 |
| **75% Q3** | 5.730017 |
| **50% Median** | -0.117109 |
| **25% Q1** | -4.793897 |
| **10%** | -11.207477 |
| **5%** | -15.682271 |
| **1%** | -23.248921 |
| **0% Min** | -27.869413 |

| **Extreme Observations** | | | |
| --- | --- | --- | --- |
| **Lowest** | | **Highest** | |
| **Value** | **Obs** | **Value** | **Obs** |
| -27.8694 | 258 | 20.9293 | 425 |
| -26.4829 | 286 | 20.9870 | 320 |
| -25.2690 | 78 | 22.1888 | 246 |
| -23.6260 | 208 | 23.1320 | 325 |
| -23.2489 | 371 | 24.7742 | 372 |

| **Missing Values** | | | |
| --- | --- | --- | --- |
| **Missing Value** | **Count** | **Percent Of** | |
|  |  | **All Obs** | **Missing Obs** |
| . | 15 | 3.21 | 100.00 |


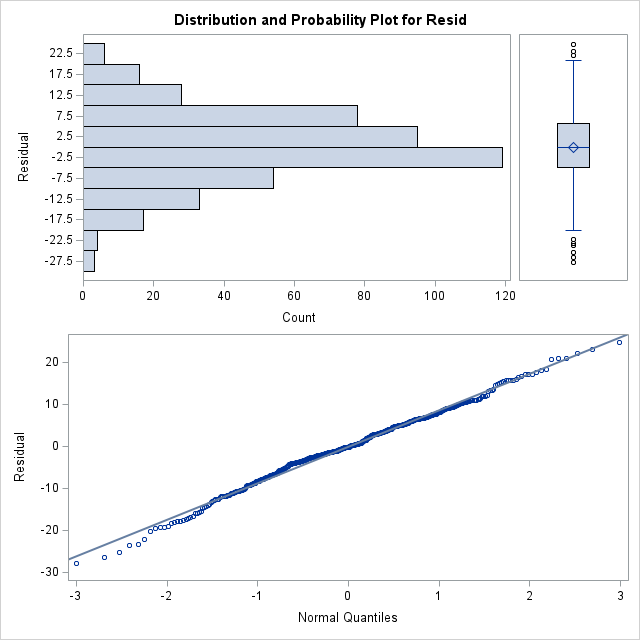


| NEPHI DRY-MATTER |
| --- |
| 2012 + 2013 + 2014 + 2015 + 2016 |

The UNIVARIATE Procedure


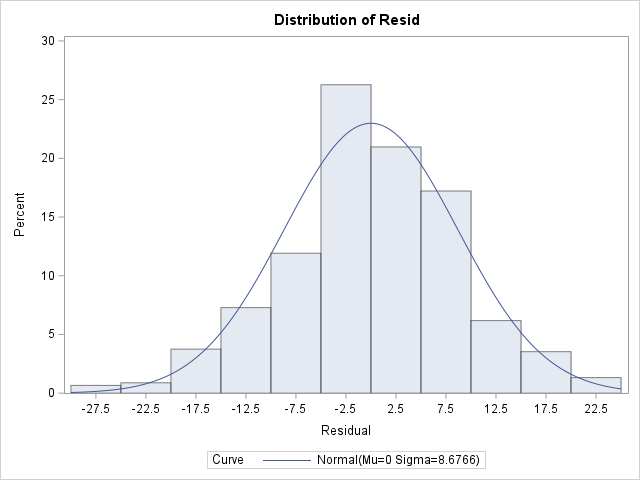


| NEPHI DRY-MATTER |
| --- |
| 2012 + 2013 + 2014 + 2015 + 2016 |

The UNIVARIATE Procedure

Fitted Normal Distribution for Resid (Residual)

| **Parameters for Normal Distribution** | | |
| --- | --- | --- |
| **Parameter** | **Symbol** | **Estimate** |
| **Mean** | Mu | 0 |
| **Std Dev** | Sigma | 8.676613 |

| **Goodness-of-Fit Tests for Normal Distribution** | | | | |
| --- | --- | --- | --- | --- |
| **Test** | **Statistic** | | **p Value** | |
| **Kolmogorov-Smirnov** | **D** | 0.05360672 | **Pr > D** | <0.010 |
| **Cramer-von Mises** | **W-Sq** | 0.15693440 | **Pr > W-Sq** | 0.020 |
| **Anderson-Darling** | **A-Sq** | 0.92550367 | **Pr > A-Sq** | 0.020 |

| **Quantiles for Normal Distribution** | | |
| --- | --- | --- |
| **Percent** | **Quantile** | |
|  | **Observed** | **Estimated** |
| **1.0** | -23.24892 | -20.1848 |
| **5.0** | -15.68227 | -14.2718 |
| **10.0** | -11.20748 | -11.1195 |
| **25.0** | -4.79390 | -5.8523 |
| **50.0** | -0.11711 | 0.0000 |
| **75.0** | 5.73002 | 5.8523 |
| **90.0** | 10.45472 | 11.1195 |
| **95.0** | 14.78205 | 14.2718 |
| **99.0** | 20.92935 | 20.1848 |

| NEPHI DRY-MATTER |
| --- |
| 2012 + 2013 + 2014 + 2015 + 2016 |

The UNIVARIATE Procedure


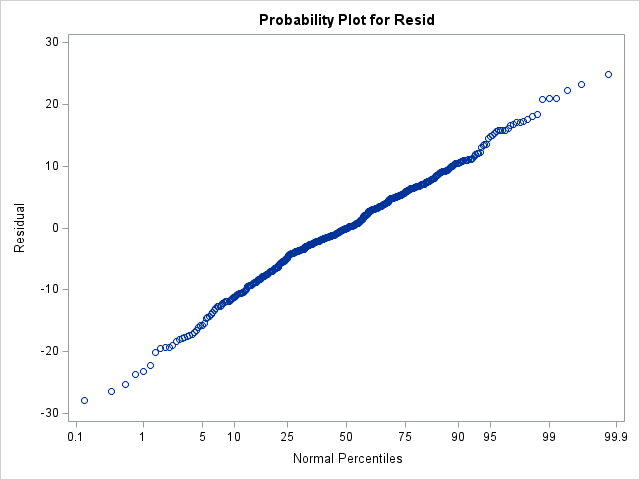


| NEPHI DRY-MATTER |
| --- |
| 2012 + 2013 + 2014 + 2015 + 2016 |

The TRANSREG Procedure


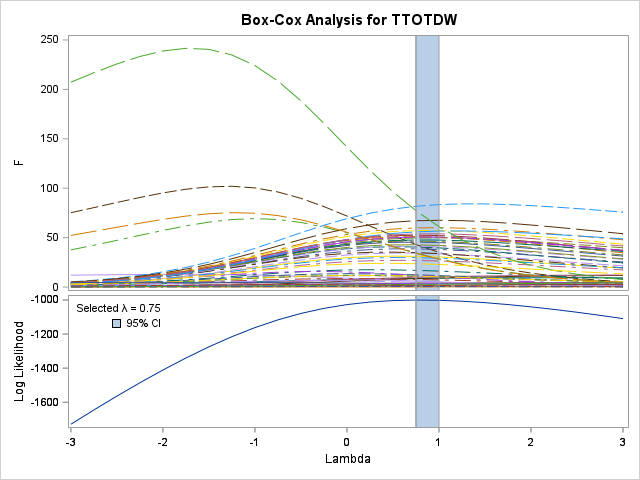


| NEPHI DRY-MATTER |
| --- |
| 2012 + 2013 + 2014 + 2015 + 2016 |

The TRANSREG Procedure

| **TRANSREG Univariate Algorithm Iteration History for BoxCox(TTOTDW)** | | | | | |
| --- | --- | --- | --- | --- | --- |
| **Iteration Number** | **Average Change** | **Maximum Change** | **R-Square** | **Criterion Change** | **Note** |
| **1** | 0.02329 | 1.45200 | 0.79394 |  |  |
| **2** | 0.00287 | 0.15333 | 0.80577 | 0.01183 |  |
| **3** | 0.00032 | 0.01715 | 0.80590 | 0.00013 |  |
| **4** | 0.00004 | 0.00313 | 0.80590 | 0.00000 |  |
| **5** | 0.00001 | 0.00065 | 0.80590 | 0.00000 | Converged |

| Algorithm converged. |
| --- |

| MILLVILLE DRY-MATTER |
| --- |
| 2012 + 2013 + 2014 + 2015 + 2016 |

The Mixed Procedure

| **Model Information** | |
| --- | --- |
| **Data Set** | WORK.MV |
| **Dependent Variable** | TTOTDW |
| **Covariance Structure** | Variance Components |
| **Estimation Method** | REML |
| **Residual Variance Method** | Profile |
| **Fixed Effects SE Method** | Model-Based |
| **Degrees of Freedom Method** | Containment |

| **Class Level Information** | | |
| --- | --- | --- |
| **Class** | **Levels** | **Values** |
| **REP** | 12 | 1 2 3 4 5 6 7 8 9 10 11 12 |
| **ENTRY** | 39 | Acc1156 Acc238 Acc243 Anatone Boardman Discover E49X E58X Goldar P10 P12 P15 P19 P20 P24 P25 P26 P30 P31 P32 P33 P34 P37 P40 P41 P42 P43 P44 P45 P7G3 P7G6 Secar Secar78 T1442 T1561 T1772 T655 Wahluke Whitmar |

| **Dimensions** | |
| --- | --- |
| **Covariance Parameters** | 2 |
| **Columns in X** | 40 |
| **Columns in Z** | 12 |
| **Subjects** | 1 |
| **Max Obs per Subject** | 447 |

| **Number of Observations** | |
| --- | --- |
| **Number of Observations Read** | 468 |
| **Number of Observations Used** | 447 |
| **Number of Observations Not Used** | 21 |

| **Iteration History** | | | |
| --- | --- | --- | --- |
| **Iteration** | **Evaluations** | **-2 Res Log Like** | **Criterion** |
| **0** | 1 | 3044.97045456 |  |
| **1** | 2 | 2944.64247925 | 0.00000322 |
| **2** | 1 | 2944.63883605 | 0.00000001 |

| Convergence criteria met. |
| --- |

| **Covariance Parameter Estimates** | |
| --- | --- |
| **Cov Parm** | **Estimate** |
| **REP** | 23.3849 |
| **Residual** | 58.6865 |

| **Fit Statistics** | |
| --- | --- |
| **-2 Res Log Likelihood** | 2944.6 |
| **AIC (Smaller is Better)** | 2948.6 |
| **AICC (Smaller is Better)** | 2948.7 |
| **BIC (Smaller is Better)** | 2949.6 |

| **Type 3 Tests of Fixed Effects** | | | | |
| --- | --- | --- | --- | --- |
| **Effect** | **Num DF** | **Den DF** | **F Value** | **Pr > F** |
| **ENTRY** | 38 | 397 | 41.58 | <.0001 |

| **Least Squares Means** | | | | | | |
| --- | --- | --- | --- | --- | --- | --- |
| **Effect** | **ENTRY** | **Estimate** | **Standard Error** | **DF** | **t Value** | **Pr > \|t\|** |
| **ENTRY** | **Acc1156** | 83.7464 | 2.7017 | 397 | 31.00 | <.0001 |
| **ENTRY** | **Acc238** | 77.8281 | 2.8014 | 397 | 27.78 | <.0001 |
| **ENTRY** | **Acc243** | 71.9739 | 2.7017 | 397 | 26.64 | <.0001 |
| **ENTRY** | **Anatone** | 94.4795 | 2.6152 | 397 | 36.13 | <.0001 |
| **ENTRY** | **Boardman** | 45.8862 | 2.6152 | 397 | 17.55 | <.0001 |
| **ENTRY** | **Discover** | 85.2263 | 2.6152 | 397 | 32.59 | <.0001 |
| **ENTRY** | **E49X** | 69.4973 | 2.7017 | 397 | 25.72 | <.0001 |
| **ENTRY** | **E58X** | 83.5769 | 2.8014 | 397 | 29.83 | <.0001 |
| **ENTRY** | **Goldar** | 86.6577 | 2.6152 | 397 | 33.14 | <.0001 |
| **ENTRY** | **P10** | 99.6062 | 2.8010 | 397 | 35.56 | <.0001 |
| **ENTRY** | **P12** | 76.8947 | 2.7017 | 397 | 28.46 | <.0001 |
| **ENTRY** | **P15** | 84.0710 | 2.7017 | 397 | 31.12 | <.0001 |
| **ENTRY** | **P19** | 98.6752 | 2.7013 | 397 | 36.53 | <.0001 |
| **ENTRY** | **P20** | 85.8971 | 2.6152 | 397 | 32.85 | <.0001 |
| **ENTRY** | **P24** | 80.7759 | 2.6152 | 397 | 30.89 | <.0001 |
| **ENTRY** | **P25** | 89.4474 | 2.6152 | 397 | 34.20 | <.0001 |
| **ENTRY** | **P26** | 92.7480 | 2.6152 | 397 | 35.46 | <.0001 |
| **ENTRY** | **P30** | 57.2374 | 2.6152 | 397 | 21.89 | <.0001 |
| **ENTRY** | **P31** | 88.0450 | 2.6152 | 397 | 33.67 | <.0001 |
| **ENTRY** | **P32** | 87.6715 | 2.6152 | 397 | 33.52 | <.0001 |
| **ENTRY** | **P33** | 90.5240 | 2.6152 | 397 | 34.61 | <.0001 |
| **ENTRY** | **P34** | 97.2572 | 2.7013 | 397 | 36.00 | <.0001 |
| **ENTRY** | **P37** | 60.0084 | 2.6152 | 397 | 22.95 | <.0001 |
| **ENTRY** | **P40** | 93.0834 | 2.7017 | 397 | 34.45 | <.0001 |
| **ENTRY** | **P41** | 89.9127 | 2.6152 | 397 | 34.38 | <.0001 |
| **ENTRY** | **P42** | 94.1661 | 2.6152 | 397 | 36.01 | <.0001 |
| **ENTRY** | **P43** | 82.4086 | 2.6152 | 397 | 31.51 | <.0001 |
| **ENTRY** | **P44** | 94.4172 | 2.6152 | 397 | 36.10 | <.0001 |
| **ENTRY** | **P45** | 96.9184 | 2.6152 | 397 | 37.06 | <.0001 |
| **ENTRY** | **P7G3** | 92.4294 | 2.6152 | 397 | 35.34 | <.0001 |
| **ENTRY** | **P7G6** | 92.5633 | 2.6152 | 397 | 35.39 | <.0001 |
| **ENTRY** | **Secar** | 82.8679 | 2.6152 | 397 | 31.69 | <.0001 |
| **ENTRY** | **Secar78** | 85.1972 | 2.6152 | 397 | 32.58 | <.0001 |
| **ENTRY** | **T1442** | 52.5062 | 2.6152 | 397 | 20.08 | <.0001 |
| **ENTRY** | **T1561** | 47.1974 | 2.8014 | 397 | 16.85 | <.0001 |
| **ENTRY** | **T1772** | 63.9445 | 2.8014 | 397 | 22.83 | <.0001 |
| **ENTRY** | **T655** | 88.3997 | 2.8014 | 397 | 31.56 | <.0001 |
| **ENTRY** | **Wahluke** | 55.0546 | 2.7009 | 397 | 20.38 | <.0001 |
| **ENTRY** | **Whitmar** | 76.3182 | 2.6152 | 397 | 29.18 | <.0001 |

| MILLVILLE DRY-MATTER |
| --- |
| 2012 + 2013 + 2014 + 2015 + 2016 |

Effect=ENTRY Method=LSD(P<0.05) Set=1

| **Obs** | **ENTRY** | **Estimate** | **Standard Error** | **Letter Group** |
| --- | --- | --- | --- | --- |
| **1** | P10 | 99.6062 | 2.8010 | A |
| **2** | P19 | 98.6752 | 2.7013 | AB |
| **3** | P34 | 97.2572 | 2.7013 | AB |
| **4** | P45 | 96.9184 | 2.6152 | AB |
| **5** | Anatone | 94.4795 | 2.6152 | ABC |
| **6** | P44 | 94.4172 | 2.6152 | ABC |
| **7** | P42 | 94.1661 | 2.6152 | ABCD |
| **8** | P40 | 93.0834 | 2.7017 | ABCDE |
| **9** | P26 | 92.7480 | 2.6152 | BCDEF |
| **10** | P7G6 | 92.5633 | 2.6152 | BCDEF |
| **11** | P7G3 | 92.4294 | 2.6152 | BCDEF |
| **12** | P33 | 90.5240 | 2.6152 | CDEFG |
| **13** | P41 | 89.9127 | 2.6152 | CDEFGH |
| **14** | P25 | 89.4474 | 2.6152 | CDEFGH |
| **15** | T655 | 88.3997 | 2.8014 | CDEFGHI |
| **16** | P31 | 88.0450 | 2.6152 | DEFGHI |
| **17** | P32 | 87.6715 | 2.6152 | EFGHI |
| **18** | Goldar | 86.6577 | 2.6152 | FGHIJ |
| **19** | P20 | 85.8971 | 2.6152 | GHIJ |
| **20** | Discover | 85.2263 | 2.6152 | GHIJ |
| **21** | Secar78 | 85.1972 | 2.6152 | GHIJ |
| **22** | P15 | 84.0710 | 2.7017 | HIJK |
| **23** | Acc1156 | 83.7464 | 2.7017 | HIJK |
| **24** | E58X | 83.5769 | 2.8014 | HIJK |
| **25** | Secar | 82.8679 | 2.6152 | IJKL |
| **26** | P43 | 82.4086 | 2.6152 | IJKLM |
| **27** | P24 | 80.7759 | 2.6152 | JKLM |
| **28** | Acc238 | 77.8281 | 2.8014 | KLMN |
| **29** | P12 | 76.8947 | 2.7017 | LMN |
| **30** | Whitmar | 76.3182 | 2.6152 | MN |
| **31** | Acc243 | 71.9739 | 2.7017 | NO |
| **32** | E49X | 69.4973 | 2.7017 | OP |
| **33** | T1772 | 63.9445 | 2.8014 | PQ |
| **34** | P37 | 60.0084 | 2.6152 | QR |
| **35** | P30 | 57.2374 | 2.6152 | RS |
| **36** | Wahluke | 55.0546 | 2.7009 | RS |
| **37** | T1442 | 52.5062 | 2.6152 | ST |
| **38** | T1561 | 47.1974 | 2.8014 | TU |
| **39** | Boardman | 45.8862 | 2.6152 | U |

| MILLVILLE DRY-MATTER |
| --- |
| 2012 + 2013 + 2014 + 2015 + 2016 |

Effect=ENTRY Method=LSD(P<0.10) Set=1

| **Obs** | **ENTRY** | **Estimate** | **Standard Error** | **Letter Group** |
| --- | --- | --- | --- | --- |
| **1** | P10 | 99.6062 | 2.8010 | A |
| **2** | P19 | 98.6752 | 2.7013 | AB |
| **3** | P34 | 97.2572 | 2.7013 | ABC |
| **4** | P45 | 96.9184 | 2.6152 | ABC |
| **5** | Anatone | 94.4795 | 2.6152 | ABCD |
| **6** | P44 | 94.4172 | 2.6152 | ABCD |
| **7** | P42 | 94.1661 | 2.6152 | BCD |
| **8** | P40 | 93.0834 | 2.7017 | CDE |
| **9** | P26 | 92.7480 | 2.6152 | CDEF |
| **10** | P7G6 | 92.5633 | 2.6152 | CDEF |
| **11** | P7G3 | 92.4294 | 2.6152 | CDEF |
| **12** | P33 | 90.5240 | 2.6152 | DEFG |
| **13** | P41 | 89.9127 | 2.6152 | DEFGH |
| **14** | P25 | 89.4474 | 2.6152 | DEFGH |
| **15** | T655 | 88.3997 | 2.8014 | EFGHI |
| **16** | P31 | 88.0450 | 2.6152 | EFGHI |
| **17** | P32 | 87.6715 | 2.6152 | FGHIJ |
| **18** | Goldar | 86.6577 | 2.6152 | GHIJK |
| **19** | P20 | 85.8971 | 2.6152 | GHIJKL |
| **20** | Discover | 85.2263 | 2.6152 | HIJKL |
| **21** | Secar78 | 85.1972 | 2.6152 | HIJKL |
| **22** | P15 | 84.0710 | 2.7017 | IJKL |
| **23** | Acc1156 | 83.7464 | 2.7017 | IJKL |
| **24** | E58X | 83.5769 | 2.8014 | IJKL |
| **25** | Secar | 82.8679 | 2.6152 | JKLM |
| **26** | P43 | 82.4086 | 2.6152 | KLM |
| **27** | P24 | 80.7759 | 2.6152 | LMN |
| **28** | Acc238 | 77.8281 | 2.8014 | MN |
| **29** | P12 | 76.8947 | 2.7017 | NO |
| **30** | Whitmar | 76.3182 | 2.6152 | NO |
| **31** | Acc243 | 71.9739 | 2.7017 | OP |
| **32** | E49X | 69.4973 | 2.7017 | P |
| **33** | T1772 | 63.9445 | 2.8014 | Q |
| **34** | P37 | 60.0084 | 2.6152 | QR |
| **35** | P30 | 57.2374 | 2.6152 | RS |
| **36** | Wahluke | 55.0546 | 2.7009 | RS |
| **37** | T1442 | 52.5062 | 2.6152 | ST |
| **38** | T1561 | 47.1974 | 2.8014 | TU |
| **39** | Boardman | 45.8862 | 2.6152 | U |

| MILLVILLE DRY-MATTER |
| --- |
| 2012 + 2013 + 2014 + 2015 + 2016 |

The UNIVARIATE Procedure

Variable: Resid (Residual)

| **Moments** | | | |
| --- | --- | --- | --- |
| **N** | 447 | **Sum Weights** | 447 |
| **Mean** | 0 | **Sum Observations** | 0 |
| **Std Deviation** | 7.23394758 | **Variance** | 52.3299975 |
| **Skewness** | 0.05326526 | **Kurtosis** | -0.0722745 |
| **Uncorrected SS** | 23339.1789 | **Corrected SS** | 23339.1789 |
| **Coeff Variation** | . | **Std Error Mean** | 0.34215398 |

| **Basic Statistical Measures** | | | |
| --- | --- | --- | --- |
| **Location** | | **Variability** | |
| **Mean** | 0.00000 | **Std Deviation** | 7.23395 |
| **Median** | -0.45498 | **Variance** | 52.33000 |
| **Mode** | . | **Range** | 43.80545 |
|  |  | **Interquartile Range** | 9.90742 |

| **Tests for Location: Mu0=0** | | | | |
| --- | --- | --- | --- | --- |
| **Test** | **Statistic** | | **p Value** | |
| **Student's t** | **t** | 0 | **Pr > \|t\|** | 1.0000 |
| **Sign** | **M** | -12.5 | **Pr >= \|M\|** | 0.2563 |
| **Signed Rank** | **S** | -477 | **Pr >= \|S\|** | 0.8617 |

| **Tests for Normality** | | | | |
| --- | --- | --- | --- | --- |
| **Test** | **Statistic** | | **p Value** | |
| **Shapiro-Wilk** | **W** | 0.995117 | **Pr < W** | 0.1721 |
| **Kolmogorov-Smirnov** | **D** | 0.04896 | **Pr > D** | <0.0100 |
| **Cramer-von Mises** | **W-Sq** | 0.149563 | **Pr > W-Sq** | 0.0241 |
| **Anderson-Darling** | **A-Sq** | 0.852939 | **Pr > A-Sq** | 0.0288 |

| **Quantiles (Definition 5)** | |
| --- | --- |
| **Level** | **Quantile** |
| **100% Max** | 21.624879 |
| **99%** | 16.194044 |
| **95%** | 12.073361 |
| **90%** | 10.055656 |
| **75% Q3** | 4.972823 |
| **50% Median** | -0.454982 |
| **25% Q1** | -4.934598 |
| **10%** | -8.651246 |
| **5%** | -10.959151 |
| **1%** | -17.979057 |
| **0% Min** | -22.180575 |

| **Extreme Observations** | | | |
| --- | --- | --- | --- |
| **Lowest** | | **Highest** | |
| **Value** | **Obs** | **Value** | **Obs** |
| -22.1806 | 138 | 16.1940 | 369 |
| -19.5787 | 156 | 17.9825 | 254 |
| -19.2991 | 174 | 18.5415 | 168 |
| -19.1603 | 68 | 20.2837 | 6 |
| -17.9791 | 23 | 21.6249 | 128 |

| **Missing Values** | | | |
| --- | --- | --- | --- |
| **Missing Value** | **Count** | **Percent Of** | |
|  |  | **All Obs** | **Missing Obs** |
| . | 21 | 4.49 | 100.00 |


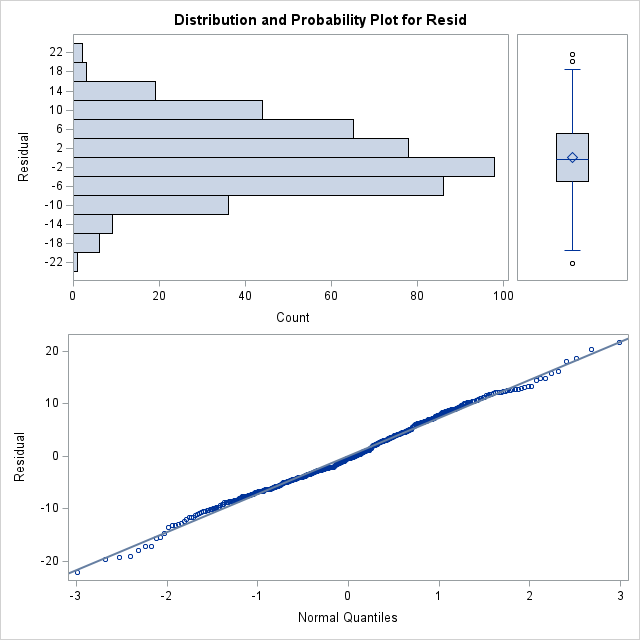


| MILLVILLE DRY-MATTER |
| --- |
| 2012 + 2013 + 2014 + 2015 + 2016 |

The UNIVARIATE Procedure


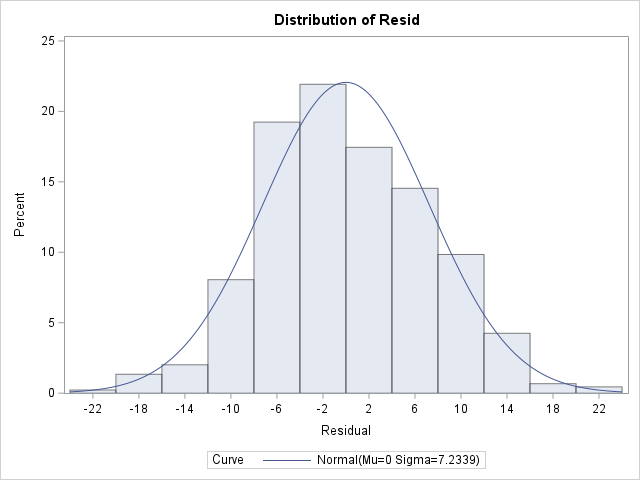


| MILLVILLE DRY-MATTER |
| --- |
| 2012 + 2013 + 2014 + 2015 + 2016 |

The UNIVARIATE Procedure

Fitted Normal Distribution for Resid (Residual)

| **Parameters for Normal Distribution** | | |
| --- | --- | --- |
| **Parameter** | **Symbol** | **Estimate** |
| **Mean** | Mu | 0 |
| **Std Dev** | Sigma | 7.233948 |

| **Goodness-of-Fit Tests for Normal Distribution** | | | | |
| --- | --- | --- | --- | --- |
| **Test** | **Statistic** | | **p Value** | |
| **Kolmogorov-Smirnov** | **D** | 0.04895953 | **Pr > D** | <0.010 |
| **Cramer-von Mises** | **W-Sq** | 0.14956255 | **Pr > W-Sq** | 0.024 |
| **Anderson-Darling** | **A-Sq** | 0.85293947 | **Pr > A-Sq** | 0.029 |

| **Quantiles for Normal Distribution** | | |
| --- | --- | --- |
| **Percent** | **Quantile** | |
|  | **Observed** | **Estimated** |
| **1.0** | -17.97906 | -16.8287 |
| **5.0** | -10.95915 | -11.8988 |
| **10.0** | -8.65125 | -9.2707 |
| **25.0** | -4.93460 | -4.8792 |
| **50.0** | -0.45498 | 0.0000 |
| **75.0** | 4.97282 | 4.8792 |
| **90.0** | 10.05566 | 9.2707 |
| **95.0** | 12.07336 | 11.8988 |
| **99.0** | 16.19404 | 16.8287 |

| MILLVILLE DRY-MATTER |
| --- |
| 2012 + 2013 + 2014 + 2015 + 2016 |

The UNIVARIATE Procedure


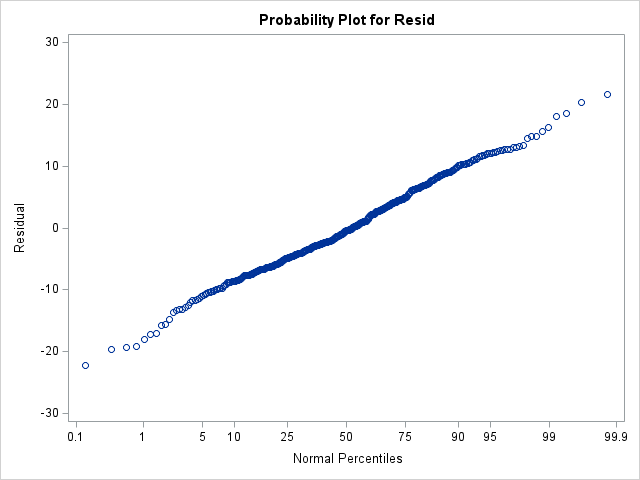


| MILLVILLE DRY-MATTER |
| --- |
| 2012 + 2013 + 2014 + 2015 + 2016 |

The TRANSREG Procedure


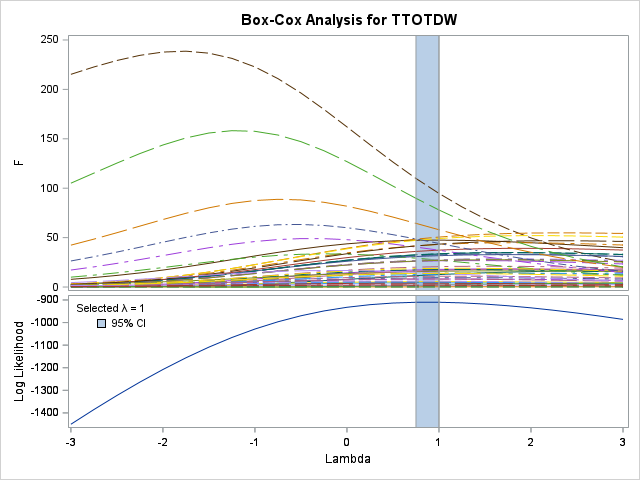


| MILLVILLE DRY-MATTER |
| --- |
| 2012 + 2013 + 2014 + 2015 + 2016 |

The TRANSREG Procedure

| **TRANSREG Univariate Algorithm Iteration History for BoxCox(TTOTDW)** | | | | | |
| --- | --- | --- | --- | --- | --- |
| **Iteration Number** | **Average Change** | **Maximum Change** | **R-Square** | **Criterion Change** | **Note** |
| **1** | 0.01013 | 1.58537 | 0.81029 |  |  |
| **2** | 0.00145 | 0.19097 | 0.81721 | 0.00692 |  |
| **3** | 0.00017 | 0.02314 | 0.81730 | 0.00009 |  |
| **4** | 0.00002 | 0.00283 | 0.81730 | 0.00000 |  |
| **5** | 0.00000 | 0.00035 | 0.81730 | 0.00000 | Converged |

| Algorithm converged. |
| --- |

Appendix S3. SAS code (TOM289ZZH.sas) and output for standard errors (s.e.) for biomass in 2011 (DW_F11) at three locations and across 2012-2016 (TOTDW) at two locations. Bluebunch wheatgrass statistics are presented in Table 4, and Snake River wheatgrass statistics are presented in Table 5.

FILENAME SCREEN DDE 'EXCEL|Sheet1!R5C1:R1408C13';

**DATA** ONE;

INFILE SCREEN LRECL=**5000**;

INPUT LOC $ REP PLOT NO_PLTS ENTRY $ DW_F11 DW_12 DW_13 SPK13 NO_PL14 DW_14 DW_15 DW_16;

OPTION PAGESIZE=**5000**; LRECL=**1000**;

%include 'c:\pdmix8002.SAS';

**DATA** ONE; SET ONE;

DW_F11=DW_F11*(**12**/NO_PLTS);

**DATA** ONE; SET ONE;

IF LOC='Nephi' AND REP=**1** AND PLOT=**1** THEN DW_F11=**.**;

IF LOC='Nephi' AND REP=**2** AND PLOT=**27** THEN DW_F11=**.**;

IF LOC='Nephi' AND REP=**7** AND PLOT=**39** THEN DW_F11=**.**;

**DATA** ONE; SET ONE;

DW_F11=DW_F11*(**12**/NO_PLTS);

DW_F12=DW_F12*(**12**/NO_PLTS);

DW_F13=DW_F13*(**12**/NO_PLTS);

DW_F14=DW_F14*(**12**/NO_PLTS);

DW_F15=DW_F15*(**12**/NO_PLTS);

DW_F16=DW_F16*(**12**/NO_PLTS);

**DATA** ONE; SET ONE;

TOTDW=DW_12 + DW_13 + DW_14 + DW_15 + DW_16;

**PROC** **SORT** DATA=ONE; BY LOC ENTRY;

**PROC** **MEANS** DATA=ONE N STDERR; BY LOC ENTRY; VAR DW_F11 TOTDW;

**RUN**; **QUIT**; **RUN**;

| The SAS System |
| --- |

The MEANS Procedure

LOC=Mville ENTRY=' '

| **Variable** | **N** | **Std Error** |
| --- | --- | --- |
| \| **DW_F11** \| \| --- \| \| **TOTDW** \| | \| 0 \| \| --- \| \| 0 \| | \| . \| \| --- \| \| . \| |

LOC=Mville ENTRY=Acc1156

| **Variable** | **N** | **Std Error** |
| --- | --- | --- |
| \| **DW_F11** \| \| --- \| \| **TOTDW** \| | \| 12 \| \| --- \| \| 11 \| | \| 5.3119079 \| \| --- \| \| 137.4374285 \| |

LOC=Mville ENTRY=Acc238

| **Variable** | **N** | **Std Error** |
| --- | --- | --- |
| \| **DW_F11** \| \| --- \| \| **TOTDW** \| | \| 10 \| \| --- \| \| 10 \| | \| 4.0058688 \| \| --- \| \| 83.5473465 \| |

LOC=Mville ENTRY=Acc243

| **Variable** | **N** | **Std Error** |
| --- | --- | --- |
| \| **DW_F11** \| \| --- \| \| **TOTDW** \| | \| 11 \| \| --- \| \| 11 \| | \| 4.1001576 \| \| --- \| \| 86.8672513 \| |

LOC=Mville ENTRY=Anatone

| **Variable** | **N** | **Std Error** |
| --- | --- | --- |
| \| **DW_F11** \| \| --- \| \| **TOTDW** \| | \| 12 \| \| --- \| \| 12 \| | \| 4.1156784 \| \| --- \| \| 126.1934574 \| |

LOC=Mville ENTRY=Boardman

| **Variable** | **N** | **Std Error** |
| --- | --- | --- |
| \| **DW_F11** \| \| --- \| \| **TOTDW** \| | \| 12 \| \| --- \| \| 12 \| | \| 3.6387096 \| \| --- \| \| 77.6087922 \| |

LOC=Mville ENTRY=Discover

| **Variable** | **N** | **Std Error** |
| --- | --- | --- |
| \| **DW_F11** \| \| --- \| \| **TOTDW** \| | \| 12 \| \| --- \| \| 12 \| | \| 2.4379583 \| \| --- \| \| 134.2961551 \| |

LOC=Mville ENTRY=E49X

| **Variable** | **N** | **Std Error** |
| --- | --- | --- |
| \| **DW_F11** \| \| --- \| \| **TOTDW** \| | \| 11 \| \| --- \| \| 11 \| | \| 5.8265778 \| \| --- \| \| 117.8170132 \| |

LOC=Mville ENTRY=E58X

| **Variable** | **N** | **Std Error** |
| --- | --- | --- |
| \| **DW_F11** \| \| --- \| \| **TOTDW** \| | \| 10 \| \| --- \| \| 10 \| | \| 3.8744400 \| \| --- \| \| 83.2862953 \| |

LOC=Mville ENTRY=Goldar

| **Variable** | **N** | **Std Error** |
| --- | --- | --- |
| \| **DW_F11** \| \| --- \| \| **TOTDW** \| | \| 12 \| \| --- \| \| 12 \| | \| 5.7861900 \| \| --- \| \| 130.0266904 \| |

LOC=Mville ENTRY=P10

| **Variable** | **N** | **Std Error** |
| --- | --- | --- |
| \| **DW_F11** \| \| --- \| \| **TOTDW** \| | \| 10 \| \| --- \| \| 10 \| | \| 7.0226467 \| \| --- \| \| 147.8911166 \| |

LOC=Mville ENTRY=P12

| **Variable** | **N** | **Std Error** |
| --- | --- | --- |
| \| **DW_F11** \| \| --- \| \| **TOTDW** \| | \| 11 \| \| --- \| \| 11 \| | \| 4.4816533 \| \| --- \| \| 144.3106417 \| |

LOC=Mville ENTRY=P15

| **Variable** | **N** | **Std Error** |
| --- | --- | --- |
| \| **DW_F11** \| \| --- \| \| **TOTDW** \| | \| 11 \| \| --- \| \| 11 \| | \| 5.0350367 \| \| --- \| \| 73.6514025 \| |

LOC=Mville ENTRY=P19

| **Variable** | **N** | **Std Error** |
| --- | --- | --- |
| \| **DW_F11** \| \| --- \| \| **TOTDW** \| | \| 11 \| \| --- \| \| 11 \| | \| 5.7242167 \| \| --- \| \| 108.3512974 \| |

LOC=Mville ENTRY=P20

| **Variable** | **N** | **Std Error** |
| --- | --- | --- |
| \| **DW_F11** \| \| --- \| \| **TOTDW** \| | \| 12 \| \| --- \| \| 12 \| | \| 2.8613959 \| \| --- \| \| 134.6869493 \| |

LOC=Mville ENTRY=P24

| **Variable** | **N** | **Std Error** |
| --- | --- | --- |
| \| **DW_F11** \| \| --- \| \| **TOTDW** \| | \| 12 \| \| --- \| \| 12 \| | \| 4.9018589 \| \| --- \| \| 82.5808516 \| |

LOC=Mville ENTRY=P25

| **Variable** | **N** | **Std Error** |
| --- | --- | --- |
| \| **DW_F11** \| \| --- \| \| **TOTDW** \| | \| 12 \| \| --- \| \| 12 \| | \| 6.8124146 \| \| --- \| \| 117.7724922 \| |

LOC=Mville ENTRY=P26

| **Variable** | **N** | **Std Error** |
| --- | --- | --- |
| \| **DW_F11** \| \| --- \| \| **TOTDW** \| | \| 12 \| \| --- \| \| 12 \| | \| 8.2617134 \| \| --- \| \| 128.7525084 \| |

LOC=Mville ENTRY=P30

| **Variable** | **N** | **Std Error** |
| --- | --- | --- |
| \| **DW_F11** \| \| --- \| \| **TOTDW** \| | \| 12 \| \| --- \| \| 12 \| | \| 2.3476486 \| \| --- \| \| 60.9097125 \| |

LOC=Mville ENTRY=P31

| **Variable** | **N** | **Std Error** |
| --- | --- | --- |
| \| **DW_F11** \| \| --- \| \| **TOTDW** \| | \| 12 \| \| --- \| \| 12 \| | \| 5.2387396 \| \| --- \| \| 137.7321946 \| |

LOC=Mville ENTRY=P32

| **Variable** | **N** | **Std Error** |
| --- | --- | --- |
| \| **DW_F11** \| \| --- \| \| **TOTDW** \| | \| 12 \| \| --- \| \| 12 \| | \| 4.5386297 \| \| --- \| \| 173.5251139 \| |

LOC=Mville ENTRY=P33

| **Variable** | **N** | **Std Error** |
| --- | --- | --- |
| \| **DW_F11** \| \| --- \| \| **TOTDW** \| | \| 12 \| \| --- \| \| 12 \| | \| 5.6600912 \| \| --- \| \| 137.6279712 \| |

LOC=Mville ENTRY=P34

| **Variable** | **N** | **Std Error** |
| --- | --- | --- |
| \| **DW_F11** \| \| --- \| \| **TOTDW** \| | \| 11 \| \| --- \| \| 11 \| | \| 3.9681388 \| \| --- \| \| 100.3321018 \| |

LOC=Mville ENTRY=P37

| **Variable** | **N** | **Std Error** |
| --- | --- | --- |
| \| **DW_F11** \| \| --- \| \| **TOTDW** \| | \| 12 \| \| --- \| \| 12 \| | \| 2.2400123 \| \| --- \| \| 63.1543069 \| |

LOC=Mville ENTRY=P40

| **Variable** | **N** | **Std Error** |
| --- | --- | --- |
| \| **DW_F11** \| \| --- \| \| **TOTDW** \| | \| 11 \| \| --- \| \| 11 \| | \| 3.8800401 \| \| --- \| \| 159.2351835 \| |

LOC=Mville ENTRY=P41

| **Variable** | **N** | **Std Error** |
| --- | --- | --- |
| \| **DW_F11** \| \| --- \| \| **TOTDW** \| | \| 12 \| \| --- \| \| 12 \| | \| 3.5091818 \| \| --- \| \| 106.2519027 \| |

LOC=Mville ENTRY=P42

| **Variable** | **N** | **Std Error** |
| --- | --- | --- |
| \| **DW_F11** \| \| --- \| \| **TOTDW** \| | \| 12 \| \| --- \| \| 12 \| | \| 2.8537966 \| \| --- \| \| 110.9107267 \| |

LOC=Mville ENTRY=P43

| **Variable** | **N** | **Std Error** |
| --- | --- | --- |
| \| **DW_F11** \| \| --- \| \| **TOTDW** \| | \| 12 \| \| --- \| \| 12 \| | \| 5.4899203 \| \| --- \| \| 106.3169972 \| |

LOC=Mville ENTRY=P44

| **Variable** | **N** | **Std Error** |
| --- | --- | --- |
| \| **DW_F11** \| \| --- \| \| **TOTDW** \| | \| 12 \| \| --- \| \| 12 \| | \| 4.4954237 \| \| --- \| \| 131.0137835 \| |

LOC=Mville ENTRY=P45

| **Variable** | **N** | **Std Error** |
| --- | --- | --- |
| \| **DW_F11** \| \| --- \| \| **TOTDW** \| | \| 12 \| \| --- \| \| 12 \| | \| 4.9542706 \| \| --- \| \| 142.4895323 \| |

LOC=Mville ENTRY=P7G3

| **Variable** | **N** | **Std Error** |
| --- | --- | --- |
| \| **DW_F11** \| \| --- \| \| **TOTDW** \| | \| 12 \| \| --- \| \| 12 \| | \| 4.8051481 \| \| --- \| \| 90.6979865 \| |

LOC=Mville ENTRY=P7G6

| **Variable** | **N** | **Std Error** |
| --- | --- | --- |
| \| **DW_F11** \| \| --- \| \| **TOTDW** \| | \| 12 \| \| --- \| \| 12 \| | \| 5.5223264 \| \| --- \| \| 128.8254956 \| |

LOC=Mville ENTRY=Secar

| **Variable** | **N** | **Std Error** |
| --- | --- | --- |
| \| **DW_F11** \| \| --- \| \| **TOTDW** \| | \| 12 \| \| --- \| \| 12 \| | \| 4.1723556 \| \| --- \| \| 121.3562652 \| |

LOC=Mville ENTRY=Secar78

| **Variable** | **N** | **Std Error** |
| --- | --- | --- |
| \| **DW_F11** \| \| --- \| \| **TOTDW** \| | \| 12 \| \| --- \| \| 12 \| | \| 3.0701679 \| \| --- \| \| 97.5890074 \| |

LOC=Mville ENTRY=T1442

| **Variable** | **N** | **Std Error** |
| --- | --- | --- |
| \| **DW_F11** \| \| --- \| \| **TOTDW** \| | \| 12 \| \| --- \| \| 12 \| | \| 2.4007765 \| \| --- \| \| 56.6785974 \| |

LOC=Mville ENTRY=T1561

| **Variable** | **N** | **Std Error** |
| --- | --- | --- |
| \| **DW_F11** \| \| --- \| \| **TOTDW** \| | \| 10 \| \| --- \| \| 10 \| | \| 2.3133339 \| \| --- \| \| 39.0137879 \| |

LOC=Mville ENTRY=T1772

| **Variable** | **N** | **Std Error** |
| --- | --- | --- |
| \| **DW_F11** \| \| --- \| \| **TOTDW** \| | \| 11 \| \| --- \| \| 10 \| | \| 2.2699633 \| \| --- \| \| 61.7145238 \| |

LOC=Mville ENTRY=T655

| **Variable** | **N** | **Std Error** |
| --- | --- | --- |
| \| **DW_F11** \| \| --- \| \| **TOTDW** \| | \| 10 \| \| --- \| \| 10 \| | \| 5.3123557 \| \| --- \| \| 103.5198575 \| |

LOC=Mville ENTRY=Wahluke

| **Variable** | **N** | **Std Error** |
| --- | --- | --- |
| \| **DW_F11** \| \| --- \| \| **TOTDW** \| | \| 12 \| \| --- \| \| 11 \| | \| 4.0298323 \| \| --- \| \| 66.5720267 \| |

LOC=Mville ENTRY=Whitmar

| **Variable** | **N** | **Std Error** |
| --- | --- | --- |
| \| **DW_F11** \| \| --- \| \| **TOTDW** \| | \| 12 \| \| --- \| \| 12 \| | \| 3.4423314 \| \| --- \| \| 109.1209320 \| |

LOC=Nephi ENTRY=' '

| **Variable** | **N** | **Std Error** |
| --- | --- | --- |
| \| **DW_F11** \| \| --- \| \| **TOTDW** \| | \| 0 \| \| --- \| \| 0 \| | \| . \| \| --- \| \| . \| |

LOC=Nephi ENTRY=Acc1156

| **Variable** | **N** | **Std Error** |
| --- | --- | --- |
| \| **DW_F11** \| \| --- \| \| **TOTDW** \| | \| 10 \| \| --- \| \| 10 \| | \| 4.0301995 \| \| --- \| \| 109.7249115 \| |

LOC=Nephi ENTRY=Acc238

| **Variable** | **N** | **Std Error** |
| --- | --- | --- |
| \| **DW_F11** \| \| --- \| \| **TOTDW** \| | \| 12 \| \| --- \| \| 12 \| | \| 5.3457660 \| \| --- \| \| 70.3786572 \| |

LOC=Nephi ENTRY=Acc243

| **Variable** | **N** | **Std Error** |
| --- | --- | --- |
| \| **DW_F11** \| \| --- \| \| **TOTDW** \| | \| 12 \| \| --- \| \| 12 \| | \| 5.1210703 \| \| --- \| \| 67.2815368 \| |

LOC=Nephi ENTRY=Anatone

| **Variable** | **N** | **Std Error** |
| --- | --- | --- |
| \| **DW_F11** \| \| --- \| \| **TOTDW** \| | \| 11 \| \| --- \| \| 12 \| | \| 5.7109745 \| \| --- \| \| 65.0742388 \| |

LOC=Nephi ENTRY=Boardman

| **Variable** | **N** | **Std Error** |
| --- | --- | --- |
| \| **DW_F11** \| \| --- \| \| **TOTDW** \| | \| 11 \| \| --- \| \| 12 \| | \| 3.0485357 \| \| --- \| \| 49.8250239 \| |

LOC=Nephi ENTRY=Discover

| **Variable** | **N** | **Std Error** |
| --- | --- | --- |
| \| **DW_F11** \| \| --- \| \| **TOTDW** \| | \| 12 \| \| --- \| \| 11 \| | \| 4.2167554 \| \| --- \| \| 124.4924590 \| |

LOC=Nephi ENTRY=E49X

| **Variable** | **N** | **Std Error** |
| --- | --- | --- |
| \| **DW_F11** \| \| --- \| \| **TOTDW** \| | \| 12 \| \| --- \| \| 12 \| | \| 8.3390945 \| \| --- \| \| 171.2316114 \| |

LOC=Nephi ENTRY=E58X

| **Variable** | **N** | **Std Error** |
| --- | --- | --- |
| \| **DW_F11** \| \| --- \| \| **TOTDW** \| | \| 12 \| \| --- \| \| 11 \| | \| 10.4732901 \| \| --- \| \| 139.7586555 \| |

LOC=Nephi ENTRY=Goldar

| **Variable** | **N** | **Std Error** |
| --- | --- | --- |
| \| **DW_F11** \| \| --- \| \| **TOTDW** \| | \| 11 \| \| --- \| \| 12 \| | \| 6.4290333 \| \| --- \| \| 99.3510739 \| |

LOC=Nephi ENTRY=P10

| **Variable** | **N** | **Std Error** |
| --- | --- | --- |
| \| **DW_F11** \| \| --- \| \| **TOTDW** \| | \| 11 \| \| --- \| \| 11 \| | \| 6.5677084 \| \| --- \| \| 67.9944100 \| |

LOC=Nephi ENTRY=P12

| **Variable** | **N** | **Std Error** |
| --- | --- | --- |
| \| **DW_F11** \| \| --- \| \| **TOTDW** \| | \| 9 \| \| --- \| \| 10 \| | \| 5.5639317 \| \| --- \| \| 81.9119972 \| |

LOC=Nephi ENTRY=P15

| **Variable** | **N** | **Std Error** |
| --- | --- | --- |
| \| **DW_F11** \| \| --- \| \| **TOTDW** \| | \| 10 \| \| --- \| \| 10 \| | \| 5.8076957 \| \| --- \| \| 90.3609618 \| |

LOC=Nephi ENTRY=P19

| **Variable** | **N** | **Std Error** |
| --- | --- | --- |
| \| **DW_F11** \| \| --- \| \| **TOTDW** \| | \| 11 \| \| --- \| \| 12 \| | \| 6.8975004 \| \| --- \| \| 118.7677719 \| |

LOC=Nephi ENTRY=P20

| **Variable** | **N** | **Std Error** |
| --- | --- | --- |
| \| **DW_F11** \| \| --- \| \| **TOTDW** \| | \| 12 \| \| --- \| \| 11 \| | \| 6.2849359 \| \| --- \| \| 124.0605600 \| |

LOC=Nephi ENTRY=P24

| **Variable** | **N** | **Std Error** |
| --- | --- | --- |
| \| **DW_F11** \| \| --- \| \| **TOTDW** \| | \| 12 \| \| --- \| \| 12 \| | \| 4.7764526 \| \| --- \| \| 100.9236049 \| |

LOC=Nephi ENTRY=P25

| **Variable** | **N** | **Std Error** |
| --- | --- | --- |
| \| **DW_F11** \| \| --- \| \| **TOTDW** \| | \| 12 \| \| --- \| \| 11 \| | \| 6.4257071 \| \| --- \| \| 95.2054713 \| |

LOC=Nephi ENTRY=P26

| **Variable** | **N** | **Std Error** |
| --- | --- | --- |
| \| **DW_F11** \| \| --- \| \| **TOTDW** \| | \| 12 \| \| --- \| \| 12 \| | \| 9.0172161 \| \| --- \| \| 54.9386190 \| |

LOC=Nephi ENTRY=P30

| **Variable** | **N** | **Std Error** |
| --- | --- | --- |
| \| **DW_F11** \| \| --- \| \| **TOTDW** \| | \| 12 \| \| --- \| \| 12 \| | \| 3.6066122 \| \| --- \| \| 45.5765355 \| |

LOC=Nephi ENTRY=P31

| **Variable** | **N** | **Std Error** |
| --- | --- | --- |
| \| **DW_F11** \| \| --- \| \| **TOTDW** \| | \| 12 \| \| --- \| \| 12 \| | \| 6.8869919 \| \| --- \| \| 121.7329713 \| |

LOC=Nephi ENTRY=P32

| **Variable** | **N** | **Std Error** |
| --- | --- | --- |
| \| **DW_F11** \| \| --- \| \| **TOTDW** \| | \| 12 \| \| --- \| \| 12 \| | \| 7.6113613 \| \| --- \| \| 150.3639795 \| |

LOC=Nephi ENTRY=P33

| **Variable** | **N** | **Std Error** |
| --- | --- | --- |
| \| **DW_F11** \| \| --- \| \| **TOTDW** \| | \| 12 \| \| --- \| \| 12 \| | \| 8.1691913 \| \| --- \| \| 143.8206904 \| |

LOC=Nephi ENTRY=P34

| **Variable** | **N** | **Std Error** |
| --- | --- | --- |
| \| **DW_F11** \| \| --- \| \| **TOTDW** \| | \| 12 \| \| --- \| \| 12 \| | \| 7.4902985 \| \| --- \| \| 147.5076322 \| |

LOC=Nephi ENTRY=P37

| **Variable** | **N** | **Std Error** |
| --- | --- | --- |
| \| **DW_F11** \| \| --- \| \| **TOTDW** \| | \| 11 \| \| --- \| \| 12 \| | \| 2.4950804 \| \| --- \| \| 55.5556818 \| |

LOC=Nephi ENTRY=P40

| **Variable** | **N** | **Std Error** |
| --- | --- | --- |
| \| **DW_F11** \| \| --- \| \| **TOTDW** \| | \| 12 \| \| --- \| \| 12 \| | \| 7.9380893 \| \| --- \| \| 128.6257973 \| |

LOC=Nephi ENTRY=P41

| **Variable** | **N** | **Std Error** |
| --- | --- | --- |
| \| **DW_F11** \| \| --- \| \| **TOTDW** \| | \| 12 \| \| --- \| \| 12 \| | \| 5.2214612 \| \| --- \| \| 119.1803760 \| |

LOC=Nephi ENTRY=P42

| **Variable** | **N** | **Std Error** |
| --- | --- | --- |
| \| **DW_F11** \| \| --- \| \| **TOTDW** \| | \| 12 \| \| --- \| \| 12 \| | \| 6.7655999 \| \| --- \| \| 118.4033591 \| |

LOC=Nephi ENTRY=P43

| **Variable** | **N** | **Std Error** |
| --- | --- | --- |
| \| **DW_F11** \| \| --- \| \| **TOTDW** \| | \| 12 \| \| --- \| \| 12 \| | \| 8.8467992 \| \| --- \| \| 95.9540771 \| |

LOC=Nephi ENTRY=P44

| **Variable** | **N** | **Std Error** |
| --- | --- | --- |
| \| **DW_F11** \| \| --- \| \| **TOTDW** \| | \| 12 \| \| --- \| \| 12 \| | \| 7.2117017 \| \| --- \| \| 159.8800296 \| |

LOC=Nephi ENTRY=P45

| **Variable** | **N** | **Std Error** |
| --- | --- | --- |
| \| **DW_F11** \| \| --- \| \| **TOTDW** \| | \| 12 \| \| --- \| \| 12 \| | \| 6.9602618 \| \| --- \| \| 110.0227836 \| |

LOC=Nephi ENTRY=P7G3

| **Variable** | **N** | **Std Error** |
| --- | --- | --- |
| \| **DW_F11** \| \| --- \| \| **TOTDW** \| | \| 11 \| \| --- \| \| 11 \| | \| 7.1869548 \| \| --- \| \| 105.7705547 \| |

LOC=Nephi ENTRY=P7G6

| **Variable** | **N** | **Std Error** |
| --- | --- | --- |
| \| **DW_F11** \| \| --- \| \| **TOTDW** \| | \| 12 \| \| --- \| \| 12 \| | \| 8.3990081 \| \| --- \| \| 120.1457047 \| |

LOC=Nephi ENTRY=Secar

| **Variable** | **N** | **Std Error** |
| --- | --- | --- |
| \| **DW_F11** \| \| --- \| \| **TOTDW** \| | \| 12 \| \| --- \| \| 11 \| | \| 5.8884654 \| \| --- \| \| 181.0240211 \| |

LOC=Nephi ENTRY=Secar78

| **Variable** | **N** | **Std Error** |
| --- | --- | --- |
| \| **DW_F11** \| \| --- \| \| **TOTDW** \| | \| 12 \| \| --- \| \| 12 \| | \| 4.9790647 \| \| --- \| \| 138.8191352 \| |

LOC=Nephi ENTRY=T1442

| **Variable** | **N** | **Std Error** |
| --- | --- | --- |
| \| **DW_F11** \| \| --- \| \| **TOTDW** \| | \| 12 \| \| --- \| \| 12 \| | \| 1.2376455 \| \| --- \| \| 55.1213319 \| |

LOC=Nephi ENTRY=T1561

| **Variable** | **N** | **Std Error** |
| --- | --- | --- |
| \| **DW_F11** \| \| --- \| \| **TOTDW** \| | \| 12 \| \| --- \| \| 12 \| | \| 1.3967161 \| \| --- \| \| 17.5854923 \| |

LOC=Nephi ENTRY=T1772

| **Variable** | **N** | **Std Error** |
| --- | --- | --- |
| \| **DW_F11** \| \| --- \| \| **TOTDW** \| | \| 12 \| \| --- \| \| 11 \| | \| 2.6134366 \| \| --- \| \| 71.3021264 \| |

LOC=Nephi ENTRY=T655

| **Variable** | **N** | **Std Error** |
| --- | --- | --- |
| \| **DW_F11** \| \| --- \| \| **TOTDW** \| | \| 12 \| \| --- \| \| 11 \| | \| 4.6680553 \| \| --- \| \| 119.2016665 \| |

LOC=Nephi ENTRY=Wahluke

| **Variable** | **N** | **Std Error** |
| --- | --- | --- |
| \| **DW_F11** \| \| --- \| \| **TOTDW** \| | \| 12 \| \| --- \| \| 12 \| | \| 4.3601967 \| \| --- \| \| 74.7843425 \| |

LOC=Nephi ENTRY=Whitmar

| **Variable** | **N** | **Std Error** |
| --- | --- | --- |
| \| **DW_F11** \| \| --- \| \| **TOTDW** \| | \| 12 \| \| --- \| \| 12 \| | \| 5.2157702 \| \| --- \| \| 73.8798107 \| |

LOC=Sharp ENTRY=' '

| **Variable** | **N** | **Std Error** |
| --- | --- | --- |
| \| **DW_F11** \| \| --- \| \| **TOTDW** \| | \| 0 \| \| --- \| \| 0 \| | \| . \| \| --- \| \| . \| |

LOC=Sharp ENTRY=Acc1156

| **Variable** | **N** | **Std Error** |
| --- | --- | --- |
| \| **DW_F11** \| \| --- \| \| **TOTDW** \| | \| 11 \| \| --- \| \| 0 \| | \| 2.1227340 \| \| --- \| \| . \| |

LOC=Sharp ENTRY=Acc238

| **Variable** | **N** | **Std Error** |
| --- | --- | --- |
| \| **DW_F11** \| \| --- \| \| **TOTDW** \| | \| 12 \| \| --- \| \| 0 \| | \| 3.3383972 \| \| --- \| \| . \| |

LOC=Sharp ENTRY=Acc243

| **Variable** | **N** | **Std Error** |
| --- | --- | --- |
| \| **DW_F11** \| \| --- \| \| **TOTDW** \| | \| 11 \| \| --- \| \| 0 \| | \| 2.4100113 \| \| --- \| \| . \| |

LOC=Sharp ENTRY=Anatone

| **Variable** | **N** | **Std Error** |
| --- | --- | --- |
| \| **DW_F11** \| \| --- \| \| **TOTDW** \| | \| 11 \| \| --- \| \| 0 \| | \| 3.6828386 \| \| --- \| \| . \| |

LOC=Sharp ENTRY=Boardman

| **Variable** | **N** | **Std Error** |
| --- | --- | --- |
| \| **DW_F11** \| \| --- \| \| **TOTDW** \| | \| 12 \| \| --- \| \| 0 \| | \| 2.7366718 \| \| --- \| \| . \| |

LOC=Sharp ENTRY=Discover

| **Variable** | **N** | **Std Error** |
| --- | --- | --- |
| \| **DW_F11** \| \| --- \| \| **TOTDW** \| | \| 12 \| \| --- \| \| 0 \| | \| 2.2876653 \| \| --- \| \| . \| |

LOC=Sharp ENTRY=E49X

| **Variable** | **N** | **Std Error** |
| --- | --- | --- |
| \| **DW_F11** \| \| --- \| \| **TOTDW** \| | \| 11 \| \| --- \| \| 0 \| | \| 2.9018966 \| \| --- \| \| . \| |

LOC=Sharp ENTRY=E58X

| **Variable** | **N** | **Std Error** |
| --- | --- | --- |
| \| **DW_F11** \| \| --- \| \| **TOTDW** \| | \| 12 \| \| --- \| \| 0 \| | \| 4.1303293 \| \| --- \| \| . \| |

LOC=Sharp ENTRY=Goldar

| **Variable** | **N** | **Std Error** |
| --- | --- | --- |
| \| **DW_F11** \| \| --- \| \| **TOTDW** \| | \| 12 \| \| --- \| \| 0 \| | \| 1.1254470 \| \| --- \| \| . \| |

LOC=Sharp ENTRY=P10

| **Variable** | **N** | **Std Error** |
| --- | --- | --- |
| \| **DW_F11** \| \| --- \| \| **TOTDW** \| | \| 12 \| \| --- \| \| 0 \| | \| 3.4800743 \| \| --- \| \| . \| |

LOC=Sharp ENTRY=P12

| **Variable** | **N** | **Std Error** |
| --- | --- | --- |
| \| **DW_F11** \| \| --- \| \| **TOTDW** \| | \| 11 \| \| --- \| \| 0 \| | \| 3.3261908 \| \| --- \| \| . \| |

LOC=Sharp ENTRY=P15

| **Variable** | **N** | **Std Error** |
| --- | --- | --- |
| \| **DW_F11** \| \| --- \| \| **TOTDW** \| | \| 12 \| \| --- \| \| 0 \| | \| 3.2577423 \| \| --- \| \| . \| |

LOC=Sharp ENTRY=P19

| **Variable** | **N** | **Std Error** |
| --- | --- | --- |
| \| **DW_F11** \| \| --- \| \| **TOTDW** \| | \| 12 \| \| --- \| \| 0 \| | \| 2.2337716 \| \| --- \| \| . \| |

LOC=Sharp ENTRY=P20

| **Variable** | **N** | **Std Error** |
| --- | --- | --- |
| \| **DW_F11** \| \| --- \| \| **TOTDW** \| | \| 12 \| \| --- \| \| 0 \| | \| 1.9944487 \| \| --- \| \| . \| |

LOC=Sharp ENTRY=P24

| **Variable** | **N** | **Std Error** |
| --- | --- | --- |
| \| **DW_F11** \| \| --- \| \| **TOTDW** \| | \| 12 \| \| --- \| \| 0 \| | \| 2.5135715 \| \| --- \| \| . \| |

LOC=Sharp ENTRY=P25

| **Variable** | **N** | **Std Error** |
| --- | --- | --- |
| \| **DW_F11** \| \| --- \| \| **TOTDW** \| | \| 12 \| \| --- \| \| 0 \| | \| 3.4983047 \| \| --- \| \| . \| |

LOC=Sharp ENTRY=P26

| **Variable** | **N** | **Std Error** |
| --- | --- | --- |
| \| **DW_F11** \| \| --- \| \| **TOTDW** \| | \| 12 \| \| --- \| \| 0 \| | \| 3.0597795 \| \| --- \| \| . \| |

LOC=Sharp ENTRY=P30

| **Variable** | **N** | **Std Error** |
| --- | --- | --- |
| \| **DW_F11** \| \| --- \| \| **TOTDW** \| | \| 12 \| \| --- \| \| 0 \| | \| 2.2763157 \| \| --- \| \| . \| |

LOC=Sharp ENTRY=P31

| **Variable** | **N** | **Std Error** |
| --- | --- | --- |
| \| **DW_F11** \| \| --- \| \| **TOTDW** \| | \| 12 \| \| --- \| \| 0 \| | \| 2.4137339 \| \| --- \| \| . \| |

LOC=Sharp ENTRY=P32

| **Variable** | **N** | **Std Error** |
| --- | --- | --- |
| \| **DW_F11** \| \| --- \| \| **TOTDW** \| | \| 12 \| \| --- \| \| 0 \| | \| 2.4440740 \| \| --- \| \| . \| |

LOC=Sharp ENTRY=P33

| **Variable** | **N** | **Std Error** |
| --- | --- | --- |
| \| **DW_F11** \| \| --- \| \| **TOTDW** \| | \| 11 \| \| --- \| \| 0 \| | \| 3.9808711 \| \| --- \| \| . \| |

LOC=Sharp ENTRY=P34

| **Variable** | **N** | **Std Error** |
| --- | --- | --- |
| \| **DW_F11** \| \| --- \| \| **TOTDW** \| | \| 11 \| \| --- \| \| 0 \| | \| 2.3865326 \| \| --- \| \| . \| |

LOC=Sharp ENTRY=P37

| **Variable** | **N** | **Std Error** |
| --- | --- | --- |
| \| **DW_F11** \| \| --- \| \| **TOTDW** \| | \| 12 \| \| --- \| \| 0 \| | \| 1.5205098 \| \| --- \| \| . \| |

LOC=Sharp ENTRY=P40

| **Variable** | **N** | **Std Error** |
| --- | --- | --- |
| \| **DW_F11** \| \| --- \| \| **TOTDW** \| | \| 11 \| \| --- \| \| 0 \| | \| 4.5073548 \| \| --- \| \| . \| |

LOC=Sharp ENTRY=P41

| **Variable** | **N** | **Std Error** |
| --- | --- | --- |
| \| **DW_F11** \| \| --- \| \| **TOTDW** \| | \| 12 \| \| --- \| \| 0 \| | \| 1.6921071 \| \| --- \| \| . \| |

LOC=Sharp ENTRY=P42

| **Variable** | **N** | **Std Error** |
| --- | --- | --- |
| \| **DW_F11** \| \| --- \| \| **TOTDW** \| | \| 12 \| \| --- \| \| 0 \| | \| 2.1278023 \| \| --- \| \| . \| |

LOC=Sharp ENTRY=P43

| **Variable** | **N** | **Std Error** |
| --- | --- | --- |
| \| **DW_F11** \| \| --- \| \| **TOTDW** \| | \| 11 \| \| --- \| \| 0 \| | \| 3.6058776 \| \| --- \| \| . \| |

LOC=Sharp ENTRY=P44

| **Variable** | **N** | **Std Error** |
| --- | --- | --- |
| \| **DW_F11** \| \| --- \| \| **TOTDW** \| | \| 11 \| \| --- \| \| 0 \| | \| 2.1363436 \| \| --- \| \| . \| |

LOC=Sharp ENTRY=P45

| **Variable** | **N** | **Std Error** |
| --- | --- | --- |
| \| **DW_F11** \| \| --- \| \| **TOTDW** \| | \| 12 \| \| --- \| \| 0 \| | \| 2.6066151 \| \| --- \| \| . \| |

LOC=Sharp ENTRY=P7G3

| **Variable** | **N** | **Std Error** |
| --- | --- | --- |
| \| **DW_F11** \| \| --- \| \| **TOTDW** \| | \| 11 \| \| --- \| \| 0 \| | \| 1.9548726 \| \| --- \| \| . \| |

LOC=Sharp ENTRY=P7G6

| **Variable** | **N** | **Std Error** |
| --- | --- | --- |
| \| **DW_F11** \| \| --- \| \| **TOTDW** \| | \| 11 \| \| --- \| \| 0 \| | \| 4.9009279 \| \| --- \| \| . \| |

LOC=Sharp ENTRY=Secar

| **Variable** | **N** | **Std Error** |
| --- | --- | --- |
| \| **DW_F11** \| \| --- \| \| **TOTDW** \| | \| 12 \| \| --- \| \| 0 \| | \| 4.0180923 \| \| --- \| \| . \| |

LOC=Sharp ENTRY=Secar78

| **Variable** | **N** | **Std Error** |
| --- | --- | --- |
| \| **DW_F11** \| \| --- \| \| **TOTDW** \| | \| 12 \| \| --- \| \| 0 \| | \| 3.0758124 \| \| --- \| \| . \| |

LOC=Sharp ENTRY=T1442

| **Variable** | **N** | **Std Error** |
| --- | --- | --- |
| \| **DW_F11** \| \| --- \| \| **TOTDW** \| | \| 11 \| \| --- \| \| 0 \| | \| 2.8763670 \| \| --- \| \| . \| |

LOC=Sharp ENTRY=T1561

| **Variable** | **N** | **Std Error** |
| --- | --- | --- |
| \| **DW_F11** \| \| --- \| \| **TOTDW** \| | \| 11 \| \| --- \| \| 0 \| | \| 1.6360713 \| \| --- \| \| . \| |

LOC=Sharp ENTRY=T1772

| **Variable** | **N** | **Std Error** |
| --- | --- | --- |
| \| **DW_F11** \| \| --- \| \| **TOTDW** \| | \| 12 \| \| --- \| \| 0 \| | \| 2.4168830 \| \| --- \| \| . \| |

LOC=Sharp ENTRY=T655

| **Variable** | **N** | **Std Error** |
| --- | --- | --- |
| \| **DW_F11** \| \| --- \| \| **TOTDW** \| | \| 11 \| \| --- \| \| 0 \| | \| 2.4336917 \| \| --- \| \| . \| |

LOC=Sharp ENTRY=Wahluke

| **Variable** | **N** | **Std Error** |
| --- | --- | --- |
| \| **DW_F11** \| \| --- \| \| **TOTDW** \| | \| 12 \| \| --- \| \| 0 \| | \| 2.3081435 \| \| --- \| \| . \| |

LOC=Sharp ENTRY=Whitmar

| **Variable** | **N** | **Std Error** |
| --- | --- | --- |
| \| **DW_F11** \| \| --- \| \| **TOTDW** \| | \| 11 \| \| --- \| \| 0 \| | \| 2.6505777 \| \| --- \| \| . \| |

Graphical Abstract Text:

Plant materials can be characterized for adaptation to various potential restoration environments through prior testing. This may be more predictive than surrogate site measures of geographical, climatic, or soils data.
